# Pathogen evolution in finite populations: slow and steady spreads the best

**DOI:** 10.1098/rsif.2018.0135

**Published:** 2018-10-03

**Authors:** Todd L. Parsons, Amaury Lambert, Troy Day, Sylvain Gandon

**Affiliations:** 1 Laboratoire de Probabilités, Statistique et Modélisation (LPSM), Sorbonne Université, CNRS UMR 8001, Paris, France; 2 Center for Interdisciplinary Research in Biology (CIRB), Collège de France, PSL Research University, CNRS UMR 7241, INSERM U1050, Paris, France; 3 Department of Mathematics and Statistics, Queen’s University, Kingston, Canada; 4 Department of Biology, Queen’s University, Kingston, Canada; 5 Centre d’Ecologie Fonctionnelle et Evolutive (CEFE), Université de Montpellier–Université Paul-Valéry Montpellier–EPHE, CNRS UMR 5175, Montpellier, France

**Keywords:** epidemiology, virulence, life-history evolution, genetic drift, demographic stochasticity, Adaptive Dynamics

## Abstract

The theory of life-history evolution provides a powerful framework to understand the evolutionary dynamics of pathogens. It assumes, however, that host populations are large and that one can neglect the effects of demographic stochasticity. Here, we expand the theory to account for the effects of finite population size on the evolution of pathogen virulence. We show that demographic stochasticity introduces additional evolutionary forces that can qualitatively affect the dynamics and the evolutionary outcome. We discuss the importance of the shape of the pathogen fitness landscape on the balance between mutation, selection and genetic drift. This analysis reconciles Adaptive Dynamics with population genetics in finite populations and provides a new theoretical toolbox to study life-history evolution in realistic ecological scenarios.

## 1. Introduction

Why are some pathogens virulent and harm their hosts while others have minimal effect on host fitness? Our ability to understand and predict the evolutionary dynamics of pathogen virulence has considerable implications for public-health management [1–3]. A classical explanation for pathogen virulence involves trade-offs with other pathogen life-history traits. If certain components of pathogen fitness, such as a high transmission rate or a low clearance rate, necessarily require that the pathogen incidentally increase host mortality, then virulence is expected to evolve [4]. A now classical way to develop specific predictions from this hypothesis is invasion analysis and evolutionary game theory, under assumptions that have been since formalized as Adaptive Dynamics [1,4–6]. This approach relies on the assumption that the mutation rate is small so that the epidemiological dynamics occur on a faster timescale than the evolutionary dynamics [4,7–9]. Under simple epidemiological assumptions (e.g. well-mixed population, no co-infection or superinfection with different genotypes, a single infection pathway, etc.) the evolutionarily stable level of virulence maximizes the basic reproduction ratio *R*_0_ of the pathogen [7,10,11], but see e.g. [12–17] for more complex epidemiological scenarios.

The above-mentioned theory allows one to determine the level of virulence expected to evolve under a broad range of epidemiological scenarios but it still suffers from the fundamental shortcoming of being a deterministic theory. The number of infected individuals, however, can be very small (e.g. at the onset of an epidemic or after a vaccination campaign) and demographic stochasticity—i.e. randomness in individual mortality and reproduction [18]—is likely to affect both the epidemiological and evolutionary dynamics of the disease. If all that such stochasticity did was to introduce random noise, then the predictions of deterministic theory would likely suffice. However, several recent studies have demonstrated that this is not the case. For example, [19,20] each used different theoretical approaches to demonstrate that finite population size tends to select for lower virulence and transmission, using perturbation series, and assuming fixed numbers of infecteds, respectively, to estimate fixation probabilities. Likewise, [21] analysed the effect of finite population size in a complex epidemiological model with unstable epidemiological dynamics and showed that finite population size could induce an evolutionary instability that may either lead to selection for very high or very low transmission.

Taken together, the existing literature presents a complex picture of the factors that drive virulence evolution and it remains unclear how all of these factors are related to one another and how they might interact. In this paper, we develop a very general theory of pathogen evolution that can be used to examine virulence evolution when the above-mentioned factors are at play. First, we use an individual-based description of the epidemiological process to derive a stochastic characterization of the evolutionary epidemiology dynamics of the pathogen. This theoretical framework is used to pinpoint the effect of finite population size on the interplay between epidemiology and evolution. Second, we analyse this model under the realistic assumption that the rate of mutation is small, so that pathogen evolution can be approximated by a sequence of fixations. We derive the probability of fixation of a mutant pathogen under both weak and strong selection regimes, and for different epidemiological scenarios. Third, we use this theoretical framework to derive the stationary distribution of pathogen virulence resulting from the balance between mutation, selection and genetic drift. This yields new predictions regarding the effect of the shape of pathogen fitness landscape and the size of the population on the long-term evolution of the pathogen. As the question of virulence evolution can be viewed as a specific example of the more general notion of life-history evolution [22,23], our results should be directly applicable to other life-history traits and other organisms as well, providing a new theoretical approach for studying life-history evolution in realistic ecological scenarios based on the principles advocated in [24]: Stochastic Adaptive Dynamics (SAD).

## 2. Model

We use a classical SIR epidemiological model with demography, where hosts can either be susceptible, infected or recovered. The number of each of these types of hosts is denoted by *N*_S_, *N*_I_ and *N*_R_, respectively. Because we are interested in the effect of demographic stochasticity the model is derived from a microscopic description of all the events that may occur in a finite—but not fixed—host population of total size *N*_T_ = *N*_S_ + *N*_I_ + *N*_R_ living in an area of size *n* (the details of the model are given in the electronic supplementary material).

We use *λ* to denote the rate at which new susceptible hosts enter the population *per unit area*, and therefore the total rate is given by *λn*. We focus on the case of frequency-dependent transmission; i.e. new infections occur at rate (*β*/*N*_T_)*N*_S_*N*_I_, where *β* is a constant quantifying the combined effects of contact rate among individuals and the probability of pathogen transmission, given an appropriate contact occurs. Note, however, that other forms of transmission (e.g. density-dependent transmission [25]) yield qualitatively similar results [26]. We also assume that already infected hosts cannot be reinfected by another pathogen strain (i.e. no co-infections). All hosts are assumed to suffer a constant *per capita* death rate of *δ*, whereas infected hosts die *due to disease* at *per capita* rate *α* and they recover at *per capita* rate *γ*. Finally, to study pathogen evolution, we need to introduce genetic variation in the pathogen population. Therefore, we consider *d* pathogen strains which differ in their transmission rate *β*_*i*_, and virulence *α*_*i*_, with *i* ∈ {1, …, *d*}. Likewise, we use the subscripted variable *N*_I_*i*__ to denote the number of hosts infected with strain *i*.

The above assumptions give a continuous-time Markov process tracking the number of individuals of each type of host. To progress in the analysis, we use a diffusion approximation and work with host densities defined as *S* = *N*_S_/*n*, *I*_*i*_ = *N*_I_*i*__/*n* and *N* = *N*_T_/*n* and we define the total density of infected hosts as 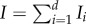. When *n* is sufficiently large (but finite) these variables can be approximated using a continuous state space and so this model can be described by a system of stochastic differential equations (see electronic supplementary material, §3).

### 2.1. Deterministic evolution

In the limit where the habitat size (and thus the host population size) becomes infinite, demographic stochasticity becomes unimportant and the epidemiological dynamics are given by the following system of ordinary differential equations:
2.1
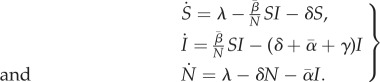
The bars above *α* and *β* refer to the mean of the transmission rate and the virulence distributions of the infected host population (i.e. 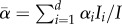). In the absence of the pathogen, the density of hosts equilibrates at *S*_0_ = *λ*/*δ*. A monomorphic pathogen (*d* = 1, 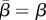 and 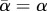) is able to invade this equilibrium if its basic reproduction ratio, *R*_0_ = *β*/(*δ* + *α* + *γ*) is greater than one. If this condition is fulfilled, then the system reaches an endemic equilibrium, where *S*_eq_/*N*_eq_ = 1/*R*_0_, *I*_eq_/*N*_eq_ = (*δ*/(*δ* + *γ*))(1 − 1/*R*_0_) and *N*_eq_ = (*λ*(*δ* + *γ*)/*δ*(*β* − *α*))*R*_0_.

When several strains are present in the population, the evolutionary dynamics of the pathogen can be tracked with [27,28]:
2.2
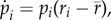
where *p*_*i*_ = *I*_*i*_/*I* is the frequency of hosts infected with strain *i*. The quantity *r*_*i*_ = *β*_*i*_(*S*/*N*) − (*δ* + *α*_*i*_ + *γ*) is the instantaneous *per capita* growth rate of strain *i* and 
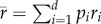
 is the average *per capita* growth rate of the infected host population. When *d* = 2 only two strains are competing (a wild-type, strain 1, and a mutant, strain 2) and the change in the frequency *p*_2_ of the mutant strain is given by:
2.3
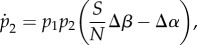
where Δ*β* = *β*_2_ − *β*_1_ and Δ*α* = *α*_2_ − *α*_1_ are the effects of the mutation on transmission and virulence, respectively.

**Figure 1.**
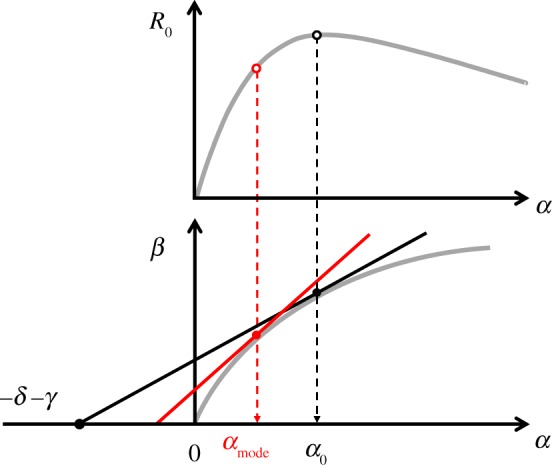
Schematic of the effect of finite population size on the evolution of pathogen virulence. The grey line in the top figure represents the effect of pathogen virulence, *α*, on *R*_0_ (for an asymmetric fitness function). The grey line in the bottom figure represents the effect of pathogen virulence, *α*, on pathogen transmission, *β*. In the deterministic version of our model, the marginal value theorem can be used to find the optimal pathogen virulence, *α*_0_ (dashed black arrow). In this model, optimal virulence maximizes *R*_0_ in the absence of demographic stochasticity. Finite population size modifies selection and favours pathogen strategies with lower virulence (see equation (3.4)). The mode of the stationary distribution of pathogen virulence is indicated by a dashed red arrow, *α*_mode_ (see equation (3.7)). This geometrical construction indicates that finite population size is expected to favour *slower* strains even if they have a lower *R*_0_.

The above formalization can be used to understand the evolution of pathogen life history under different scenarios. First, under the classical Adaptive Dynamics assumption that the mutation rate is very small, one may use a separation of timescales where the epidemiological dynamics reach an endemic equilibrium (set by the resident pathogen, strain 1) before the introduction of a new variant (strain 2) by mutation. In this case, evolution favours the strain with the highest basic reproduction ratio, *R*_0,*i*_ = *β*_*i*_/(*δ* + *α*_*i*_ + *γ*). In other words, evolution favours strains with higher transmission rates and lower virulence. According to the trade-off hypothesis, however, transmission and virulence cannot evolve independently. For example, the within-host growth rate of pathogens is likely to affect both traits and result in a functional trade-off between transmission and virulence [4,7–9]. Under this assumption, equation (2.3) can be used to predict the evolutionary stable virulence strategy (figure 1).^1^ The above model can also be used to predict virulence evolution when the evolutionary and epidemiological dynamics occur on a similar timescale [27–29]. For instance, these models can be used to understand virulence evolution during an epidemic [4,30–32]. In this case, a pathogen strain *i* with a lower *R*_0_ may outcompete other strains if its instantaneous growth rate, *r*_*i*_, is higher.

### 2.2. Stochastic evolution

Finite population size introduces demographic stochasticity and the epidemiological dynamics can be described by the following system of (Itô) stochastic differential equations:
2.4
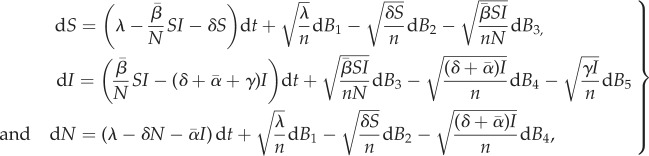
where *B*_1_, …, *B*_5_ are independent Brownian motions. As expected, when 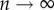 this set of stochastic differential equations reduces to the deterministic equations in (2.1) (n.b., both (2.4) and (2.1) require that one knows the strain frequencies, as given by (2.2) or (2.5) below, respectively, for a complete description of the dynamics).

In finite populations, the pathogen, and indeed the host population itself, are destined to extinction with probability 1. The time it takes for this to occur, however, depends critically on the parameter values. For example, in a host population infected with a monomorphic pathogen (i.e. *d* = 1), if *R*_0_ is larger than 1 the size of the infected host population reaches a quasi-stationary distribution which is approximately normal. The mean of this distribution is of order *n* and its standard deviation is of order 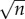 [33,34]. The extinction time from the quasi-stationary distribution increases exponentially with *n* [33,34], and so, in the remainder of the paper, we will assume that *n* is large enough so that we can focus on the dynamics conditional on non-extinction.

As in the deterministic case, one can study evolutionary dynamics by focusing on the change in strain frequencies. We obtain a stochastic differential equation analogous to (2.2) (see electronic supplementary material, §4):
2.5
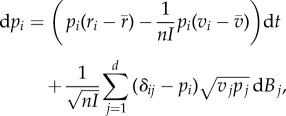
where *v*_*i*_ = *β*_*i*_(*S*/*N*) + (*δ* + *α*_*i*_ + *γ*) is the variance in the growth rate of strain *i* (while *r*_*i*_ = *β*_*i*_(*S*/*N*) − (*δ* + *α*_*i*_ + *γ*) is the mean) and 
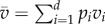
 is the average variance in growth rate of the infected host population. The first (advective, d*t*) component in equation (2.5) is analogous to (2.2). The second (diffusive, d*B*) component shows that finite population size (i.e. when the pathogen-infected population size, as measured by the total number of infected hosts, *nI* is not too large) can affect the direction of evolution. In contrast with the deterministic model, the evolutionary dynamics are not driven exclusively by the expected growth rate *r*_*i*_, but also by a minimization of the variance. This effect is akin to bet-hedging theory stating that a mutant strategy with lower variance in reproduction may outcompete a resident strategy with a higher average instantaneous growth rate [35,36]. To better understand this effect, it is particularly insightful to examine the case *d* = 2 when only two strains are competing and the change in frequency *p*_2_ of the mutant strain is given by:
2.6
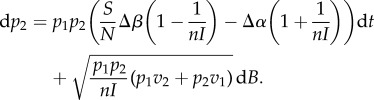
The first component (the advective component) in equation (2.6) is similar to (2.3) except for the 1/*nI* terms. Those terms are due to the fact that a transmission (or a death) event of the mutant is associated with a change in the number of mutants as well as an increase (decrease) of the total infected host population size by one individual. This concomitant variation of infected host population size affects the effective change of the mutant frequency (relative to the change expected under the deterministic model where the population size is assumed to be infinite). This effect decreases the benefit associated with higher transmission and increases the cost of virulence. In the long-term, this effect (the first term in (2.5)) is thus expected to select for lower virulence. But this long-term evolutionary outcome cannot be described by an evolutionary stable state because demographic stochasticity is also expected to generate noise (the diffusion term in (2.5)). Indeed, this stochasticity (i.e. genetic drift) may lead to the invasion and fixation of strains with lower *per capita* growth rates. In the following, we fully characterize this complex evolutionary outcome with the stationary distribution of pathogen virulence under different epidemiological scenarios.

## 3. Results

The above theoretical framework embodied by the stochastic differential equations (2.4) and (2.5) subsume the deterministic model and can be used to study the interplay of all the relevant factors affecting virulence evolution. In the following, we will assume that pathogen mutation is rare, so that evolution can be described, as in classical Adaptive Dynamics, as a chain of fixations of new pathogen mutations. In contrast with Adaptive Dynamics, however, demographic stochasticity in the resident population may allow neutral, or even mildly deleterious, mutations to go to fixation. The analysis of the effect of finite population size requires specific ways to quantify the stochastic fate of a genotype [37]. To determine the fate of a new mutation we need to compute the probability of fixation of a mutant pathogen in a resident population. In the absence of selection, the fixation probability of a mutant allele depends only on the demography of the population. When the size of the population is fixed and equal to *N* the fixation probability of a neutral allele is 1/*N*. When the fixation probability of a mutant is higher than neutral it indicates that the mutant is selectively favoured. This is particularly useful in many complex situations where the interplay between selection and genetic drift are difficult to disentangle like time-varying demography [38,39] or spatial structure [40]. In our model, the difficulty arises from (i) the stochastic demography of the infected host population and (ii) the fact that pathogen life-history traits feedback on the epidemiological dynamics and thus on the intensity of genetic drift.

### 3.1. Stationary distribution of pathogen virulence at equilibrium

Here we assume, as in the Adaptive Dynamics framework, that the pathogen mutation rate *μ* is so low that the mutant pathogen (strain 2) arises when the resident population (strain 1) has reached a quasi-stationary distribution tightly peaked about *nI*_eq_ (i.e. close to the endemic equilibrium derived in the deterministic model). The *R*_0_ of the two strains may be written in the following way: *R*_0,2_ = *R*_0,1_(1 + *s*) where *s* measures the magnitude of selection.

When selection is strong (i.e. *s*≫1/*n*) the probability of fixation of the mutant when *N*_I_2__(0) mutants with *R*_0,2_ > 1 are introduced into a resident population at equilibrium is (see electronic supplementary material, §5.2):
3.1
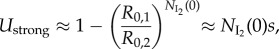
which may be obtained by approximating the invading strain by a branching process (see electronic supplementary material, §8.2 for a rigorous justification).

**Figure 2.**
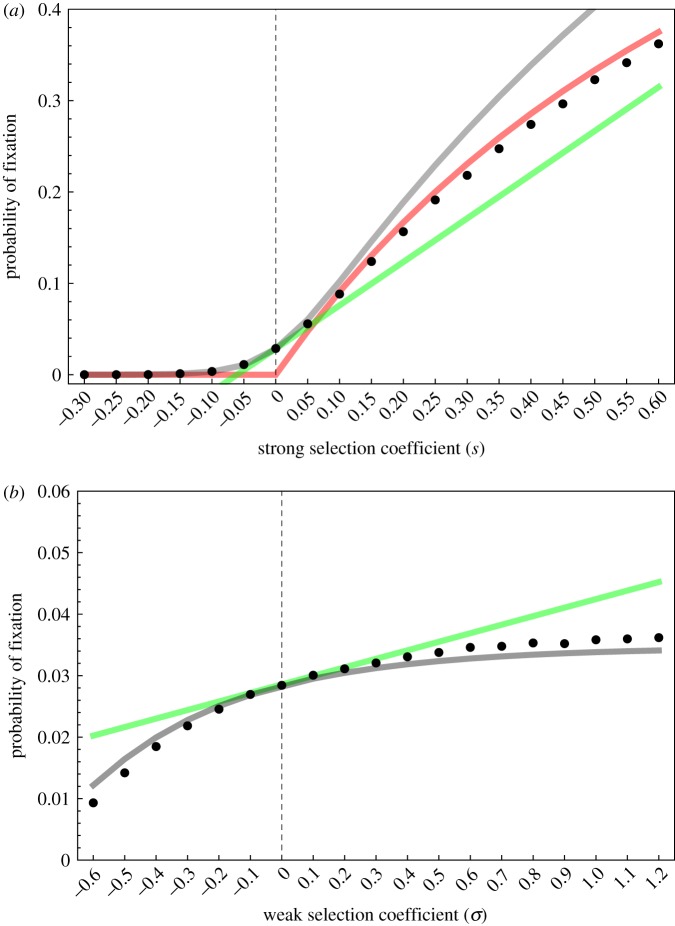
Probability of fixation for (*a*) different values of *s* (strong selection effect) and (*b*) different values of *σ* for fixed *R*_0_ (weak selection effect). Simulation results for the model described in §2 are indicated with a dot, weak selection approximation is indicated with a grey line and its linear approximation (equation (3.2)) is indicated with a green line, the strong selection approximation is indicated with a red line (equation (3.1)). Parameter values of the resident population: *n* = 100, *R*_0_ = 4, *δ* = 1, *α* = 3, *γ* = 1, *λ* = 2, *β*_1_ = 20. For the simulation, a single mutant (an individual host infected with a mutant pathogen) is introduced at the endemic equilibrium set by the resident pathogen: *S*_eq_ = 24 and *I*_eq_ = 35. 10^6^ simulations are realized for each parameter values and we plot the proportion of the simulations where the mutant goes to fixation. We implement strong selection by setting *β*_1_ = *β*_2_(1 + *s*) so that *R*_0,1_ = *R*_0,2_(1 + *s*), and weak selection by setting *β*_1_ = *β*_2_(1 + *σ*), while holding *R*_0,1_ = *R*_0,2_ by setting *α*_2_ = (*δ* + *α*_1_ + *γ*)/(1 + *σ*) − *δ* − *γ*.

When the mutant and the resident have similar values of *R*_0_ > 1 (i.e. *s* is of order 1/*n*) selection is weak, and the derivation of the probability of fixation is a much more difficult problem. The classical population genetics approach under the assumption that population size is fixed (or is characterized by a deterministic trajectory independent of mutant frequency) is to use the diffusion equation of mutant frequency to derive the probability of fixation [38,39]. But in our model, equation (2.3) is not autonomous and is coupled with the epidemiological dynamics. To derive the probability of fixation we use a separation of timescale argument to reduce the dimension of the system (see [41] for a discussion of the approach). Indeed, if selection is weak, as *n* → ∞, the deterministic component of the model sends the system rapidly to the endemic equilibrium, which is now a manifold of fixed points, on which coexistence is possible at all mutant frequencies. After this, it is possible to approximate the change in frequency of the mutant by tracking the dynamics of the projection of the mutant frequency on this manifold (see electronic supplementary material, §5.3). This one-dimensional system can then be used to derive the probability of fixation under weak selection. A first-order approximation in *s* and *σ* is:
3.2
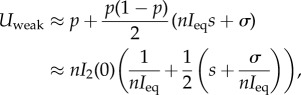
where *p* = *I*_2_(0)/*I*_eq_ and *σ* = (*β*_1_ − *β*_2_)/*β*_2_. The first term in (3.2) is the probability of fixation of a single neutral mutation introduced in an infected host population at the endemic equilibrium, *nI*_eq_. The second term takes into account the effect due to selection. First, selection may be driven by differences in *R*_0_. Second, even if strains have identical *R*_0_ (i.e. *s* = 0) selection may be driven by *σ* which measures the difference in transmission rate; this effect selects for lower transmission rates, and, since under weak selection the *R*_0_ values are approximately equal, for lower virulence. Note, however, that the effect of *s* rapidly overwhelms the effect of *σ* as the infected host population size *nI*_eq_ becomes large (unless *s* is of order 1/*n*). The probability of fixation given in (3.2) confirms that evolution tends to push towards higher basic reproductive ratio but when the population size is small other forces may affect the evolutionary outcome. In particular, when *nI*_eq_ is small, strains with lower *R*_0_ can reach fixation. Figure 2 shows the result of stochastic simulations that confirm the approximations (3.1) and (3.2) under different epidemiological scenarios, and show that our approximations already perform extremely well for populations as small as the order of 100 hosts (see electronic supplementary material, §7 for details of the simulations).

**Figure 3.**
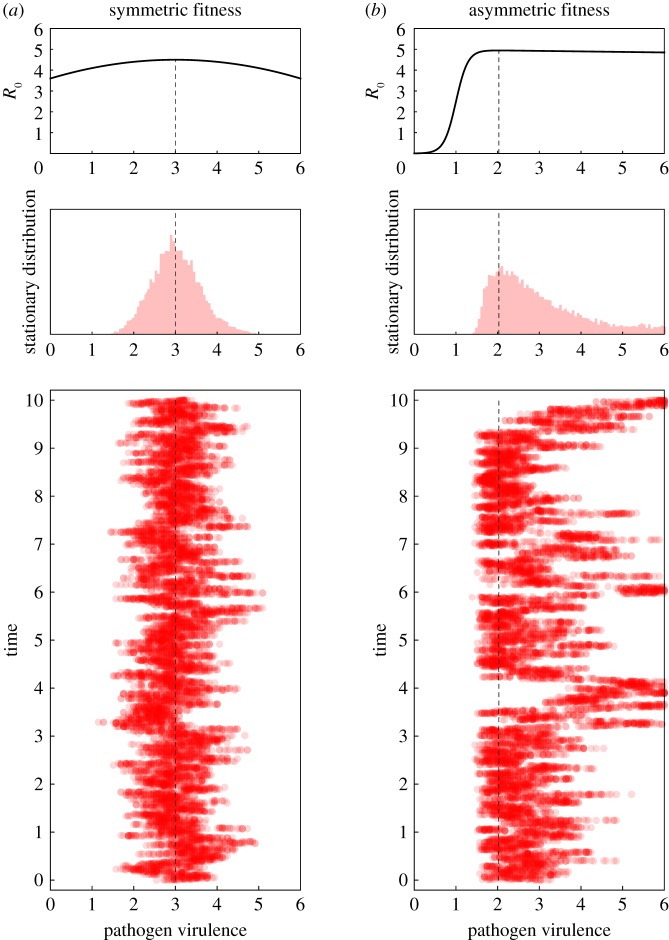
Dynamics of pathogen virulence across time (one time unit on the graph is 10^7^ time steps in the simulation) and stationary distribution of pathogen virulence for two different fitness landscapes: (*a*) symmetric fitness landscape with *β*(*α*) = (*δ* + *γ* + *α*)*R*_0,max_(1 − *w*(*α*_0_ − *α*)^2^), *R*_0,max_ = 4.5 and *α*_0_ = 3, (*b*) asymmetric fitness landscape with 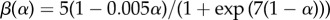. We take 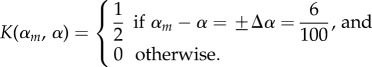 The dashed vertical line indicates the position of *α*_0_. Other parameter values: *n* = 200, *δ* = 1, *α* = 3, *γ* = 1, *λ* = 2, *μ* = 0.001.

Even though the probability of fixation helps understand the interplay between selection and genetic drift it does not account for any differences in the time to fixation and it is often difficult to measure this probability experimentally as well (but see [42]). What may be more accessible is a characterization of the phenotypic state of the population across different points in time (or in space among replicate populations)—that is, the stationary distribution of the virulence of the pathogen under the action of mutation, selection and genetic drift [43–45] (figure 3).

To derive the stationary distribution of pathogen virulence, we first need to impose a trade-off between virulence and transmission rate, setting *β* = *β*(*α*), and introduce the mutation kernel *K*(*α*_*m*_, *α*), the probability that a mutant with strategy *α*_*m*_ appears in a monomorphic population with strategy *α*. Here, we assume that this distribution has mean equal to the current resident trait value and variance *ν*. Under the assumption that the mutation rate *μ* remains small, pathogen polymorphism is limited to the transient period between the introduction of a mutant and a fixation, and we may consider the (monomorphic) resident virulence as a random process evolving in time.

The probability of fixation (3.2) accurately describes the direction of evolution, and the evolution of pathogen virulence can then be described by the following Fokker–Planck diffusion equation (see electronic supplementary material, §6):
3.3
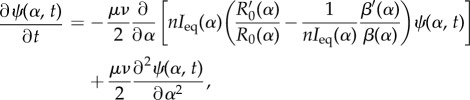
where *ψ*(*α*, *t*) is the probability of observing pathogen virulence *α* at time *t* and ′ indicates the derivative with respect to *α*, and we write *R*_0_(*α*) and *I*_eq_(*α*) to emphasize that these quantities depend on the resident virulence. The first term of the above equation indicates a strong deterministic trend, with *R*′_0_(*α*) indicating a trend towards a higher basic reproduction ratio, offset by a finite population effect proportional to −*β*′(*α*) that tends to select for lower transmission. Under the classical assumption that pathogen transmission and pathogen virulence are linked by a genetic trade-off one can ask what the level of pathogen virulence is where the advective term is zero. This trait value corresponds to the mode of the stationary distribution of pathogen virulence and is given by the following condition (see electronic supplementary material, equation S.39):
3.4
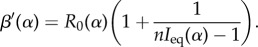
When the infected host population is very large (i.e. 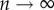) we recover the marginal value theorem, while finite population size increases the slope *β*′(*α*) and reduces the mode of the stationary distribution (figure 1). Thus, provided the transmission–virulence trade-off function is concave, finite population size is expected to decrease virulence and transmission rates. In other words, pathogen avirulence may be viewed as a bet-hedging strategy because even if it reduces the instantaneous growth rate *r*_*i*_, the reduced variance in growth rate *v*_*i*_ is adaptive in finite populations.

**Figure 4.**
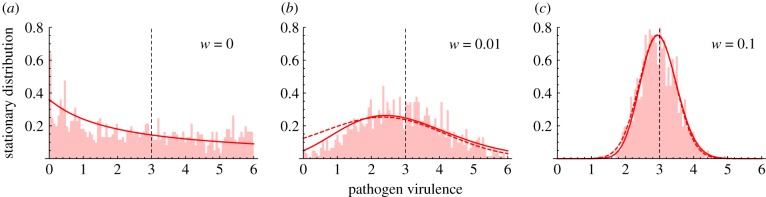
Stationary distribution for symmetric fitness landscapes with increasing strength of selection around the optimum with *β*(*α*) = (*δ* + *γ* + *α*)*R*_0,max_(1 − *w*(*α*_0_ − *α*)^2^), *K*(*α*_*m*_, *α*) as in figure 3, and *α*_0_ = 3 for three different values of *w*: (*a*) *w* = 0, (*b*) 0.01 and (*c*) 0.1. Note that when *w* = 0, the fitness landscape is flat, whereas with increasing *w*, the landscape becomes more tightly peaked at the optimum. The light red histogram indicates results of a stochastic simulation. The red line indicates the stationary distribution of the diffusion approximation (the dashed line indicates the approximation of this distribution, see (3.6)). The dashed vertical line indicates the position of *α*_0_. Parameter values: *n* = 200, *R*_0,max_ = 4, *d* = 1, *α* = 3, *γ* = 1, *λ* = 2, *μ* = 0.001.

Let us now consider the limiting case when all the pathogen strains have the same *R*_0_. This corresponds to a very special case where the fitness landscape is flat. The deterministic model predicts that pathogen life-history variation is neutral near the endemic equilibrium (see (2.2)). The probability of fixation (3.2) shows, however, that selection is *quasi-neutral* and favours pathogens with lower transmission and virulence rates [19,20,26,46]. The stationary distribution results from the balance between selection (pushing towards lower values of pathogen traits) and mutation (reintroducing variation). If we focus on virulence and allow variation between the minimal viable value *α*_min_ and the maximal viable value *α*_max_, the stationary distribution is (see electronic supplementary material, equation S.33):
3.5
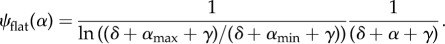
It is worth noting that this distribution is independent of the pathogen-infected population size. Indeed, near the endemic equilibrium, when pathogens have the same *R*_0_, the probability of fixation (3.2) is independent of infected population size. So this prediction holds even in very large populations. The time to fixation may, however, be considerably longer in large populations and the assumption that polymorphism is always reduced to the resident and a single mutant may not always hold as the population size increases. Yet, stochastic simulations confirm that (3.5) correctly predicts the stationary distribution, which is relatively insensitive to the infected population size, but varies with *δ* + *γ* (figure 4*a*).

Second, we consider a general fitness landscape with a single maximum. It is possible to derive a good approximation for the stationary distribution (see electronic supplementary material, S.38):
3.6
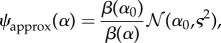
where *α*_0_ is the virulence that maximizes *R*_0_, 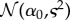 is the Gaussian distribution with mean *α*_0_ and variance ς^2^ = 1/(*nI*_eq_(*α*_0_)|*R*_0_′′(*α*_0_)|/*R*_0_(*α*_0_)), and *nI*_eq_(*α*_0_) is the expected number of infected individuals at the endemic equilibrium when the virulence is *α*_0_. We thus see the effect of demographic stochasticity is to bias the Gaussian, putting more weight on values of the virulence below *α*_0_: if *R*_0_(*α*_0_) ≥ *R*_0_(*α*), then, *β*(*α*_0_)/*β*(*α*) ≥ (*δ* + *α*_0_ + *γ*)/(*δ* + *α* + *γ*) > 1 for *α* < *α*_0_. This becomes more clear when we consider the mode and mean of the (true) stationary distribution (see electronic supplementary material, §6.3):
3.7
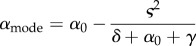
and
3.8
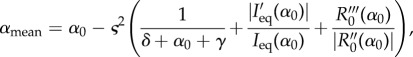
respectively. Equations (3.7) and (3.8) indicate that, as expected from the simple optimization approach used above in (3.4) and illustrated in figures 3 and 4, a lower infected population size tends to decrease pathogen virulence. However, the above derivation of the stationary distribution goes far beyond this optimization criterion. First, it accurately predicts the mode of the stationary distribution; in particular, it shows that the peakedness of the fitness landscape may affect the mode of the stationary distribution. The skew of the fitness landscape can also have huge effects on the stationary distribution (figure 3): a positive skew leads to a higher mean virulence and may thus counteract the effect of a small pathogen-infected population. In other words, whether demographic stochasticity favours lower or higher virulence also depends on the shape of the fitness landscape. Second, our analysis predicts the amount of variation one may expect to see around this mode. Unlike the criteria used to derive a single optimal strategy, our approach predicts accurately the expected variation around this mode (figures 3 and 4). Note that the population remains monomorphic most of the time (because mutation is assumed to be small) but the variance of the stationary distribution refers to the distribution of phenotypes explored through time (or through space if stochastic evolution is taking place in multiple isolated populations).

## 4. Discussion

Evolutionary theory has led to the development of different mathematical tools for studying phenotypic evolution in a broad diversity of ecological scenarios [47–49]. For instance, Adaptive Dynamics is a powerful theoretical framework to study life-history evolution when mutation is assumed to be rare so that demographic and evolutionary processes can be decoupled [5,6]. This analysis yields evolutionarily stable life-history strategies and captures the ultimate outcome of evolution. This approach, however, relies on the assumption that population size is infinite and that the epidemiological trajectory is deterministic. Finite population size, however, can also affect evolutionary trajectories. In particular, even the fittest genotype can be invaded by a deleterious mutant when population size is reduced. This leads to the collapse of the concept of evolutionarily stable strategy. On the other hand, population genetics allows us to consider the effect of finite population size and drift, but at the cost of assuming fixed population sizes and ignoring ecological processes (however, see, e.g. [38,50,51]). Here we build upon Stochastic Adaptive Dynamics (SAD) [43,45,49], a new theoretical framework where the evolutionary outcome of life-history evolution is studied by systematically scaling from ecologically complex individual-based stochastic models to the stationary distribution of the phenotype under mutation–selection–drift equilibrium. Under the assumption that the mutation rate is small, the long-term stochastic dynamics and equilibrium distribution can be derived from a diffusion approximation. In contrast with previous population genetics models, the present framework also allows life-history evolution to affect population size and, consequently, the amount of demographic stochasticity. In other words, this framework retains key strengths of Adaptive Dynamics but relaxes a major assumption by allowing genetic drift to affect the evolutionary outcome (see also [52], p. 1149). As such, our SAD framework is an important step towards a better integration between Adaptive Dynamics and classical population genetics.

We show that finite population size induces a selective pressure towards strains with lower variance in growth rate (but see also [35,39,46]). A simple way to understand this effect is to compare the fate of two strains with the same *R*_0_ but with different life-history strategies. The *fast* strain is very transmissible but has a short duration of infection (e.g. because of high virulence or high clearance rate). The *slow* strain has a long duration of infection but a small transmission rate. As the two strains have the same *R*_0_, deterministic models predict that these two strains should coexist once common, but that neither can invade from small numbers. With finite population size, however, invasion is possible. When population size is assumed to be fixed, say *N*, the two strains are forced to share the same speed because when e.g. one strain infects a new host, the artificial constraint on the pathogen population size requires the death of a host infected by the other strain. By contrast, when population size is allowed to vary stochastically, the competing strains can have different speeds. The fast strain has a higher extinction rate simply because more events happen per unit of time: rare events, such as large fluctuations, will happen more regularly for the faster strain. As in Aesop's fable, ‘slow and steady wins the race’: when population size is allowed to vary stochastically, however, the race has no finish line (unlike a fixed population model, you cannot hit *N* and ‘win’) and a strain can succeed only by outlasting its competitors. The advantage thus falls to the slower strain which persists by using longer infectious periods to ‘wait out’ periods of paucity of susceptibles, outliving the more volatile fast strain.

Previous studies [19,20] pointed out the influence of finite population size on the direction of virulence evolution, but they focused mainly on the quasi-neutral case where all the strains have the same *R*_0_. Humplik *et al*. [20] did look at scenarios where strains have different *R*_0_, but assumed a fixed population, strong selection, and values of *R*_0_ so large that all hosts are infected. None of these studies considered varying strengths of selection, and none provided a derivation of the stationary distribution at mutation–selection–drift equilibrium, which describes the long-term behaviour of pathogen virulence, that we believe is key to explore the interaction between finite population size and phenotypic evolution. This distribution yields testable predictions on the mean as well as other moments of the phenotypic distribution.

The approximation (3.6) shows that this distribution is moulded by two main parameters: (i) the pathogen fitness landscape, and (ii) the effective size of the infected host population. First, the pathogen fitness at the endemic equilibrium can be derived from (2.5) and depends mainly on the way *R*_0_ varies with pathogen life-history traits. Under the classical transmission–virulence assumption, *R*_0_ is maximized for some intermediate virulence. But the shape of the trade-off also affects the shape of the fitness landscape and in particular its symmetry. When the fitness landscape of the pathogen is symmetric, reducing the infected population size increases the variance of the stationary distribution but also decreases the mean (and the mode) of this distribution. This effect results from the selection for a reduction of the variance identified in (2.5). This is the effect that emerges in the quasi-neutral case. When the fitness landscape is flat, this may lead to an important bias towards lower virulence (figure 4). When the fitness landscape of the pathogen is asymmetric the skewness of the fitness landscape can affect the mean of the stationary distribution when the equilibrium host population size, *nI*_eq_, is reduced. More specifically, negative (positive) skewness reduces (increases) the mean of the stationary distribution. It is interesting to note that classical functions used to model the trade-off between virulence and transmission tend to generate positive skewness in the fitness landscape [4,8,14]. The asymmetry of these fitness functions may thus counteract the effects of stochasticity *per se* identified in symmetric fitness landscapes. In other words, predictions on the stochastic evolutionary outcome are sensitive to the shape of genetic constraints acting on different pathogen life-history traits. This result is very similar to the deterministic effects discussed in [53,54] on the influence of asymmetric fitness landscapes on phenotypic evolution. Note, however, that the effect analysed by [54] is driven by environmental effects on phenotypes. In our model, we did not assume any environmental effects, and a given genotype is assumed to produce a single phenotype.

While we considered the standard SIR model, our approach can be generalized to consider a number of variants, including the SIRS model, the SEIR model, models with multiple exposed and infected compartments, etc. Our strong selection results for the fixation probability will apply whenever invasion implies fixation [55] (this assumption is also necessary in general to derive (3.3); in particular, the diffusion approximation can fail if e.g. there is an evolutionary branching, such as in a model with co-infection [17]). Multi-type branching processes [56] would allow the addition of e.g. exposed classes, whereas general (non-Markovian) branching processes would allow the consideration of arbitrary distributions for the infectious period [57–59]. Weak selection results may be obtained when the exchange of stability between resident and invader results in a manifold of equilibria connecting the steady states. The structure of the SIR model considered here lends itself to computing the reduced diffusion (2.5); a general, albeit computation-heavy, method to derive the reduced equation is presented in [41]. If, on the other hand, multiple strains could coexist at a stable node or focus or a saddle point for the infinite population limit, then one would have to use large deviations theory or adapt the results of [60], respectively. An important extension would be to consider models with less variance in the infectious period than the exponential distribution considered here, as they could diminish the effects of demographic stochasticity.

We analysed the effects of demographic stochasticity induced by finite population size but environmental stochasticity may also affect evolution [36,61,62]. Environmental factors are known to have dramatic impacts on pathogen transmission and it would thus be particularly relevant to expand the current framework to account for the effects of random perturbations of the environment on pathogen evolution [63]. Indeed, although we focused our analysis on the stationary distribution at the endemic equilibrium of the classical SIR model, we can also explore the effect of demographic stochasticity on the transient evolutionary dynamics away from the endemic equilibrium, e.g. in epidemic scenarios, under bottlenecks, etc. [64].

Further, to focus on the effects of finite populations, we have considered a well-mixed population, whereas it is well known that spatial spreading can facilitate coexistence of competing strains and trade-offs between pathogen virulence and host mobility [65–67]. Moreover, spatial structure can result in smaller local effective population sizes, thus amplifying the effects of demographic stochasticity. Other factors may reduce the effective infected host population size as well. For instance, variance in transmission among infected hosts is likely to reduce the effective infected population size below *nI*_eq_. One source of heterogeneity in transmissibility may be induced by public-health interventions (e.g. vaccination, drug treatments), but intrinsic behavioural or immunological heterogeneities among hosts may induce superspreading transmission routes as well [68,69]. As such, a structured stochastic model would be an important extension.

Another possible extension of this model would be to analyse the effect of demographic stochasticity on the multi-locus dynamics of pathogens. Indeed, the interaction between genetic drift and selection is known to yield complex evolutionary dynamics resulting in the build-up of negative linkage disequilibrium between loci. But the analysis of this so-called Hill–Robertson effect is often restricted to population genetics models with fixed population size. The build-up of linkage disequilibrium in some epidemiological models has been discussed in some simulation models [70,71]. Our model provides a theoretical framework to explore the effect of finite population size on multi-locus dynamics of pathogens and to generate more accurate predictions on e.g. the evolution of drug resistance [72].

Finally, although we have presented our results in the context of pathogen evolution, it is hopefully clear that a very similar theoretical framework could be used to study other examples of life-history evolution in the context of demographic stochasticity. Current general life-history theory largely neglects the evolutionary consequences of stochasticity arising from small population sizes. Our results suggest that it would be profitable to determine what sorts of insights might be gained for life-history evolution more generally by using the type of theoretical framework developed here.

## Supplementary Material

Supplementary Information

## Endnote

1 Equation (2.3) shows us that a rare mutant can invade if and only if Δ*β*/Δ*α* > *N*/*S* = *R*_0_ i.e. if the line through (−*δ* −*γ*, 0) with slope Δ*β*/Δ*α* lies above the curve (*α*, *β*(*α*)) at the resident virulence. If *β*(*α*) is concave, then for all *α*, Δ*β*/Δ*α* ≤ *β*′(*α*); in particular, if the line through (−*δ* −*γ*, 0) is tangent to the curve at *α*_0_, then no mutant can invade, and *α*_0_ is an evolutionary stable strategy; see [73].

## Data accessibility

Mathematica simulation code is available on request.
