# Supplementary material for: Pathogen evolution in finite populations: slow and steady spreads the best
Source: J R Soc Interface. 2018 Oct 3;15(147):20180135. doi: 10.1098/rsif.2018.0135 (PMC6228476; doi:10.1098/rsif.2018.0135)
Supplement: Supplementary Information [file rsif20180135supp1.pdf]

Supplementary Information

Pathogen evolution in finite populations: slow and steady spreads the best

Todd L. Parsons, Amaury Lambert, Troy Day and Sylvain Gandon

September 11, 2018

Contents

1 Introduction **3**

2 A Stochastic Epidemiological Model with Multiple Pathogen Strains **3**

3 Stochastic Differential Equation Formulation **5**
3.1 Obtaining Equation (4) 9

4 Itô's Formula and Derivation of Equation (5) **10**

5 Probability of Fixation of a Mutant Pathogen **13**
5.1 A Deterministic Limit and its Asymptotic Analysis 13
5.2 The Strong Selection Case 15
5.3 The Weak Selection Case 17
5.3.1 Computing the derivatives of $\boldsymbol{\pi}$ 24
5.3.2 Reduced Diffusion 26
5.3.3 Frequency Process 27
5.3.4 Results for $d = 2$ 29
5.4 Reconciling the Strong and Weak Selection Results 32

6 Adaptive Dynamics **36**
6.1 Stationary Distribution 40
6.2 Asymptotic Approximation to Stationary Distribution 41
6.3 Mean & Mode of the Stationary Distribution 44
6.3.1 Estimating the Mean 44
6.3.2 Estimating the Mode 45

7 Some Remarks Regarding Simulations **47**

8 Some Rigorous Demonstrations **48**
8.1 Proof of Proposition 2 48
8.2 Proof of Proposition 5 50
8.2.1 Macroscopic Initial Frequencies 50
8.2.2 Novel Strain in Small Number of Copies 58
8.3 Proof of Proposition 10 60

Glossary of Notation

n	system size
$\lambda^{(n)}n$	immigration rate for susceptible individuals; $\lambda^{(n)} = \lambda + \mathcal{O}\big(\frac{1}{n}\big)$
$\delta^{(n)}$	base mortality rate; $\delta^{(n)} = \delta + \mathcal{O}\big(\frac{1}{n}\big)$
$\beta_i^{(n)}$	contact rate for strain i; $\beta_i^{(n)} = \beta_i + \mathcal{O}\big(\frac{1}{n}\big)$
$\alpha_i^{(n)}$	excess mortality for strain i; $\alpha_i^{(n)} = \alpha_i + \mathcal{O}\big(\frac{1}{n}\big)$
$\gamma_i^{(n)}$	recovery rate for strain i; $\gamma_i^{(n)} = \gamma_i + \mathcal{O}\big(\frac{1}{n}\big)$
$R_{0,i}$	$\frac{\beta_i}{\delta+\alpha_i+\gamma_i}$
$R_{0,i}^{(n)}$	$\frac{\beta_i^{(n)}}{\delta^{(n)}+\alpha_i^{(n)}+\gamma_i^{(n)}} = R_{0,i}\left(1+\frac{r_i}{n}\right) + o\left(\frac{1}{n}\right)$
$S^{(n)}(t)$	number of susceptible individuals at time t
$I_i^{(n)}(t)$	number of individuals infected with strain i at time t
$R^{(n)}(t)$	number of recovered individuals at time t
$N^{(n)}(t)$	total number of individuals at time t
$P_i^{(n)}(t)$	frequency of individuals infected with strain i at time t
$\boldsymbol{I}^{(n)}(t)$	$(I_1^{(n)}(t),\dots,I_d^{(n)}(t),N^{(n)}(t))$
$\boldsymbol{E}^{(n)}(t)$	$(S^{(n)}(t),I_1^{(n)}(t),\dots,I_d^{(n)}(t))$
$\bar{S}^{(n)}(t)$	density of susceptible individuals at time t
$\bar{I}_i^{(n)}(t)$	density of individuals infected with strain i at time t
$\bar{R}^{(n)}(t)$	density of recovered individuals at time t
$\bar{N}^{(n)}(t)$	total density of individuals at time t
$\bar{\boldsymbol{I}}^{(n)}(t)$	$(\bar{I}_i^{(n)}(t),\dots,\bar{I}_d^{(n)}(t))$
$\bar{\boldsymbol{E}}^{(n)}(t)$	$(\bar{S}^{(n)}(t),\bar{I}_i^{(n)}(t),\dots,\bar{I}_d^{(n)}(t),\bar{N}^{(n)}(t))$
$\hat{S}^{(n)}(t)$	density of susceptible individuals at time nt
$\hat{I}_i^{(n)}(t)$	density of individuals infected with strain i at time nt
$\hat{R}^{(n)}(t)$	density of recovered individuals at time nt
$\hat{N}^{(n)}(t)$	total density of individuals at time nt
$\hat{\boldsymbol{I}}^{(n)}(t)$	$(\hat{I}_1^{(n)}(t),\dots,\hat{I}_d^{(n)}(t))$
$\hat{\boldsymbol{E}}^{(n)}(t)$	$(\hat{S}^{(n)}(t),\hat{I}_1^{(n)}(t),\dots,\hat{I}_d^{(n)}(t),\hat{N}^{(n)}(t))$
$S(t)$	asymptotic density of susceptible individuals at time t
$I_i(t)$	asymptotic density of individuals infected with strain i at time t
$R(t)$	asymptotic density of recovered individuals at time t
$N(t)$	total asymptotic density of individuals at time t
$\boldsymbol{E}^{\star,i}$	endemic equilibrium with resident strain i
$\boldsymbol{I}(t)$	$(I_1(t),\dots,I_d(t))$
$\boldsymbol{E}(t)$	$(S(t),I_1(t),\dots,I_d(t),N(t))$
$\hat{S}(t)$	asymptotic density of susceptible individuals in slow time limit
$\hat{I}_i(t)$	asymptotic density of individuals infected with strain i in slow time limit
$\hat{R}(t)$	asymptotic density of recovered individuals in slow time limit
$\hat{N}(t)$	asymptotic total density of individuals in slow time limit
$\hat{\boldsymbol{I}}(t)$	$(\hat{I}_1(t),\dots,\hat{I}_d(t))$
$\hat{\boldsymbol{E}}(t)$	$(\hat{S}(t),\hat{I}_1(t),\dots,\hat{I}_d(t),\hat{N}(t))$
$P_i(t)$	asymptotic frequency of individuals infected with strain i in slow time limit

1 Introduction

In this SI, we derive the results in the main text. Where suitable references exist in the literature, we keep the discussion informal, sketching how the results are obtained and referring to the appropriate references for rigorous proofs. Where they do not, we first give a heuristic derivation for a broader audience, whilst deferring the proofs to the end.

2 A Stochastic Epidemiological Model with Multiple Pathogen Strains

We consider a family of random processes $\left(S^{(n)}(t), I_1^{(n)}(t), \ldots, I_d^{(n)}(t), R^{(n)}(t)\right)$, indexed by a parameter n, the "system size" [31], which plays a role similar to the census population size in population genetics (see *e.g.,* [15, 11, 13]): it can be thought of as the area in which the population lives, determining the population density per unit area and the rate of immigration. Similarly to fixed-size population genetic models, we will consider the asymptotic behaviour of our model when n is large. $S^{(n)}(t)$, $I_1^{(n)}(t), \ldots, I_d^{(n)}(t)$, and $R^{(n)}(t)$ are the number of susceptible individuals, individuals infected with strain $i = 1, \ldots, d$, and recovered individuals, respectively. We will write $N^{(n)}(t)$ for the total population size at time t, so that

$$N^{(n)}(t) = S^{(n)}(t) + I_1^{(n)}(t) + \cdots + I_d^{(n)}(t) + R^{(n)}(t).$$

Using this notation, our compartmental model for the epidemic is represented graphically in Figure S.1.

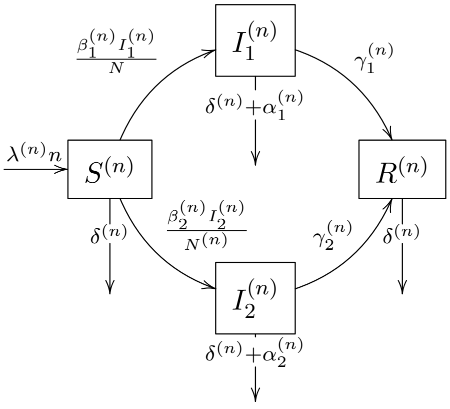

Figure S.1: Compartmental model of a two-strain SIR epidemic. Arrows indicate transitions between states and are labelled with the corresponding transition rate. Arrows into empty space indicate deaths.

Equivalently, we may describe our model as a continuous-time Markov chain $\boldsymbol{E}$ taking values in $\mathbb{N}^{d+2}$ with transition rates given in Table 1. When a transition occurs at time t, we will distinguish

Transition	Rate
$S \to S+1$	$\lambda^{(n)} n$
$S \to S-1$	$\delta^{(n)} S$
$S \to S-1, I_k \to I_k + 1$	$\frac{\beta_k^{(n)} S I_k}{N}$
$I_k \to I_k - 1$	$(\delta^{(n)} + \alpha_k^{(n)}) I_k$
$I_k \to I_k - 1, R \to R+1$	$\gamma_k^{(n)} I_k$
$R \to R-1$	$\delta^{(n)} R$

Table 1: Transition rates from the state $S^{(n)}(t) = S$, $I_k^{(n)}(t) = I_k$ and $R^{(n)}(t) = R$

between the value $\boldsymbol{E}(t-)$ of the Markov chain before the transition and its value $\boldsymbol{E}(t)$ after the transition.

All parameters given in Table 1 may depend on n, but are assumed to have a constant value to first approximation in n

$$\alpha_i^{(n)} = \alpha_i + \mathcal{O}\big(\tfrac{1}{n}\big), \quad \beta_i^{(n)} = \beta_i + \mathcal{O}\big(\tfrac{1}{n}\big), \quad \gamma_i^{(n)} = \gamma_i + \mathcal{O}\big(\tfrac{1}{n}\big), \quad \delta^{(n)} = \delta + \mathcal{O}\big(\tfrac{1}{n}\big), \quad \lambda^{(n)} = \lambda + \mathcal{O}\big(\tfrac{1}{n}\big) \tag{S.1}$$

Simple calculations using the master equation tell us that in the absence of infected individuals, the expected value of $N^{(n)}(t)$ is

$$\mathbb{E}\left[N^{(n)}(t)\right] = e^{-\delta^{(n)} t} N^{(n)}(0) + \frac{\lambda^{(n)}}{\delta^{(n)}} \left(1 - e^{-\delta^{(n)} t}\right) n,$$

which approaches an equilibrium value of $\frac{\lambda^{(n)}}{\delta^{(n)}} n$ as $t \to \infty$. Thus, to first approximation, the total population size is proportional to n.

If one knows the values of $S^{(n)}(t)$ and $\boldsymbol{I}^{(n)}(t) := (I_1^{(n)}(t), \ldots, I_d^{(n)}(t))$, then given one of $R^{(n)}(t)$ or $N^{(n)}(t)$ one can determine the other. For our purposes, it is more convenient to track the total population size, and consider the epidemic

$$\boldsymbol{E}^{(n)}(t) := \left(S^{(n)}(t), \boldsymbol{I}^{(n)}(t), N^{(n)}(t)\right).$$

In what follows, rather than working with $\boldsymbol{E}^{(n)}(t)$ we will focus on the rescaled process

$$\bar{S}^{(n)}(t) := \frac{1}{n} S^{(n)}(t), \quad \bar{I}_i^{(n)}(t) := \frac{1}{n} I_i^{(n)}(t), \quad \bar{N}^{(n)}(t) := \frac{1}{n} N^{(n)}(t),$$

and

$$\bar{\boldsymbol{E}}^{(n)}(t) := \left(\bar{S}^{(n)}(t), \bar{\boldsymbol{I}}^{(n)}(t), \bar{N}^{(n)}(t)\right).$$

$\bar{\boldsymbol{E}}^{(n)}(t)$ has the advantage of being a density dependent population process [21, 22, 23, 24] as generalized in [28]: the transition rates in (1) depend only on the densities $\bar{S}^{(n)}(t), \bar{\boldsymbol{I}}^{(n)}(t), \bar{N}^{(n)}(t)$ and not on the absolute numbers of individuals. As we discuss below, density dependent population processes have a number of nice features, including a law of large numbers and central limit

theorems.

Remark 1. To simplify our subsequent use of subscripts, we will consider $\bar{\boldsymbol{E}}^{(n)}(t)$ as a process taking values in $\mathbb{R}^{d+2}$, the space of points

$$\boldsymbol{x} = (x_0, x_1, \ldots, x_d, x_{d+1}),$$

and use $\bar{S}^{(n)}(t)$ and $\bar{E}_0^{(n)}(t)$, *etc.* interchangeably.

3 Stochastic Differential Equation Formulation

Here, we introduce a very convenient way of writing our Markov chain as the solution to a stochastic integral equation with the help of simple Poisson processes.

A Poisson process P is a Markov process making jumps of $+1$ exclusively, and such that $P(0) = 0$. A Poisson process P is a called a simple Poisson process if it jumps at constant rate 1. In this case, $(P(at))$ is a Poisson process with rate a. This can be generalized by noting that $(P(\int_0^t a(s)\, ds))$ is a time-inhomogeneous Poisson process which jumps at rate $a(t)$ at time t. Similarly, there is a unique continuous-time Markov chain X satisfying

$$X(t) = x_0 + P\left(\int_0^t f(X(s-))\, ds\right)$$

and when $X(t-) = x$, X jumps to $x+1$ at rate $f(x)$.

Then it is not difficult to extend this (see Chapter 6, §4 in [14] for details) to our Markov process as follows:

$$\begin{aligned}
S^{(n)}(t) = {} & S^{(n)}(0) + P_{\boldsymbol{e}_0+\boldsymbol{e}_{d+1}}(n\lambda^{(n)}t) \\
& - P_{-\boldsymbol{e}_0-\boldsymbol{e}_{d+1}}\left(\int_0^t \delta^{(n)} S^{(n)}(s)\, ds\right) - \sum_{i=1}^d P_{-\boldsymbol{e}_0+\boldsymbol{e}_i}\left(\int_0^t \frac{\beta_i^{(n)} S^{(n)}(s) I_i^{(n)}(s)}{N^{(n)}(s)}\, ds\right) \\
I_i^{(n)}(t) = {} & I_i^{(n)}(0) + P_{-\boldsymbol{e}_0+\boldsymbol{e}_i}\left(\int_0^t \frac{\beta_i^{(n)} S^{(n)}(s) I_i^{(n)}(s)}{N^{(n)}(s)}\, ds\right) \\
& - P_{-\boldsymbol{e}_i-\boldsymbol{e}_{d+1}}\left(\int_0^t (\delta^{(n)} + \alpha_i^{(n)}) I_i^{(n)}(s)\, ds\right) - P_{-\boldsymbol{e}_i}\left(\int_0^t \gamma_i^{(n)} I_i^{(n)}(s)\, ds\right) \\
N^{(n)}(t) = {} & N^{(n)}(0) + P_{\boldsymbol{e}_0+\boldsymbol{e}_{d+1}}(n\lambda^{(n)}t) - P_{-\boldsymbol{e}_0-\boldsymbol{e}_{d+1}}\left(\int_0^t \delta^{(n)} S^{(n)}(s)\, ds\right) \\
& - P_{-\boldsymbol{e}_{d+1}}\left(\int_0^t \delta^{(n)}\left(N^{(n)}(s) - \sum_{i=1}^d I_i^{(n)}(s) - S^{(n)}(s)\right) ds\right) \\
& \qquad - \sum_{i=1}^d P_{-\boldsymbol{e}_i-\boldsymbol{e}_{d+1}}\left(\int_0^t (\delta^{(n)} + \alpha_i^{(n)}) I_i^{(n)}(s)\, ds\right).
\end{aligned}$$

where all the processes $P_{\boldsymbol{l}}(t)$ are independent, simple Poisson processes, indexed by the corresponding jumps, $\boldsymbol{l}$, of the Markov process $(\boldsymbol{E}^{(n)}(t))$ and $\boldsymbol{e}_i$ is the ith standard basis vector, the element

of $\mathbb{R}^{d+2}$ with zeros everywhere except for a 1 at row i *i.e.,* an immigration event is indexed by $\boldsymbol{e}_0 + \boldsymbol{e}_{d+1}$, as it increases the number of susceptibles and the total population size by 1.

Changing variables, we get

$$
\begin{aligned}
\bar{S}^{(n)}(t) = \ & \bar{S}^{(n)}(0) + \frac{1}{n} P_{\boldsymbol{e}_0+\boldsymbol{e}_{d+1}}(n\lambda^{(n)} t) \\
& - \frac{1}{n} P_{-\boldsymbol{e}_0-\boldsymbol{e}_{d+1}} \left(n \int_0^t \delta^{(n)} \bar{S}^{(n)}(s)\, ds \right) - \sum_{i=1}^d \frac{1}{n} P_{-\boldsymbol{e}_0+\boldsymbol{e}_i} \left(n \int_0^t \frac{\beta_i^{(n)} \bar{S}^{(n)}(s) \bar{I}_i^{(n)}(s)}{\bar{N}^{(n)}(s)}\, ds \right) \\
\bar{I}_i^{(n)}(t) = \ & \bar{I}_i^{(n)}(0) + \frac{1}{n} P_{-\boldsymbol{e}_0+\boldsymbol{e}_i} \left(n \int_0^t \frac{\beta_i^{(n)} \bar{S}^{(n)}(s) \bar{I}_i^{(n)}(s)}{\bar{N}^{(n)}(s)}\, ds \right) \\
& - \frac{1}{n} P_{-\boldsymbol{e}_i-\boldsymbol{e}_{d+1}} \left(n \int_0^t (\delta^{(n)} + \alpha_i^{(n)}) \bar{I}_i^{(n)}(s)\, ds \right) - \frac{1}{n} P_{-\boldsymbol{e}_i} \left(n \int_0^t \gamma_i^{(n)} \bar{I}_i^{(n)}(s)\, ds \right) \\
\bar{N}^{(n)}(t) = \ & \bar{N}^{(n)}(0) + \frac{1}{n} P_{\boldsymbol{e}_0+\boldsymbol{e}_{d+1}}(n\lambda^{(n)} t) - \frac{1}{n} P_{-\boldsymbol{e}_0-\boldsymbol{e}_{d+1}} \left(n \int_0^t \delta^{(n)} \bar{S}^{(n)}(s)\, ds \right) \\
& - \frac{1}{n} P_{-\boldsymbol{e}_{d+1}} \left(n \int_0^t \delta^{(n)} \left(\bar{N}^{(n)}(s) - \sum_{i=1}^d \bar{I}_i^{(n)}(s) - \bar{S}^{(n)}(s) \right) ds \right) \\
& - \sum_{i=1}^d \frac{1}{n} P_{-\boldsymbol{e}_i-\boldsymbol{e}_{d+1}} \left(n \int_0^t (\delta^{(n)} + \alpha_i^{(n)}) \bar{I}_i^{(n)}(s)\, ds \right).
\end{aligned}
$$

This formalism is useful because it will allow us to write each r.h.s. as the sum of a deterministic trend and of a stochastic term with zero expectation.

Recall that the marginal value $P(t)$ of a simple Poisson process at time t is a Poisson random variable with parameter t. In particular, $P(t) - t$ has mean 0 and variance t. So if we write

$$\tilde{P}(t) := P(t) - t,$$

we are writing $P(t)$ as the sum of a deterministic trend t and of a stochastic term $\tilde{P}(t)$ with mean 0. If we come back to the example of the Markov process X jumping at rate $f(X)$, we can write

$$X(t) = x_0 + \int_0^t f(X(s-))\, ds + M(t),$$

where we have set

$$M(t) := \tilde{P}\left(\int_0^t f(X(s-))\, ds \right).$$

In addition, since the increments $\tilde{P}(t+s) - \tilde{P}(t)$ are independent of the past before t, have mean 0 and variance s, we can write the last equation in differential form

$$dX(t) = f(X(t-))\, dt + dM(t),$$

with $dM(t) = U(t) - f(X(t-))\, dt$, where $U(t)$ equals 1 iff P jumps at $\int_0^t f(X(s-))\, ds$ and equals 0 otherwise. In particular, conditional on $X(t-) = x$, $dM(t)$ has mean 0 and variance $f(x)\, dt$. Thus, we also recover the infinitesimal variation of X as the sum of an infinitesimal trend in the dynamics

and of a stochastic fluctuation term with zero expectation.

Now let us return to our initial process. We adopt the same notation as previously, for example $\tilde{P}_{-\boldsymbol{e}_0-\boldsymbol{e}_{d+1}}(t) = P_{-\boldsymbol{e}_0-\boldsymbol{e}_{d+1}}(t) - t$ and

$$M^{(n)}_{-\boldsymbol{e}_0-\boldsymbol{e}_{d+1}}(t) := \tilde{P}_{-\boldsymbol{e}_0-\boldsymbol{e}_{d+1}}\left(n\int_0^t \delta^{(n)}\bar{S}^{(n)}(s)\,ds\right).$$

Proposition 1. *The infinitesimal variation of $\bar{\boldsymbol{E}}^{(n)}(t)$ can be written as the sum of an infinitesimal deterministic trend and of a stochastic fluctuation term with zero expectation:*

$$d\bar{S}^{(n)}(t) = F_0^{(n)}\left(\bar{\boldsymbol{E}}^{(n)}(t)\right)dt + \frac{1}{n}dM^{(n)}_{\mathbf{e}_0+\mathbf{e}_{d+1}}(t) - \frac{1}{n}dM^{(n)}_{-\mathbf{e}_0-\mathbf{e}_{d+1}}(t) - \frac{1}{n}\sum_{i=1}^{d} dM^{(n)}_{-\mathbf{e}_0+\mathbf{e}_i}(t)$$

$$d\bar{I}_i^{(n)}(t) = F_i^{(n)}\left(\bar{\boldsymbol{E}}^{(n)}(t)\right)dt + \frac{1}{n}dM^{(n)}_{-\mathbf{e}_0+\mathbf{e}_i}(t) - \frac{1}{n}dM^{(n)}_{-\mathbf{e}_i-\mathbf{e}_{d+1}}(t) - \frac{1}{n}dM^{(n)}_{-\mathbf{e}_i}(t)$$

$$d\bar{N}^{(n)}(t) = F_{d+1}^{(n)}\left(\bar{\boldsymbol{E}}^{(n)}(t)\right)dt + \frac{1}{n}dM^{(n)}_{\mathbf{e}_0+\mathbf{e}_{d+1}}(t) - \frac{1}{n}dM^{(n)}_{-\mathbf{e}_0-\mathbf{e}_{d+1}}(t) - \frac{1}{n}\sum_{i=1}^{d} dM^{(n)}_{-\mathbf{e}_i-\mathbf{e}_{d+1}}(t) - \frac{1}{n}dM^{(n)}_{-\mathbf{e}_{d+1}}(t),$$

where

$$F_0^{(n)}(\boldsymbol{x}) = \lambda^{(n)} - \left(\sum_{i=1}^{d}\beta_i^{(n)}\frac{x_i}{x_{d+1}} + \delta^{(n)}\right)x_0$$

$$F_i^{(n)}(\boldsymbol{x}) = \left(\beta_i^{(n)}\frac{x_0}{x_{d+1}} - (\delta^{(n)} + \alpha_i^{(n)} + \gamma_i^{(n)})\right)x_i$$

$$F_{d+1}^{(n)}(\boldsymbol{x}) = \lambda^{(n)} - \delta^{(n)}x_{d+1} - \sum_{i=1}^{d}\alpha_i^{(n)}x_i,$$

and $dM^{(n)}_{\mathbf{e}_0+\mathbf{e}_{d+1}}(t)$, $dM^{(n)}_{-\mathbf{e}_0-\mathbf{e}_{d+1}}(t)$, $dM^{(n)}_{-\mathbf{e}_0+\mathbf{e}_i}(t)$, $dM^{(n)}_{-\mathbf{e}_i-\mathbf{e}_{d+1}}(t)$, $dM^{(n)}_{-\mathbf{e}_i}(t)$ and $dM^{(n)}_{-\mathbf{e}_{d+1}}(t)$, are independent infinitesimal noise terms with mean zero and respective infinitesimal variances $n\rho^{(n)}_{\mathbf{e}_0+\mathbf{e}_{d+1}}\left(\bar{\boldsymbol{E}}^{(n)}(t)\right)dt$, $n\rho^{(n)}_{-\mathbf{e}_0-\mathbf{e}_{d+1}}\left(\bar{\boldsymbol{E}}^{(n)}(t)\right)dt$, $n\rho^{(n)}_{-\mathbf{e}_0+\mathbf{e}_i}\left(\bar{\boldsymbol{E}}^{(n)}(t)\right)dt$, $n\rho^{(n)}_{-\mathbf{e}_i-\mathbf{e}_{d+1}}\left(\bar{\boldsymbol{E}}^{(n)}(t)\right)dt$, $n\rho^{(n)}_{-\mathbf{e}_i}\left(\bar{\boldsymbol{E}}^{(n)}(t)\right)dt$ and $n\rho^{(n)}_{-\mathbf{e}_{d+1}}\left(\bar{\boldsymbol{E}}^{(n)}(t)\right)dt$, where

$$\rho^{(n)}_{\mathbf{e}_0+\mathbf{e}_{d+1}}(\boldsymbol{x}) = \lambda^{(n)}$$

$$\rho^{(n)}_{-\mathbf{e}_0-\mathbf{e}_{d+1}}(\boldsymbol{x}) = \delta^{(n)}x_0$$

$$\rho^{(n)}_{-\mathbf{e}_0+\mathbf{e}_i}(\boldsymbol{x}) = \beta_i^{(n)}\frac{x_0\,x_i}{x_{d+1}}$$

$$\rho^{(n)}_{-\mathbf{e}_i-\mathbf{e}_{d+1}}(\boldsymbol{x}) = (\delta^{(n)} + \alpha_i^{(n)})x_i$$

$$\rho^{(n)}_{-\mathbf{e}_i}(\boldsymbol{x}) = \gamma_i^{(n)}x_i$$

$$\rho^{(n)}_{-\mathbf{e}_{d+1}}(\boldsymbol{x}) = \delta^{(n)}\left(x_{d+1} - \sum_{i=0}^{d}x_i\right).$$

Remark 2. Note that $n\rho_{\boldsymbol{l}}^{(n)}\left(\bar{\boldsymbol{E}}^{(n)}(t)\right)$, is rate at which the Markov process $(\boldsymbol{E}^{(n)}(t))$ makes a jump $\boldsymbol{l}$.

Setting

$$\boldsymbol{M}^{(n)}(t) := (\boldsymbol{e}_0 + \boldsymbol{e}_{d+1})M^{(n)}_{\boldsymbol{e}_0+\boldsymbol{e}_{d+1}}(t) - (\boldsymbol{e}_0 + \boldsymbol{e}_{d+1})M^{(n)}_{-\boldsymbol{e}_0-\boldsymbol{e}_{d+1}}(t) + \sum_{i=1}^{d}(\boldsymbol{e}_i - \boldsymbol{e}_0)M^{(n)}_{-\boldsymbol{e}_0+\boldsymbol{e}_i}(t) - \sum_{i=1}^{d}(\boldsymbol{e}_i + \boldsymbol{e}_{d+1})M^{(n)}_{-\boldsymbol{e}_i-\boldsymbol{e}_{d+1}}(t) - \sum_{i=1}^{d}\boldsymbol{e}_i M^{(n)}_{-\boldsymbol{e}_i}(t) - \boldsymbol{e}_{d+1}M^{(n)}_{-\boldsymbol{e}_{d+1}}(t), \quad \text{(S.2)}$$

the result in the proposition can be written more compactly as

$$d\bar{\boldsymbol{E}}^{(n)}(t) = \boldsymbol{F}^{(n)}\left(\bar{\boldsymbol{E}}^{(n)}(t)\right)\,dt + \frac{1}{n}d\boldsymbol{M}^{(n)}(t), \quad \text{(S.3)}$$

where

$$\boldsymbol{F}^{(n)}(\boldsymbol{x}) = (F_0^{(n)}(\boldsymbol{x}), F_1^{(n)}(\boldsymbol{x}), \dots F_d^{(n)}(\boldsymbol{x}), F_{d+1}^{(n)}(\boldsymbol{x})).$$

Thus, the function $\boldsymbol{F}^{(n)}(\boldsymbol{x})$ describes the infinitesimal trend in the dynamics, whereas the terms $M_{\boldsymbol{l}}^{(n)}(t)$ capture the de-trended fluctuations corresponding to each type of possible event. This equation is analogous to an Itô SDE, only now the driving noise is the discontinuous $\boldsymbol{M}^{(n)}(t)$, rather than the more familiar Brownian motion.

We note that for $i = 1, \dots, d$,

$$M_i^{(n)}(t) = M^{(n)}_{-\boldsymbol{e}_0+\boldsymbol{e}_i}(t) - M^{(n)}_{-\boldsymbol{e}_i-\boldsymbol{e}_{d+1}}(t) - M^{(n)}_{-\boldsymbol{e}_i}(t),$$

so that $M_i^{(n)}(t)$ is independent of $M_j^{(n)}(t)$ for all $1 \le i \ne j \le d$, whereas

$$\mathbb{E}\left[dM_i^{(n)}(t)^2\right] = \left(\beta_i^{(n)}\frac{S^{(n)}(t)}{N^{(n)}(t)} + (\delta^{(n)} + \alpha_i^{(n)} + \gamma_i^{(n)})\right)I_i^{(n)}(t), \quad \text{(S.4)}$$

two facts that will prove useful in what follows.

In particular, it makes sense to define $\boldsymbol{a}^{(n)}$ as the infinitesimal variance-covariance matrix of $\bar{\boldsymbol{E}}^{(n)}(t)$ by

$$a_{ij}^{(n)}\left(\bar{\boldsymbol{E}}^{(n)}(t)\right)\,dt := \operatorname{Cov}\left[d\bar{E}_i^{(n)}(t), d\bar{E}_j^{(n)}(t)\right] = \frac{1}{n^2}\operatorname{Cov}\left[dM_i^{(n)}(t), dM_j^{(n)}(t)\right]. \quad \text{(S.5)}$$

Let us compute $\boldsymbol{a}^{(n)}$. Because all distinct terms in the definition (S.2) of $M^{(n)}$ are independent, all cross terms vanish. For example, for any $1 \le i \le d$

$$\begin{aligned}
&\operatorname{Cov}\left[d\bar{E}_0^{(n)}(t), d\bar{E}_i^{(n)}(t)\right] \\
&= \frac{1}{n^2}\operatorname{Cov}\Bigg[dM^{(n)}_{\boldsymbol{e}_0+\boldsymbol{e}_{d+1}}(t) - dM^{(n)}_{-\boldsymbol{e}_0-\boldsymbol{e}_{d+1}}(t) - \sum_{j=1}^{d} dM^{(n)}_{-\boldsymbol{e}_0+\boldsymbol{e}_j}(t), \\
&\qquad dM^{(n)}_{-\boldsymbol{e}_0+\boldsymbol{e}_i}(t) - dM^{(n)}_{-\boldsymbol{e}_i-\boldsymbol{e}_{d+1}}(t) - dM^{(n)}_{-\boldsymbol{e}_i}(t)\Bigg]
\end{aligned}$$

$$= \frac{1}{n^2}\mathrm{Cov}\left[-dM^{(n)}_{-\boldsymbol{e}_0+\boldsymbol{e}_i}(t), dM^{(n)}_{-\boldsymbol{e}_0+\boldsymbol{e}_i}(t)\right]$$
$$= -\frac{1}{n^2}\mathrm{Var}\left[dM^{(n)}_{-\boldsymbol{e}_0+\boldsymbol{e}_i}(t)\right] = -\frac{1}{n}\rho^{(n)}_{-\boldsymbol{e}_0+\boldsymbol{e}_i}(\bar{\boldsymbol{E}}^{(n)}(t))\, dt,$$

which shows that $a^{(n)}_{0i}(\boldsymbol{x}) = -\frac{1}{n}\rho^{(n)}_{-\boldsymbol{e}_0+\boldsymbol{e}_i}(\boldsymbol{x})$. We should not be surprised by the fact that this infinitesimal covariance is negative. Each event where a susceptible is infected by strain i has the effect of simultaneously decreasing the number of susceptibles and increasing the number of individuals infected by strain i.

By similar calculations, it is easy to see that

$$a^{(n)}_{0,d+1}(\boldsymbol{x}) = \frac{1}{n}\rho^{(n)}_{\boldsymbol{e}_0+\boldsymbol{e}_{d+1}}(\boldsymbol{x}) + \frac{1}{n}\rho^{(n)}_{-\boldsymbol{e}_0-\boldsymbol{e}_{d+1}}(\boldsymbol{x}),$$

$$a^{(n)}_{d+1,d+1}(\boldsymbol{x}) = \frac{1}{n}\rho^{(n)}_{\boldsymbol{e}_0+\boldsymbol{e}_{d+1}}(\boldsymbol{x}) + \frac{1}{n}\rho^{(n)}_{-\boldsymbol{e}_0-\boldsymbol{e}_{d+1}}(\boldsymbol{x}) + \frac{1}{n}\sum_{i=1}^{d}\rho^{(n)}_{-\boldsymbol{e}_i-\boldsymbol{e}_{d+1}}(\boldsymbol{x}) + \frac{1}{n}\rho^{(n)}_{-\boldsymbol{e}_{d+1}}(\boldsymbol{x}),$$

and for any $1 \le i \le d$,

$$a^{(n)}_{i,d+1}(\boldsymbol{x}) = \frac{1}{n}\rho^{(n)}_{-\boldsymbol{e}_i-\boldsymbol{e}_{d+1}}(\boldsymbol{x}).$$

Also for any $1 \le i \ne j \le d$, $a^{(n)}_{ij}(\boldsymbol{x}) = 0$ and

$$a^{(n)}_{jj}(\boldsymbol{x}) = \frac{1}{n}\rho^{(n)}_{-\boldsymbol{e}_0+\boldsymbol{e}_j}(\boldsymbol{x}) + \frac{1}{n}\rho^{(n)}_{-\boldsymbol{e}_j-\boldsymbol{e}_{d+1}}(\boldsymbol{x}) + \frac{1}{n}\rho^{(n)}_{-\boldsymbol{e}_j}(\boldsymbol{x}).$$

In other words, for any $0 \le i, j \le d+1$,

$$a^{(n)}_{ij}(\boldsymbol{x}) = \begin{cases} \frac{1}{n}\left(\frac{\beta^{(n)}_i x_0 x_j}{x_{d+1}} + (\delta^{(n)} + \alpha^{(n)}_j + \gamma^{(n)}_j)x_j\right) & \text{if } i = j, \\ 0 & \text{otherwise.} \end{cases} \tag{S.6}$$

Equivalently,

$$\boldsymbol{a}^{(n)}(\boldsymbol{x}) = \frac{1}{n}\sum_{\boldsymbol{l}} \boldsymbol{l}^\top \boldsymbol{l}\rho_{\boldsymbol{l}}(\boldsymbol{x}), \tag{S.7}$$

where the sum is over all possible jumps $\boldsymbol{l}$ of the Markov process $(\boldsymbol{E}^{(n)}(t))$.

3.1 Obtaining Equation (4)

Note that similarly to Brownian motion, the infinitesimal mean of $\frac{1}{\sqrt{n}}M^{(n)}_{\boldsymbol{l}}(t)$ during the time interval dt is zero and its infinitesimal variance is $\rho^{(n)}_{\boldsymbol{l}}\left(\tilde{\boldsymbol{E}}^{(n)}(t)\right)dt.$, whereas the jump size $\frac{1}{\sqrt{n}}$ tends to 0 as $n \to \infty$ (and thus, $\frac{1}{\sqrt{n}}M^{(n)}_{\boldsymbol{l}}(t)$ is approximately continuous for large n), we see that for large values of n, this noise is approximately equal to a Brownian motion with the same variance:

$$\frac{1}{\sqrt{n}}M^{(n)}_{\boldsymbol{l}}(t) \approx \sqrt{\rho^{(n)}_{\boldsymbol{l}}\left(\tilde{\boldsymbol{E}}^{(n)}(t)\right)}\, dB_{\boldsymbol{l}}(t),$$

where all Brownian motions $B_{\boldsymbol{e}_0+\boldsymbol{e}_{d+1}}$, $B_{-\boldsymbol{e}_0-\boldsymbol{e}_{d+1}}$, $B_{-\boldsymbol{e}_0+\boldsymbol{e}_i}$, $B_{-\boldsymbol{e}_i-\boldsymbol{e}_{d+1}}$, $B_{-\boldsymbol{e}_i}$ and $B_{-\boldsymbol{e}_{d+1}}$ are independent.

This allows us to rewrite the results in Proposition 1 in the form of the following diffusion approximation for n large,

$$
\begin{aligned}
d\bar{S}^{(n)}(t) \approx F_0^{(n)}\left(\bar{\boldsymbol{E}}^{(n)}(t)\right)dt + \frac{1}{\sqrt{n}}\sqrt{\rho^{(n)}_{\boldsymbol{e}_0+\boldsymbol{e}_{d+1}}\left(\bar{\boldsymbol{E}}^{(n)}(t)\right)}\,dB_{\boldsymbol{e}_0+\boldsymbol{e}_{d+1}}(t) \\
-\frac{1}{\sqrt{n}}\sqrt{\rho^{(n)}_{-\boldsymbol{e}_0-\boldsymbol{e}_{d+1}}\left(\bar{\boldsymbol{E}}^{(n)}(t)\right)}\,dB_{-\boldsymbol{e}_0-\boldsymbol{e}_{d+1}}(t) - \frac{1}{\sqrt{n}}\sum_{i=1}^{d}\sqrt{\rho^{(n)}_{-\boldsymbol{e}_0+\boldsymbol{e}_i}\left(\bar{\boldsymbol{E}}^{(n)}(t)\right)}\,dB_{-\boldsymbol{e}_0+\boldsymbol{e}_i}(t)
\end{aligned}
$$

$$
\begin{aligned}
d\bar{I}_i^{(n)}(t) \approx F_i^{(n)}\left(\bar{\boldsymbol{E}}^{(n)}(t)\right)dt + \frac{1}{\sqrt{n}}\sqrt{\rho^{(n)}_{-\boldsymbol{e}_0+\boldsymbol{e}_i}\left(\bar{\boldsymbol{E}}^{(n)}(t)\right)}dB_{-\boldsymbol{e}_0+\boldsymbol{e}_i}(t) \\
-\frac{1}{\sqrt{n}}\sqrt{\rho^{(n)}_{-\boldsymbol{e}_i-\boldsymbol{e}_{d+1}}\left(\bar{\boldsymbol{E}}^{(n)}(t)\right)}dB_{-\boldsymbol{e}_i-\boldsymbol{e}_{d+1}}(t) - \frac{1}{\sqrt{n}}\sqrt{\rho^{(n)}_{-\boldsymbol{e}_i}\left(\bar{\boldsymbol{E}}^{(n)}(t)\right)}dB_{-\boldsymbol{e}_i}(t)
\end{aligned}
$$

$$
\begin{aligned}
d\bar{N}^{(n)}(t) \approx F_{d+1}^{(n)}\left(\bar{\boldsymbol{E}}^{(n)}(t)\right)dt + \frac{1}{\sqrt{n}}\sqrt{\rho^{(n)}_{\boldsymbol{e}_0+\boldsymbol{e}_{d+1}}\left(\bar{\boldsymbol{E}}^{(n)}(t)\right)}dB_{\boldsymbol{e}_0+\boldsymbol{e}_{d+1}}(t) \\
-\frac{1}{\sqrt{n}}\sqrt{\rho^{(n)}_{-\boldsymbol{e}_0-\boldsymbol{e}_{d+1}}\left(\bar{\boldsymbol{E}}^{(n)}(t)\right)}dB_{-\boldsymbol{e}_0-\boldsymbol{e}_{d+1}}(t) - \frac{1}{\sqrt{n}}\sum_{i=1}^{d}\sqrt{\rho^{(n)}_{-\boldsymbol{e}_i-\boldsymbol{e}_{d+1}}\left(\bar{\boldsymbol{E}}^{(n)}(t)\right)}dB_{-\boldsymbol{e}_i-\boldsymbol{e}_{d+1}}(t) \\
-\frac{1}{\sqrt{n}}\sqrt{\rho^{(n)}_{-\boldsymbol{e}_{d+1}}\left(\bar{\boldsymbol{E}}^{(n)}(t)\right)}dB_{-\boldsymbol{e}_{d+1}}(t)
\end{aligned}
$$

(see [23] for a rigorous statement).

Setting

$$\bar{I}^{(n)}(t) := \sum_{l=1}^{d}\bar{I}_l^{(n)}(t),$$

so that

$$d\bar{I}^{(n)}(t) = \sum_{l=1}^{d}d\bar{I}_l^{(n)}(t),$$

and combining independent Brownian motions, we obtain equation (3) in the main text (*n.b.,* to simplify notation in the main text, we use S, I and R in lieu of $\bar{S}^{(n)}(t)$, $\bar{I}^{(n)}(t)$ and $\bar{N}^{(n)}(t)$).

We shall not use this diffusion approximation in the sequel, where we continue to consider the process with discrete jumps, (S.3).

4 Itô's Formula and Derivation of Equation (5)

As a first application of the SDE representation, we apply Itô's formula with jumps to our process to obtain an SDE for the proportion of each strain.

To motivate this, suppose we had a deterministic differential equation

$$\dot{\boldsymbol{Y}}(t) = \boldsymbol{f}(\boldsymbol{Y}(t))$$

and we let $X(t)$ be a deterministic real function of $\boldsymbol{Y}(t)$, say

$$X(t) := g(\boldsymbol{Y}(t))$$

where $g : \mathbb{R}^{d+2} \to \mathbb{R}$ is assumed to be continuously differentiable.

Then, applying the chain rule, we derive a differential equation satisfied by $X(t)$:

$$\dot{X}(t) = \sum_{j=0}^{d+1} \frac{\partial g}{\partial x_j}(\boldsymbol{Y}(t)) f_j(\boldsymbol{Y}(t))$$

or equivalently

$$X(t) = X(0) + \int_0^t \sum_{j=0}^{d+1} \frac{\partial g}{\partial x_j}(\boldsymbol{Y}(s)) f_j(\boldsymbol{Y}(s))\, ds$$

The analogue of the chain rule in the fully stochastic case is the Meyer-Itô's formula (see *e.g.,* [29]).

$$\begin{aligned} X^{(n)}(t) = X^{(n)}(0) &+ \int_0^t \sum_{j=0}^{d+1} \frac{\partial g}{\partial x_j}(\boldsymbol{E}^{(n)}(s)) F_j^{(n)}(\bar{\boldsymbol{E}}^{(n)}(s)) \\ &+ \frac{1}{2} \sum_{j,k=0}^{d+1} a_{jk}^{(n)}(\bar{\boldsymbol{E}}^{(n)}(s)) \frac{\partial^2 g}{\partial x_j \partial x_k}(\bar{\boldsymbol{E}}^{(n)}(s))\, ds + \frac{1}{n}\int_0^t \sum_{j=0}^{d+1} \frac{\partial g}{\partial x_j}(\boldsymbol{E}^{(n)}(s))\, dM_j^{(n)}(s) + \varepsilon^{(n)}(t), \end{aligned} \quad \text{(S.8)}$$

where $\boldsymbol{a}^{(n)}(\boldsymbol{x})$ is the infinitesimal variance-covariance matrix of $\bar{\boldsymbol{E}}^{(n)}(t)$ defined in (S.6) and

$$\begin{aligned} \varepsilon^{(n)}(t) = \sum_{s<t} g(\bar{\boldsymbol{E}}^{(n)}(s)) - g(\bar{\boldsymbol{E}}^{(n)}(s-)) &- \sum_{j=0}^{d+1} \frac{\partial g}{\partial x_j}(\bar{\boldsymbol{E}}^{(n)}(s-)) \Delta \bar{E}_j^{(n)}(s) \\ &- \frac{1}{2} \sum_{j,k=0}^{d+1} \frac{\partial^2 g}{\partial x_j \partial x_k}(\bar{\boldsymbol{E}}^{(n)}(s-)) \Delta \bar{E}_j^{(n)}(s) \Delta \bar{E}_k^{(n)}(s), \end{aligned} \quad \text{(S.9)}$$

where the sum is over the times s of discontinuity of $\bar{\boldsymbol{E}}^{(n)}$. At a time t of discontinuity,

$$\Delta \bar{E}_j^{(n)}(t) := \bar{E}_j^{(n)}(t) - \bar{E}_j^{(n)}(t-)$$

denotes the magnitude of the jump in $\bar{E}_j^{(n)}$ at time t. The term $\varepsilon^{(n)}(t)$ correcting for discontinuities distinguishes the more general Meyer-Itô formula from the familiar Itô's formula for diffusions. In Section 8.1, we show that $\varepsilon^{(n)}(t) = \mathcal{O}\big(1/n^2\big)$.

Using this, we can derive Equation (5) from the main text. Let

$$\Pi_i(\boldsymbol{x}) = \frac{x_i}{\sum_{l=1}^d x_l}$$

so that

$$P_i^{(n)}(t) = \Pi_i(\bar{\boldsymbol{I}}^{(n)}(t)) = \Pi_i(\bar{\boldsymbol{E}}^{(n)}(t)).$$

is the proportion of the population infected with strain i. Since $P_i^{(n)}(t)$ is a deterministic function of $\bar{\boldsymbol{E}}^{(n)}(t)$, we can use Itô's formula (S.8) for jump processes. The following statement will be proved rigorously in Section 8.1.

Proposition 2. *The fraction of the population infected by strain i satisfies*

$$\begin{aligned} P_i^{(n)}(t) = P_i^{(n)}(0) + \int_0^t P_i^{(n)}(s) \left(r_i^{(n)}(\bar{S}^{(n)}(s), \bar{N}^{(n)}(s)) - \sum_{j=1}^d r_j^{(n)}(\bar{S}^{(n)}(s), \bar{N}^{(n)}(s)) P_j^{(n)}(s) \right) \\ + \frac{1}{n} \frac{1}{\sum_{l=1}^d \bar{I}_l^{(n)}(s)} P_i^{(n)}(s) \left(v_i^{(n)}(\bar{S}^{(n)}(s), \bar{N}^{(n)}(s)) - \sum_{j=1}^d v_j^{(n)}(\bar{S}^{(n)}(s), \bar{N}^{(n)}(s)) P_j^{(n)}(s) \right) ds \\ + \frac{1}{n} \int_0^t \frac{1}{\sum_{l=1}^d \bar{I}_l^{(n)}(s)} \sum_{j=1}^d \left(\mathbb{1}_{\{i=j\}} - P_i^{(n)}(s) \right) dM_j^{(n)}(s) + \varepsilon_i^{(n)}(t), \end{aligned} \tag{S.10}$$

where

$$r_i^{(n)}(x_0, x_{d+1}) := \beta_i^{(n)} \frac{x_0}{x_{d+1}} - (\delta^{(n)} + \alpha_i^{(n)} + \gamma_i^{(n)})$$

gives the Malthusian growth rate of strain i, whereas

$$v_i^{(n)}(x_0, x_{d+1}) := \beta_i^{(n)} \frac{x_0}{x_{d+1}} + (\delta^{(n)} + \alpha_i^{(n)} + \gamma_i^{(n)})$$

is the infinitesimal variance associated with the growth of strain i. In addition, for any $T > 0$*, there is a constant C such that for all* $t \in [0, T]$, $\mathbb{P}\{|n^2 \varepsilon_i^{(n)}(t)| \geq C\}$ *vanishes as* $n \to \infty$.

To obtain Equation (5) in the main text, we omit the lower order error term $\varepsilon_i^{(n)}(t) = \mathcal{O}(1/n^2)$, recall that

$$I^{(n)}(t) = \sum_{l=1}^d I_l^{(n)}(t) = n \sum_{l=1}^d \bar{I}_l^{(n)}(t)$$

gives the total number of infectives, and observe that, similarly to the previous section,

$$\frac{1}{n} dM_i^{(n)} \approx \frac{1}{\sqrt{n}} \sqrt{v_i^{(n)}(\bar{S}^{(n)}(s), \bar{N}^{(n)}(s)) \bar{I}_i^{(n)}(t)}\, dB_i(t)$$

for independent Brownian motions $B_1, \ldots, B_d$, so that

$$\frac{1}{n} \frac{1}{\sum_{l=1}^d \bar{I}_l^{(n)}(s)} dM_j^{(n)} \approx \frac{1}{\sqrt{I^{(n)}(t)}} \sqrt{v_i^{(n)}(\bar{S}^{(n)}(s), \bar{N}^{(n)}(s)) P_i^{(n)}(t)}\, dB_i(t).$$

5 Probability of Fixation of a Mutant Pathogen

In this section, we will be interested in the long time behaviour of our multi-strain stochastic epidemics. In particular, we tackle the problem of predicting which strains will be outcompeted and which strains will fix.

5.1 A Deterministic Limit and its Asymptotic Analysis

We begin this section with a result stating the convergence to a deterministic dynamical system as $n \to \infty$.

Proposition 3 (Theorem 2.2, [23]). *If*

$$\bar{S}^{(n)}(0) \to S(0), \quad \bar{I}_i^{(n)}(0) \to I_i(0), \quad \textit{and} \quad \bar{N}^{(n)}(0) \to N(0)$$

as $n \to \infty$, then for any fixed $T > 0$, with probability 1,

$$\sup_{t \le T} \|\bar{\boldsymbol{E}}^{(n)}(t) - \boldsymbol{E}(t)\| \to 0, \tag{S.11}$$

where $\boldsymbol{E}(t) := (S(t), I_1(t), \ldots, I_d(t), N(t))$ is the solution to the following system of ordinary differential equations:

$$\dot{S}(t) = \lambda - \left(\sum_{i=1}^{d} \beta_i \frac{I_i(t)}{N(t)} + \delta\right) S(t), \tag{S.12a}$$

$$\dot{I}_i(t) = \left(\beta_i \frac{S(t)}{N(t)} - (\delta + \alpha_i + \gamma_i)\right) I_i(t), \tag{S.12b}$$

$$\dot{N}(t) = \lambda - \delta N(t) - \sum_{i=1}^{d} \alpha_i I_i(t), \tag{S.12c}$$

with initial conditions $S(0)$, $\boldsymbol{I}(0)$, and $N(0)$.

Note that the result in the previous proposition can be written more compactly as

$$\dot{\boldsymbol{E}} = \boldsymbol{F}(\boldsymbol{E}),$$

where

$$\boldsymbol{F}(\boldsymbol{x}) = \lim_{n \to \infty} \boldsymbol{F}^{(n)}(\boldsymbol{x}).$$

While we continue to work with the finite n fully stochastic process, the bifurcation structure of the deterministic system (S.12) will guide our analysis of the stochastic model. In particular, the steady states of this model, together with the degenerate case that arises when stability is exchanged between fixed points, give rise to two regimes that correspond to strong and weak selection in classical population genetics. To be explicit, let

$$R_{0,i} := \frac{\beta_i}{\delta + \alpha_i + \gamma_i}.$$

be the basic reproduction number of strain i. $R_{0,i}$ is the expected total number of new infections caused by a single infected individual, assuming an unlimited supply of susceptibles.

If $R_{0,i} \neq R_{0,j}$ for all $1 \leq i \neq j \leq d$, the equations (S.12) have $d+1$ fixed points, one at $\mathbf{0}$ and one at the d equilibria where the population is infected by a single strain

$$\boldsymbol{E}^{\star,i} := (S^{\star,i}, I_i^{\star,i}, \dots, I_d^{\star,i}, N^{\star,i}),$$

where

$$S^{\star,i} := \frac{\lambda}{\delta R_{0,i}} \left(1 - \frac{\alpha_1(R_{0,i}-1)}{\beta_1 - \alpha_1}\right), \quad I_j^{\star,i} := \begin{cases} \frac{\lambda(R_{0,i}-1)}{\beta_i - \alpha_i} & \text{if } i = j, \text{ and} \\ 0 & \text{otherwise,} \end{cases} \quad \text{and} \quad N^{\star,i} := R_{0,i} S^{\star}. \tag{S.13}$$

When $d = 1$, it is shown in [32] when $\delta > \alpha_1$ that: if $R_{0,1} > 1$ then unique endemic equilibrium of the strain, $\bar{\boldsymbol{E}}^{\star,1}$ is globally asymptotically stable, whereas if $R_{0,i} \leq 1$, the disease-free equilibrium $\mathbf{0}$ is globally asymptotically stable. The stability of fixed points is slightly more subtle when there is more than one strain.

Definition 1. We distinguish between two regimes of selection.

(i) The *strong selection* case, when $R_{0,1} > R_{0,i}$ for all $i > 1$, $\delta > \alpha_1$ and $R_{0,1} > 1$;

(ii) The *weak selection* case, $R_{0,1} = R_{0,i} = R_0^\star$ for $i \leq m$, whilst $R_0^\star > R_{0,j}$ for $m <\leq d$.

Proposition 4. *The long term behavior of the deterministic system* (S.12) *differs according to the selection regime.*

(i) In the strong selection *case, the equilibrium state $\bar{\boldsymbol{E}}^{\star,1}$ with strain 1 endemic and all other strains extinct is globally asymptotically stable from any initial condition for which $I_1(0) > 0$.*

(ii) In the weak selection *case, we arrive at a degenerate situation in which deterministic coexistence of strains $1, \dots, d$ is possible. Strains $m+1, \dots, d$ will eventually disappear, whereas all points $\boldsymbol{x} \in \mathbb{R}_+^{d+2}$ such that*

$$\begin{aligned} \sum_{i=1}^{m} (\beta_i - \alpha_i) x_i &= \lambda(R_0^\star - 1), \\ x_{m+1} = \cdots = x_d &= 0, \\ x_{d+1} &= \frac{1}{\delta}\left(\lambda - \sum_{i=1}^{m} \alpha_i x_i\right), \\ x_{d+1} &= R_0^\star x_0 \end{aligned} \tag{S.14}$$

are fixed points for the system (S.12)*. The set Ω of such points is globally attracting, but no point in Ω is an attracting fixed point.*

Proof. Point (i) follows by a direct adaptation of the result in [6]: rearranging (S.12b), we see that

$$\frac{1}{\beta_i}\frac{\dot{I}_i(t)}{I_i(t)} + \frac{1}{R_{0,i}} = \frac{S(t)}{N(t)} = \frac{1}{\beta_1}\frac{\dot{I}_1(t)}{I_1(t)} + \frac{1}{R_{0,1}},$$

so that

$$\left(\frac{I_i(t)}{I_i(0)}\right)^{\frac{1}{\beta_i}} e^{\frac{1}{R_{0,i}}t} = \left(\frac{I_1(t)}{I_1(0)}\right)^{\frac{1}{\beta_1}} e^{\frac{1}{R_{0,1}}t},$$

and, recalling that $I_1(t)$ is bounded for all $t > 0$, we see that for all $i > 1$

$$I_i(t) = I_i(0) \left(\frac{I_1(t)}{I_1(0)}\right)^{\frac{\beta_i}{\beta_1}} e^{-\beta_i \frac{R_{0,1}-R_{0,i}}{R_{0,1}R_{0,i}}t} \to 0$$

as $t \to \infty$.

The same argument shows that in the weak selection case, strains $m+1, \ldots, d$ will eventually disappear, whereas all points in Ω are fixed points. Moreover, all vectors $\boldsymbol{u}$ tangent to Ω, *i.e.,* such that

$$\sum_{i=1}^{m} (\beta_i - \alpha_i) u_i = 0, \quad u_{m+1} = \cdots = u_d = 0, \quad \text{and} \quad u_{d+1} = R_0^\star u_0,$$

are all eigenvectors to the Jacobian of $\boldsymbol{F}$ – evaluated at any $\boldsymbol{x} \in \Omega$ – corresponding to the eigenvalue 0. Thus, while Ω is a globally attracting set, no point in Ω is an attracting fixed point. □

We now turn to the computation of the fixation probability of a novel strain in the fully stochastic system. Informed by the previous statement, we will consider two cases, strong and weak selection, where the dynamics of the process – and thus our approach to the fixation probabilities – are qualitatively different. We will then show, despite the difference in the approaches, and in the expressions for the fixation probability thereby obtained, that our two results for the fixation probability agree on all intermediate scalings, and may thus be combined (heuristically) via the method of matched asymptotic expansions, to obtain a single expression valid across all scales.

5.2 The Strong Selection Case

We begin by recalling that for the deterministic approximation (S.12) to apply, we required that $\bar{S}^{(n)}(0) \to S(0)$, $\bar{I}_i^{(n)}(0) \to I_i(0)$ and $\bar{N}^{(n)}(0) \to N(0)$ as $n \to \infty$. Unpacking this assumption, we see that

$$I_i^{(n)}(0) = nI_i(0) + o(n),$$

i.e., that a non-trivial portion of the population is already infected with strain i.

For any strain with $I_i^{(n)}(0) \ll n$, $\bar{I}_i^{(n)}(0) \to 0$, and thus $I_i(t) \equiv 0$ for all $t \leq T$, for any fixed $T > 0$: until $\mathcal{O}(n)$ individuals are infected, strain i is effectively invisible to the deterministic approximation on any finite time interval. This is not to say that the strain is absent, but rather, if we sample individuals from the population uniformly at random, the probability of sampling an individual infected with strain i is zero.

We will consider the case when a fixed number k of strain 2 individuals invade an established resident population. For our purposes, a strain i is established if it is initially present in macroscopic numbers, *i.e.,*

$$\bar{I}_i^{(n)}(0) \to I_i(0) > 0.$$

In light of the results in 5.1, we will assume that there is only a single resident strain, strain 1. We will first consider the case when the resident strain is in endemic equilibrium, and then generalise to the case when the resident strain, whilst still present in macroscopic numbers, is initially away from equilibrium.

Should the invading strain, strain 2, exceed εn individuals, for any $\varepsilon > 0$, we arrive again in the domain of applicability of the deterministic approximation ($\bar{I}_2^{(n)}(0) \to I_2(0) > \varepsilon > 0$). If the reproductive number of the invader is greater than that of the resident, $R_{0,2} > R_{0,1}$, then for n sufficiently large, the dynamics are essentially deterministic, and with high probability (*i.e.,* tending to 1 as $n \to \infty$) the process will in finite time T_ε enter an ε-neighbourhood of the fixed point $\boldsymbol{E}^{\star,2}$ for arbitrarily small $\varepsilon > 0$. Once the process reaches this new equilibrium, we will see the resident strain is no longer viable, and subsequently disappears.

If we start at the endemic equilibrium of the resident strain 1, $\boldsymbol{E}^{\star,1}$, then until $I_2^{(n)}$ exceeds εn, the epidemic process $\boldsymbol{E}^{(n)}(t)$ will remain close to that point. We thus have

$$\frac{S^{(n)}(t)}{N^{(n)}(t)} = \frac{\bar{S}^{(n)}(t)}{\bar{N}^{(n)}(t)} \approx \frac{1}{R_{0,1}},$$

and to first approximation, strain 2 has per-host transmission and clearance/mortality rates of

$$\frac{\beta_2}{R_{0,1}} \quad \text{and} \quad \delta + \alpha_2 + \gamma_2.$$

This latter is a birth and death process (see *e.g.,* [3]) which will go extinct with probability

$$q = \frac{\delta + \alpha_2 + \gamma_2}{\frac{\beta_2}{R_{0,1}}} = \frac{R_{0,1}}{R_{0,2}}$$

if $R_{0,2} > R_{0,1}$, and with probability $q = 1$ otherwise. This probability of extinction q is for a single initial individual infected with strain 2, and becomes q^k for k initial individuals. Now, a birth and death process either goes extinct or grows arbitrarily large, so with probability $1 - \frac{R_{0,2}}{R_{0,1}}$ it will eventually exceed εn.

Similarly, when we have reached a neighbourhood of $\boldsymbol{E}^{\star,2}$ the transmission and clearance/mortality rates of strain 1 are approximately

$$\frac{\beta_1}{R_{0,2}} \quad \text{and} \quad \delta + \alpha_1 + \gamma_1.$$

Since $R_{0,2} > R_{0,1}$, this is a subcritical birth-death process which goes extinct with probability 1. Thus, invasion implies replacement, where for our purposes, the process *invades* if it exceeds εn individuals for some fixed $\varepsilon > 0$.

To summarize, we have heuristically derived

Proposition 5 (Strong Selection). *Consider a population infected with 2 strains such that $R_{0,2} > R_{0,1}$.*

(i) [Macroscopic initial frequencies] *If $\bar{I}_2^{(n)}(0) \to I_2(0) > 0$, then strain 1 will go extinct with high probability.*

(ii) [Novel strain in small number of copies, resident at endemic equilibrium] *Suppose that $\bar{I}_1^{(n)}(0) \to \bar{I}^{\star,1}$, and that $I_2^{(n)}(0) = k$ for some fixed positive integer k. Then, provided $R_{0,2} > 1$, for any $\varepsilon > 0$*

$$\lim_{n\to\infty} \mathbb{P}\left\{I_1^{(n)}(t) = 0 \text{ and } \|\bar{\boldsymbol{E}}^{(n)}(t) - \bar{\boldsymbol{E}}^{\star,2}\| < \varepsilon \text{ for some } t < \infty\right\} = 1 - \left(\frac{R_{0,1}}{R_{0,2}}\right)^k. \quad \text{(S.15)}$$

In other words, the event that strain 1 becomes extinct asymptotically coincides with the event that strain 2 invades, which happens with a probability asymptotically equal to the probability of survival $1 - \left(\frac{R_{0,1}}{R_{0,2}}\right)^k$ of a time-homogeneous birth-death process; on this event, the system reaches in finite time the deterministic equilibrium (S.13) *with only strain 2 endemic.*

We give a rigorous proof of this result in Section 8.2, and compare (S.15) to fixation probabilities estimated from simulated epidemics in Figure S.4b.

5.3 The Weak Selection Case

Recall that weak selection corresponds to the case when $R_{0,i} = R_{0,j} = R_0^\star$ for $1 \le i, j \le m$, whereas $R_0^\star > R_{0,j}$ for $m < j \le d$ (to simplify the discussion, we consider only the case where $m = d$). We hasten to clarify, however, that

$$R_{0,i}^{(n)} = \frac{\beta_i^{(n)}}{\delta^{(n)} + \alpha_i^{(n)} + \gamma_i^{(n)}},$$

so our assumptions (S.1) only impose that

$$R_{0,i}^{(n)} = R_0^\star\left(1 + \frac{r_i}{n}\right) + o\left(\tfrac{1}{n}\right)$$

i.e., $R_{0,i}^{(n)}$ and $R_{0,j}^{(n)}$ are allowed to differ by $\mathcal{O}\left(\frac{1}{n}\right)$ terms; as we shall see below, this is analogous to the weak selection limit of classical population genetics, and the values r_i will appear as selection coefficients in a diffusion approximation.

In this case, we have a separation of timescales: there is a fast time-scale, in which (S.11) tells us that the stochastic process approximately follows the trajectories of (S.12) arbitrarily closely to an arbitrarily small neighbourhood of Ω. Then, as we discuss below, there is a slow-time scale, in which, having arrived at Ω, the stochastic process remains near this critical manifold.

Let

$$\hat{S}^{(n)}(t) := \bar{S}^{(n)}(nt) = \frac{1}{n} S^{(n)}(nt),$$
$$\hat{I}_i^{(n)}(t) := \bar{I}_i^{(n)}(nt) = \frac{1}{n} I_i^{(n)}(nt),$$
$$\hat{N}^{(n)}(t) := \bar{N}^{(n)}(nt) = \frac{1}{n} N^{(n)}(nt),$$

and let

$$\hat{\boldsymbol{E}}^{(n)}(t) := (\hat{S}^{(n)}(t), \hat{\boldsymbol{I}}_i^{(n)}(t), \hat{N}^{(n)}(t)),$$

where, as before, we let $\hat{\boldsymbol{I}}^{(n)}(t) = \left(\hat{I}_1^{(n)}(t), \ldots, \hat{I}_d^{(n)}(t)\right)$, and note that

$$(\hat{S}^{(n)}(0), \hat{\boldsymbol{I}}^{(n)}(0), \hat{N}^{(n)}(0)) = (\bar{S}^{(n)}(0), \bar{\boldsymbol{I}}^{(n)}(0), \bar{N}^{(n)}(0)).$$

Here, rescaling time by n is analogous to the passage to so-called "coalescent time" or "generation time", which is used to derive the diffusion limit of the Wright-Fisher model in classical population genetics.

Recalling (S.3), the SDE for $\hat{\boldsymbol{E}}^{(n)}(t)$ is then

$$\begin{aligned}
\hat{\boldsymbol{E}}^{(n)}(t) &= \hat{\boldsymbol{E}}^{(n)}(0) + \int_0^{nt} \boldsymbol{F}^{(n)}\left(\bar{\boldsymbol{E}}^{(n)}(s)\right) ds + \frac{1}{n}\boldsymbol{M}^{(n)}(nt) \\
&= \hat{\boldsymbol{E}}^{(n)}(0) + \int_0^{t} n\boldsymbol{F}^{(n)}\left(\hat{\boldsymbol{E}}^{(n)}(s)\right) ds + \frac{1}{n}\boldsymbol{M}^{(n)}(nt).
\end{aligned}$$

Thus, in the slow time scale, the advective component is accelerated by a factor of n, causing the process to move rapidly to the critical manifold Ω; as $n \to \infty$, this movement becomes instantaneous, and the process immediately jumps to Ω at time $t = 0$. Moreover, stochastic fluctuations away from Ω are restored instantaneously, so the process becomes "trapped" on Ω as $n \to \infty$. The following statement formalizes this idea using the projection $\boldsymbol{\pi}$ defined as follows. Let $\boldsymbol{E}(t, \boldsymbol{x}) = (S(t, \boldsymbol{x}), \boldsymbol{I}(t, \boldsymbol{x}), N(t, \boldsymbol{x}))$ be the solution to (S.12) with initial conditions $S(0) = x_0$, $I_i(0) = x_i$, and $N(0) = x_{d+1}$ and let

$$\boldsymbol{\pi}(\boldsymbol{x}) := \lim_{t\to\infty} \boldsymbol{E}(t, \boldsymbol{x}),$$

i.e., $\boldsymbol{\pi}(\boldsymbol{x})$ is the point on Ω at which the trajectory of (S.12) starting from $\boldsymbol{x}$ meets Ω.

Proposition 6. *As $n \to \infty$, $(\hat{\boldsymbol{E}}^{(n)}(t)) \Rightarrow (\hat{\boldsymbol{E}}(t))$*[1] *where the latter is a diffusion on the manifold Ω, solution to the system of stochastic differential equations*

$$d\hat{E}_i = \sum_{j=0}^{d+1} \frac{\partial \pi_i}{\partial x_j}(\hat{\boldsymbol{E}}) f_j(\hat{\boldsymbol{E}})\, dt + \frac{1}{2}\sum_{j=0}^{d+1}\sum_{k=0}^{d+1} \frac{\partial^2 \pi_i}{\partial x_j \partial x_k}(\hat{\boldsymbol{E}}) a_{jk}(\hat{\boldsymbol{E}})\, dt + \sum_{j=0}^{d+1}\sum_{k=1}^{D} \frac{\partial \pi_i}{\partial x_j}(\hat{\boldsymbol{E}}) \sigma_{jk}(\hat{\boldsymbol{E}})\, dB_k(t) \quad \text{(S.16)}$$

where the (B_k) denote $D = 3(d+1)$ independent standard Brownian motions,

$$\boldsymbol{f}(\boldsymbol{x}) := \lim_{n\to\infty} n\left(\boldsymbol{F}^{(n)}(\boldsymbol{x}) - \boldsymbol{F}(\boldsymbol{x})\right),$$

and $\boldsymbol{\sigma}(\boldsymbol{x})$ is the $(d+1)\times D$ matrix

$$\begin{bmatrix} \sqrt{\lambda} & -\sqrt{\delta x_0} & -\sqrt{\frac{\beta_1 x_0 x_1}{x_{d+1}}} & 0 & 0 & -\sqrt{\frac{\beta_2 x_0 x_2}{x_{d+1}}} & \cdots & 0 & -\sqrt{\frac{\beta_d x_0 x_d}{x_{d+1}}} & 0 & 0 & 0 \\ 0 & 0 & \sqrt{\frac{\beta_1 x_0 x_1}{x_{d+1}}} & -\sqrt{(\delta+\alpha_1)x_1} & -\sqrt{\gamma_1 x_1} & 0 & \cdots & \cdots & \cdots & \cdots & 0 & 0 \\ \vdots & \vdots & \ddots & \ddots & \ddots & \ddots & \ddots & \ddots & \ddots & \ddots & \ddots & \vdots \\ 0 & 0 & 0 & \cdots & \cdots & \cdots & \cdots & 0 & \sqrt{\frac{\beta_d x_0 x_d}{x_{d+1}}} & -\sqrt{(\delta+\alpha_d)x_d} & -\sqrt{\gamma_d x_d} & 0 \\ \sqrt{\lambda} & -\sqrt{\delta x_0} & 0 & -\sqrt{(\delta+\alpha_1)x_1} & 0 & 0 & \cdots & \cdots & 0 & -\sqrt{(\delta+\alpha_d)x_d} & 0 & -\sqrt{\delta\left(x_{d+1}-\sum_{i=0}^{d} x_i\right)} \end{bmatrix},$$

and

$$\boldsymbol{a}(\boldsymbol{x}) = \lim_{n\to\infty} n\boldsymbol{a}^{(n)}(\boldsymbol{x}) = \boldsymbol{\sigma}(\boldsymbol{x})\boldsymbol{\sigma}(\boldsymbol{x})^\top.$$

In other words, for each i

$$d\hat{\boldsymbol{E}}_i(t) = \boldsymbol{D}\pi_i(\hat{\boldsymbol{E}}(t))\boldsymbol{f}(\hat{\boldsymbol{E}}(t))\, dt + \boldsymbol{D}\pi_i(\hat{\boldsymbol{E}}(t))\,\boldsymbol{\sigma}(\hat{\boldsymbol{E}}(t))\, d\boldsymbol{B}(t) + \frac{1}{2}\mathrm{Tr}\left[\boldsymbol{\sigma}^\top(\hat{\boldsymbol{E}}(t))\boldsymbol{H}\pi_i(\hat{\boldsymbol{E}}(t))\,\boldsymbol{\sigma}(\hat{\boldsymbol{E}}(t))\right] dt, \quad \text{(S.17)}$$

where $D\pi_i$ denotes the gradient vector of π_i, $H\pi_i$ its Hessian matrix, and Tr *denotes the trace operator.*

This diffusion can be understood as the result of stochastic fluctuations around Ω immediately followed by a strong deterministic drift towards Ω.

As can be seen from Proposition 3, the drift pushes the process very rapidly onto Ω, so that in the limit, the process lives permanently in Ω. Now to understand the interplay between the deterministic dynamics towards Ω and the stochastic fluctuations around Ω, it is useful to think of the dynamics in two steps. Suppose that starting from a point $\hat{\boldsymbol{E}}(t-) \in \Omega$, the process $\boldsymbol{E}$ has a jump $\boldsymbol{l}$. Then, the rescaled process $(\hat{\boldsymbol{E}}^{(n)}(t))$ has a jump $\frac{1}{n}\boldsymbol{l}$.

[1] A family of random variables $\{X^{(n)}\}$ taking values in a space S is said to *converge weakly* to X if

$$\lim_{n\to\infty} \mathbb{E}[f(X^{(n)})] = \mathbb{E}[f(X)]$$

for all $f \in C(S)$; the values $\mathbb{E}[f(X)]$ completely characterise the distribution of X. Weak convergence is denoted by

$$X^{(n)} \Rightarrow X.$$

Here, S is the Skorokhod space $\mathbb{D}_{\mathbb{R}^{d+2}}[0,\infty)$ of right- continuous functions from $[0,\infty)$ to $\mathbb{R}^{d+2}$ with left limits; the interested reader is referred to [5] for a very readable account of weak convergence on $\mathbb{D}$.

In a second step, it is immediately projected back to the manifold by the drift *at the new location*, so:

$$d\hat{\boldsymbol{E}}(t) = \boldsymbol{\pi}\left(\hat{\boldsymbol{E}}(t-) + \frac{1}{n}\boldsymbol{l}\right) - \hat{\boldsymbol{E}}(t-)$$

Thus, expanding the i-th component of the r.h.s. of the last equation and recalling that $\boldsymbol{\pi}\left(\hat{\boldsymbol{E}}(t-)\right) = \hat{\boldsymbol{E}}(t-)$ yields

$$\begin{aligned} d\hat{E}_i^{(n)}(t) &= \pi_i\left(\hat{\boldsymbol{E}}^{(n)}(t-) + \frac{1}{n}\boldsymbol{l}\right) - E_i^{(n)}(t-) \\ &= \frac{1}{n}\sum_j \frac{\partial \pi_i}{\partial x_j}(\hat{\boldsymbol{E}}^{(n)}(t-))l_i + \frac{1}{n^2}\sum_j\sum_k \frac{1}{2}\frac{\partial^2\pi_i}{\partial x_j \partial x_k}(\hat{\boldsymbol{E}}^{(n)}(t-))l_j l_k + o\left(\tfrac{1}{n^2}\right). \end{aligned}$$

To determine $\mathbb{E}[d\hat{E}_i^{(n)}(t)]$ (*i.e.,* the ith component of the drift in the diffusion approximation) we need only sum this over all possible jumps $\boldsymbol{l}$, weighted by their probabilities:

$$\begin{aligned} &\mathbb{E}[d\hat{E}_i^{(n)}(t)] \\ &= \sum_{\boldsymbol{l}}\left(\frac{1}{n}\sum_j \frac{\partial \pi_i}{\partial x_j}(\boldsymbol{E}^{(n)}(t-))l_j + \frac{1}{n^2}\sum_j\sum_k \frac{1}{2}\frac{\partial^2\pi_i}{\partial x_j \partial x_k}(\boldsymbol{E}^{(n)}(t-))l_j l_k + o\left(\tfrac{1}{n}\right)\right) n\rho_{\boldsymbol{l}}(\boldsymbol{E}^{(n)}(t-))\, d(nt) \\ &\qquad = n\sum_j \frac{\partial \pi_i}{\partial x_j}(\hat{\boldsymbol{E}}^{(n)}(t-))F_j^{(n)}(\hat{\boldsymbol{E}}(t-))\, dt + \frac{1}{2}\sum_j\sum_k \frac{\partial^2\pi_i}{\partial x_j \partial x_k}(\hat{\boldsymbol{E}}^{(n)}(t-))a_{jk}(\hat{\boldsymbol{E}}^{(n)}(t-))\, dt, \end{aligned}$$

where, because we have rescaled time, $d(nt)$ replaces dt in probability of a jump at t. Recalling $\boldsymbol{F}(\hat{\boldsymbol{E}}(t)) = 0$ and (S.7), this yields the first term of the previous equation yields the first term of Eq (S.16).

A picture (Figure S.2) more immediately explains the emergence of the variance induced drift: unless the flow lines are parallel, jumps of identical magnitude and direction will be returned to the manifold Ω at different distances from the initial point, as one moves along the manifold:

Of course the rigorous way of obtaining the result is to use Itô's formula as done in the proof.

Proof. The weak convergence $\hat{\boldsymbol{E}}^{(n)} \Rightarrow \hat{\boldsymbol{E}}$ is proven in [19] (see [27] for an informal, applications-oriented discussion).

To characterise the limit $\hat{\boldsymbol{E}}$, we shall make use of $\boldsymbol{\pi}$. Unfortunately, $\boldsymbol{\pi}(\boldsymbol{x})$ is impossible to compute analytically, but we can still use it to obtain an SDE for $\hat{\boldsymbol{E}}(t)$. We first observe that if $\boldsymbol{F}$ is twice-continuously differentiable, then $\boldsymbol{\pi}$ is as well [18]. The continuity of $\boldsymbol{\pi}$ then tells us that $\boldsymbol{\pi}(\hat{\boldsymbol{E}}^{(n)}(t)) \Rightarrow \boldsymbol{\pi}(\hat{\boldsymbol{E}}(t))$ as well. Since $\boldsymbol{\pi}$ has first and second derivatives, we may apply Itô's formula (see Section 4) to $\boldsymbol{\pi}(\hat{\boldsymbol{E}}^{(n)}(t))$:

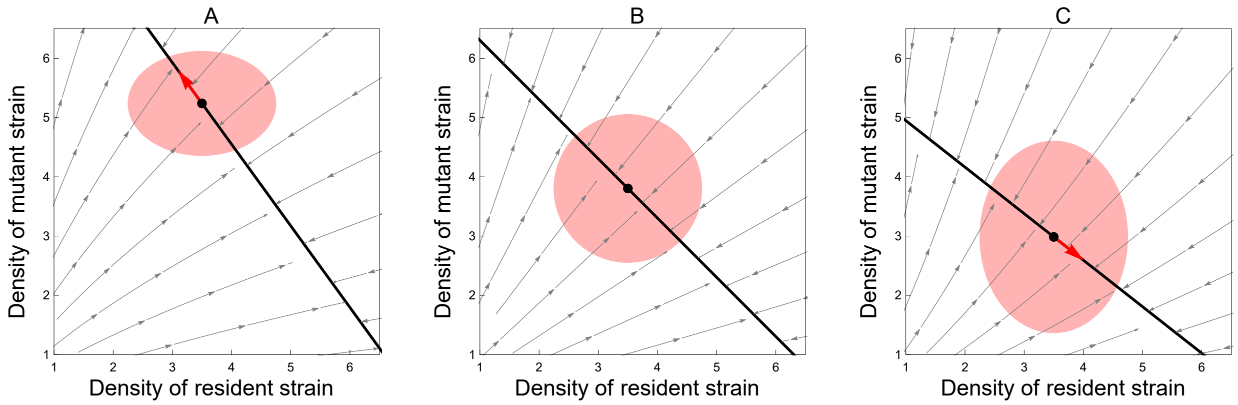

Figure S.2: Dynamics of the densities of the resident and the mutant strain in the phase plane when the two strains have the same basic reproduction number R_0, but under three different scenarios. (A) The mutant strain has a lower virulence than the resident. (B) The two strains have the same virulence. (C) The mutant strain has a higher virulence than the resident. The deterministic trajectories are shown as grey arrows that point towards the manifold Ω (the black line). The light red ellipsoid has axes proportional to the infinitesimal variance of the jumps that displace each strain from a given point on the manifold (black dot). The combination of the effect of stochasticity and the fast deterministic return to the manifold generates a drift (red arrow) that favours the strain with the lower virulence. Parameter values of the resident: $\beta_1 = 10$, $\alpha_1 = 2$, $\delta = 0.05$, $\gamma = 0.5$. Virulence of the mutant: $\alpha_2 = 1.25$, 2, and 2.75 in A, B and C, respectively.

$$
\begin{aligned}
\pi_i(\hat{\boldsymbol{E}}^{(n)}(t)) &= \pi_i(\hat{\boldsymbol{E}}^{(n)}(0)) \\
&\quad + \int_0^t \sum_{j=0}^{d+1} n \frac{\partial \pi_i}{\partial x_j}(\boldsymbol{E}^{(n)}(s)) F_j^{(n)}(\hat{\boldsymbol{E}}^{(n)}(s)) + \frac{1}{2}\sum_{j,k=0}^{d+1} n a_{jk}^{(n)}(\hat{\boldsymbol{E}}^{(n)}(s)) \frac{\partial \pi_i}{\partial x_j \partial x_k}(\boldsymbol{E}^{(n)}(s))\, ds \\
&\quad + \int_0^t \frac{1}{n}\sum_{j=0}^{d+1} \frac{\partial \pi_i}{\partial x_j}(\boldsymbol{E}^{(n)}(s))\, dM_j^{(n)}(ns) + \varepsilon_i^{(n)}(nt) \qquad \text{(S.18)}
\end{aligned}
$$

where, as before, $a_{jk}^{(n)}(\boldsymbol{x})$ is given by (S.6) and $\varepsilon_i^{(n)}(nt)$ is a smaller order error term.

On first inspection, it might appear that the drift term, which is multiplied by n, explodes as $n \to \infty$; however, from the definition of $\boldsymbol{\pi}$, we see that $\boldsymbol{\pi}(\boldsymbol{E}(t, \boldsymbol{x})) = \boldsymbol{\pi}(\boldsymbol{x})$, and thus,

$$
0 = \frac{d}{dt}\bigg|_{t=0} \boldsymbol{\pi}(\boldsymbol{E}(t, \boldsymbol{x})) = \sum_{j=0}^{d+1} \frac{\partial \pi_i}{\partial x_j}(\boldsymbol{x}) F_j(\boldsymbol{x}),
$$

and the terms of order $\mathcal{O}(n)$ vanish identically.

We can thus replace $\boldsymbol{F}^{(n)}$ by $\boldsymbol{F}^{(n)} - \boldsymbol{F}$ in (S.18), leaving

$$
\sum_{j=0}^{d+1} \frac{\partial \pi_i}{\partial x_j}(\boldsymbol{E}^{(n)}(s)) n \left(F_j^{(n)}(\hat{\boldsymbol{E}}^{(n)}(s)) - F_j(\hat{\boldsymbol{E}}^{(n)}(s)) \right),
$$

which remains bounded, as our assumptions (S.1) guarantee that $\boldsymbol{F}^{(n)} - \boldsymbol{F}$ is $\mathcal{O}\left(\frac{1}{n}\right)$.

Next, we recall (S.2)

$$
\begin{aligned}
\boldsymbol{M}^{(n)}(t) &= (\boldsymbol{e}_0 + \boldsymbol{e}_{d+1}) M_{\boldsymbol{e}_0 + \boldsymbol{e}_{d+1}}^{(n)}(t) - (\boldsymbol{e}_0 + \boldsymbol{e}_{d+1}) M_{-\boldsymbol{e}_0 - \boldsymbol{e}_{d+1}}^{(n)}(t) \\
&\quad + \sum_{i=1}^{d} (\boldsymbol{e}_i - \boldsymbol{e}_0) M_{-\boldsymbol{e}_0 + \boldsymbol{e}_i}^{(n)}(t) - \sum_{i=1}^{d} (\boldsymbol{e}_i + \boldsymbol{e}_{d+1}) M_{-\boldsymbol{e}_i - \boldsymbol{e}_{d+1}}^{(n)}(t) - \sum_{i=1}^{d} \boldsymbol{e}_i M_{-\boldsymbol{e}_i}^{(n)}(t) - \boldsymbol{e}_{d+1} M_{-\boldsymbol{e}_{d+1}}^{(n)}(t),
\end{aligned}
$$

where, for example,

$$
M_{-\boldsymbol{e}_0 - \boldsymbol{e}_{d+1}}^{(n)}(t) = \tilde{P}_{-\boldsymbol{e}_0 - \boldsymbol{e}_{d+1}} \left(\int_0^t \delta^{(n)} S^{(n)}(s)\, ds \right).
$$

Thus,

$$
\begin{aligned}
\frac{1}{n} M_{-\boldsymbol{e}_0 - \boldsymbol{e}_{d+1}}^{(n)}(nt) &= \tilde{P}_{-\boldsymbol{e}_0 - \boldsymbol{e}_{d+1}} \left(\int_0^{nt} \delta^{(n)} S^{(n)}(s)\, ds \right) \\
&= \frac{1}{n} \tilde{P}_{-\boldsymbol{e}_0 - \boldsymbol{e}_{d+1}} \left(n^2 \int_0^t \delta^{(n)} \bar{S}^{(n)}(ns)\, ds \right).
\end{aligned}
$$

The latter is a stochastic process with jumps of order $\frac{1}{n}$ and variance

$$
\int_0^t \delta^{(n)} \bar{S}^{(n)}(ns)\, ds = \int_0^t \delta^{(n)} \hat{E}_0^{(n)}(s)\, ds.
$$

Thus, as $n \to \infty$, $\frac{1}{n} M^{(n)}_{-\boldsymbol{e}_0 - \boldsymbol{e}_{d+1}}(nt)$ approaches a continuous stochastic process with variance

$$\int_0^t \delta \hat{E}_0(s)\, ds.$$

The martingale central limit theorem (see *e.g.,* [14]) tells us that the only stochastic process with these properties is a Brownian motion with the same variance,

$$\int_0^t \sqrt{\delta \hat{E}_0(s)}\, dB_{-\boldsymbol{e}_0 - \boldsymbol{e}_{d+1}}(s).$$

(*i.e.,* $B_{-\boldsymbol{e}_0 - \boldsymbol{e}_{d+1}}(t)$ is a standard Brownian motion with mean 0 and variance t).

Proceeding similarly, in the limit, we may replace all the terms $M^{(n)}_{\boldsymbol{l}}$ with integrals of independent Brownian motions, so that as $n \to \infty$, $\frac{1}{n}\boldsymbol{M}^{(n)}(nt)$ aproaches

$$\int_0^t \sigma(\hat{\boldsymbol{E}}(s))\, d\boldsymbol{B}(s)$$

where

$$\boldsymbol{B}(t) = \big(B_{\boldsymbol{e}_0 + \boldsymbol{e}_{d+1}}(t), B_{-\boldsymbol{e}_0 - \boldsymbol{e}_{d+1}}(t), B_{-\boldsymbol{e}_0 + \boldsymbol{e}_1}(t), B_{-\boldsymbol{e}_1 - \boldsymbol{e}_{d+1}}(t), B_{-\boldsymbol{e}_1}, (t), \\ \ldots, B_{-\boldsymbol{e}_0 + \boldsymbol{e}_d}(t), B_{-\boldsymbol{e}_d - \boldsymbol{e}_{d+1}}(t), B_{-\boldsymbol{e}_d}(t), B_{-\boldsymbol{e}_{d+1}}(t)\big)$$

is an ordered list of the D Brownian motions corresponding to the D noises $M^{(n)}_{\boldsymbol{l}}(t)$ and $\boldsymbol{\sigma}(\boldsymbol{x})$ is as in the statement. Taking the limit as $n \to \infty$ on both sides of (S.18) and recalling that $\boldsymbol{\pi}(\hat{\boldsymbol{E}}(t)) = \hat{\boldsymbol{E}}(t)$, we obtain (S.16). □

While the drift terms seem rather mysterious, they may be interpreted geometrically. We first observe that

Proposition 7. $(\boldsymbol{D\pi})(\boldsymbol{x})$ *is the projection onto the tangent space to* Ω *at* $\boldsymbol{x}$, $T_{\boldsymbol{x}}\Omega$.

Proof. We first observe that, since $\boldsymbol{\pi}(\boldsymbol{x}) \in \Omega$ for all $\boldsymbol{x}$, we must have

$$\boldsymbol{\pi}(\boldsymbol{\pi}(\boldsymbol{x})) = \boldsymbol{\pi}(\boldsymbol{x}).$$

If, moreover, $\boldsymbol{x} \in \Omega$, we also have $\boldsymbol{\pi}(\boldsymbol{\pi}(\boldsymbol{x})) = \boldsymbol{x}$, so taking derivatives on left and right, using the chain rule, we have that

$$(\boldsymbol{D\pi})(\boldsymbol{\pi}(\boldsymbol{x}))(\boldsymbol{D\pi})(\boldsymbol{x}) = \mathbb{I},$$

where $\mathbb{I}$ denotes the identity matrix. Now, since $\boldsymbol{x} \in \Omega$, the right hand side is equal to

$$(\boldsymbol{D\pi})(\boldsymbol{x})(\boldsymbol{D\pi})(\boldsymbol{x}),$$

so we have that $(\boldsymbol{D\pi})(\boldsymbol{x})$ is a projection. It remains to see that it is a projection onto the tangent space. We will do so by showing it's image contains, and is contained by, the tangent space.

For the former, we recall that a vector $\boldsymbol{X}$ is in the tangent space to Ω if and only if there exists a

parametric curve $\sigma_{\boldsymbol{x},\boldsymbol{X}}(t)$ such that

(i) $\sigma_{\boldsymbol{x},\boldsymbol{X}}(0) = \boldsymbol{x}$,

(ii) $\dot{\sigma}_{\boldsymbol{x},\boldsymbol{X}}(0) = \boldsymbol{X}$, and,

(iii) $\sigma_{\boldsymbol{x},\boldsymbol{X}}(t) \in \Omega$ for all $t \in \mathbb{R}$.

We then have $\pi(\sigma_{\boldsymbol{x},\boldsymbol{X}}(t)) = \sigma_{\boldsymbol{x},\boldsymbol{X}}$, and thus

$$(\boldsymbol{D\pi})(\boldsymbol{x})\boldsymbol{X} = \left.\frac{d}{dt}\right|_{t=0} \pi(\sigma_{\boldsymbol{x},\boldsymbol{X}}(t)) = \dot{\sigma}_{\boldsymbol{x},\boldsymbol{X}}(0) = \boldsymbol{X},$$

and thus $T_{\boldsymbol{x}}\Omega$ is in the image of $(\boldsymbol{D\pi})(\boldsymbol{x})$.

On the other hand, since $\boldsymbol{\pi}(\boldsymbol{x}) \in \Omega$, we have $\boldsymbol{F}(\boldsymbol{\pi}(\boldsymbol{x})) = \boldsymbol{0}$, and again, taking derivatives using the chain rule, we have

$$(\boldsymbol{DF})(\boldsymbol{\pi}(\boldsymbol{x}))(\boldsymbol{D\pi})(\boldsymbol{x}) = \boldsymbol{0},$$

so that if $\boldsymbol{x} \in \Omega$, we have $(\boldsymbol{DF})(\boldsymbol{x})(\boldsymbol{D\pi})(\boldsymbol{x}) = \boldsymbol{0}$ and thus

$$(\boldsymbol{DF})(\boldsymbol{x})(\boldsymbol{D\pi})(\boldsymbol{x})\boldsymbol{X} = \boldsymbol{0}$$

i.e., the image of $(\boldsymbol{D\pi})(\boldsymbol{x})$ is contained in the kernel of $(\boldsymbol{DF})(\boldsymbol{x})$, which we have already observed is $T_{\boldsymbol{x}}\Omega$. Thus, $\operatorname{Im}((\boldsymbol{D\pi})(\boldsymbol{x})) = T_{\boldsymbol{x}}\Omega$. □

Thus, the drift vector $(\boldsymbol{D\pi})(\boldsymbol{x})\boldsymbol{f}(\boldsymbol{x})$ from (S.17) is the projection of the vector $\boldsymbol{f}(x)$ onto the tangent space to Ω. This is an immediate consequence of the strong drift: in the absence of constraints, the process would move (on average) in the direction of this vector, whose components are the relative fitness of each strain, multiplied by the density of that strain. However, density limitation prevents unlimited growth, confining the process to the manifold Ω, and thus the direction of motion to the tangent plane, and the strains experience a drift that is the best approximating vector to their unconstrained growth rates.

5.3.1 Computing the derivatives of $\boldsymbol{\pi}$

To complete our derivation of the equations for the limiting process $\hat{\boldsymbol{E}}(t)$, we must compute the derivatives of the π_i.

Proposition 8. *Let $\boldsymbol{x} \in \Omega$ and $0 \leq i \leq d+1$. The first partial derivatives of $\boldsymbol{\pi_i}$ at $\boldsymbol{x}$ are given by $\frac{\partial \pi_i}{\partial x_k} = 0$ if $k = 0$ or $d+1$, otherwise by*

$$\frac{\partial \pi_i}{\partial x_k} = \mathbb{1}_{\{i=k\}} - \frac{(\beta_k - \alpha_k)\beta_i x_i}{\sum_{j=1}^{d}(\beta_j - \alpha_j)\beta_j x_j}.$$

The second partial derivatives $\boldsymbol{\pi_i}$ at $\boldsymbol{x}$ are given for any k, n both different from 0 and $d+1$, by:

$$\frac{\partial^2 \pi_i}{\partial x_k \partial x_n} = \frac{\beta_i}{\sum_{j=1}^{d}(\beta_j - \alpha_j)\beta_j x_j} \left(-(\beta_k - \alpha_k)\mathbb{1}_{\{n=i\}} - (\beta_n - \alpha_n)\mathbb{1}_{\{k=i\}}\right.$$

$$+\frac{(\beta_k-\alpha_k)(\beta_n-\alpha_n)x_i}{\sum_{j=1}^d(\beta_j-\alpha_j)\beta_j x_j}\left(\beta_k+\beta_n+\beta_i-\frac{\sum_{j=1}^d(\beta_j-\alpha_j)\beta_j^2 x_j}{\sum_{j=1}^d(\beta_j-\alpha_j)\beta_j x_j}\right)\Bigg).$$

Proof. We recall that under the weak selection hypothesis,

$$\dot{I}_i(t)=\beta_i\left(\frac{S(t)}{N(t)}-\frac{1}{R_0^\star}\right)I_i(t),$$

so that

$$\frac{dI_i}{dI_j}=\frac{\beta_i I_i}{\beta_j I_j}.$$

We can solve this to obtain

$$\frac{1}{\beta_i}\ln\left(\frac{I_i(t)}{I_i(0)}\right)=\frac{1}{\beta_j}\ln\left(\frac{I_j(t)}{I_j(0)}\right),$$

for all i,j, *i.e.,*

$$\frac{1}{\beta_i}\ln\left(\frac{I_i(t,\boldsymbol{x})}{x_i}\right)=\frac{1}{\beta_j}\ln\left(\frac{I_j(t,\boldsymbol{x})}{x_j}\right),$$

and, taking the limit as $t\to\infty$,

$$\frac{1}{\beta_i}\ln\left(\frac{\pi_i(\boldsymbol{x})}{x_i}\right)=\frac{1}{\beta_j}\ln\left(\frac{\pi_j(\boldsymbol{x})}{x_j}\right). \tag{S.19}$$

Taking derivatives, we then have

$$\begin{aligned}\frac{1}{\beta_i}\left(\frac{1}{\pi_i}\frac{\partial\pi_i}{\partial x_k}-\frac{1}{x_i}\mathbb{1}_{\{k=i\}}\right)&=\frac{1}{\beta_j}\left(\frac{1}{\pi_j}\frac{\partial\pi_j}{\partial x_k}-\frac{1}{x_j}\mathbb{1}_{\{k=j\}}\right),\\ \frac{1}{\beta_i\pi_i}\frac{\partial\pi_i}{\partial x_0}&=\frac{1}{\beta_j\pi_j}\frac{\partial\pi_j}{\partial x_0}\\ \frac{1}{\beta_i\pi_i}\frac{\partial\pi_i}{\partial x_{d+1}}&=\frac{1}{\beta_j\pi_j}\frac{\partial\pi_j}{\partial x_{d+1}},\end{aligned}$$

and

$$\begin{aligned}&\frac{1}{\beta_i}\left(-\frac{1}{\pi_i^2}\frac{\partial\pi_i}{\partial x_k}\frac{\partial\pi_i}{\partial x_n}+\frac{1}{\pi_i}\frac{\partial^2\pi_i}{\partial x_k\partial x_n}+\frac{1}{x_i^2}\mathbb{1}_{\{k=i\}}\mathbb{1}_{\{n=i\}}\right)\\ &\quad=\frac{1}{\beta_j}\left(-\frac{1}{\pi_j^2}\frac{\partial\pi_j}{\partial x_k}\frac{\partial\pi_j}{\partial x_n}+\frac{1}{\pi_j}\frac{\partial^2\pi_j}{\partial x_k\partial x_n}+\frac{1}{x_j^2}\mathbb{1}_{\{j=k\}}\mathbb{1}_{\{j=n\}}\right).\end{aligned}$$

Moreover, using (S.14) we have that

$$\sum_{i=1}^d(\beta_i-\alpha_i)\pi_i(\boldsymbol{x})=\lambda(R_0^\star-1),$$

so that

$$\sum_{i=1}^{d}(\beta_i - \alpha_i)\frac{\partial \pi_i}{\partial x_k} = \sum_{i=1}^{d}(\beta_i - \alpha_i)\frac{\partial \pi_i}{\partial x_0} = \sum_{i=1}^{d}(\beta_i - \alpha_i)\frac{\partial \pi_i}{\partial x_{d+1}} = 0$$

and

$$\sum_{i=1}^{d}(\beta_i - \alpha_i)\frac{\partial^2 \pi_i}{\partial x_k \partial x_n} = 0.$$

Together, these equations give us systems of linear equations that may be solved for the various derivatives of $\pi_i(\boldsymbol{x})$. To illustrate, consider $\frac{\partial \pi_i}{\partial x_0}$; from the above, we have that

$$0 = \sum_{i=1}^{d}(\beta_i - \alpha_i)\frac{\partial \pi_i}{\partial x_0} = (\beta_1 - \alpha_1)\frac{\partial \pi_1}{\partial x_0} + \sum_{i=2}^{d}(\beta_i - \alpha_i)\frac{\beta_i \pi_i}{\beta_1 \pi_1}\frac{\partial \pi_1}{\partial x_0} = \left(\sum_{i=1}^{d}(\beta_i - \alpha_i)\frac{\beta_i \pi_i}{\beta_1 \pi_1}\right)\frac{\partial \pi_1}{\partial x_0},$$

whence $\frac{\partial \pi_1}{\partial x_0} = 0$, and thus $\frac{\partial \pi_i}{\partial x_0} = 0$ for all i. Proceeding in the same manner, we find $\frac{\partial \pi_i}{\partial x_{d+1}} = 0$ as well, and thus that all second derivatives of $\pi_i(\boldsymbol{x})$ involving x_0 or x_{d+1} vanish identically, whilst

$$\frac{\partial \pi_i}{\partial x_k} = \frac{\pi_i}{x_i}\mathbb{1}_{\{i=k\}} - \frac{\pi_k}{x_k}\frac{(\beta_k - \alpha_k)\beta_i \pi_i}{\sum_{j=1}^{d}(\beta_j - \alpha_j)\beta_j \pi_j}.$$

We shall only need to evaluate these for $\boldsymbol{x} \in \Omega$, where $\hat{\boldsymbol{E}}(t)$ is trapped. For such $\boldsymbol{x}$, the first derivatives simplify to the expression given in the statement, since $\boldsymbol{\pi}(\boldsymbol{x}) = \boldsymbol{x}$ for $\boldsymbol{x} \in \Omega$. Similar calculations lead to the second partial derivatives. □

5.3.2 Reduced Diffusion

We can use the results of the previous section to provide semi-explicit expressions for the SDE satisfied by $\hat{\boldsymbol{E}}$ and displayed in Proposition 6.

Proposition 9. *Unlike the full stochastic SIR model, the weak selection limit $\hat{\boldsymbol{E}}$ can be completely characterised by a system of equations that depend only on the variables $\hat{I}_1, \ldots, \hat{I}_d$:*

$$d\hat{I}_i = s_i(\boldsymbol{I}(t))\hat{I}_i(t)\,dt + \frac{1}{\sqrt{R_0^\star}}\sum_{k=1}^{d}\left(\mathbb{1}_{\{i=k\}} - \frac{(\beta_k - \alpha_k)\beta_i \hat{I}_i(t)}{\sum_{j=1}^{d}(\beta_j - \alpha_j)\beta_j \hat{I}_j(t)}\right)\sqrt{2\beta_k \hat{I}_k(t)}\,dB_k(t). \tag{S.20}$$

where

$$s_i(\boldsymbol{x}) = \hat{s}_i(\boldsymbol{x}) - \frac{\beta_i x_i}{\sum_{j=1}^{d}(\beta_j - \alpha_j)\beta_j x_j}\sum_{j=1}^{d}(\beta_j - \alpha_j)\hat{s}_j(\boldsymbol{x}) \tag{S.21}$$

for

$$\hat{s}_i(\boldsymbol{x}) := \frac{\beta_i}{R_0^\star}\left(r_i - \frac{1}{\sum_{j=1}^{d}(\beta_j - \alpha_j)\beta_j x_j}\left(2(\beta_i - \alpha_i) - \frac{\sum_{j=1}^{d}(\beta_j - \alpha_j)^2\beta_j x_j}{\sum_{j=1}^{d}(\beta_j - \alpha_j)\beta_j x_j}\right)\right), \tag{S.22}$$

and $dB_1(t), \ldots, dB_D(t)$ are independent Brownian motions.

Proof. In the previous section, we observed that $\frac{\partial \pi_i}{\partial x_0} = \frac{\partial \pi_i}{\partial x_{d+1}} = 0$, and thus any second partial derivative with respect to x_0 or x_{d+1} vanishes as well. Moreover, for $i = 1, \ldots, d$,

$$n\left(F_i^{(n)}(\boldsymbol{x}) - F_i(\boldsymbol{x})\right) = n\left(\beta_i^{(n)}\left(\frac{x_0}{x_{d+1}} - \frac{1}{R_{0,i}^{(n)}}\right)x_i - \beta_i\left(\frac{x_0}{x_{d+1}} - \frac{1}{R_0^\star}\right)x_i\right)$$
$$\to -\frac{\beta_i r_i x_i}{R_0^\star},$$

Moreover, for $\boldsymbol{x} \in \Omega$, we have

$$\frac{x_0}{x_{d+1}} = \frac{1}{R_0^\star}\left(= \frac{\delta + \alpha_j + \gamma_j}{\beta_j}\right),$$

so that, from (S.6), we obtain in the limit $n \to \infty$

$$a_{jk}(\boldsymbol{x}) = \begin{cases} \frac{2\beta_i x_j}{R_0^\star} & \text{if } j = k, \text{ and} \\ 0 & \text{otherwise.} \end{cases}$$

Similarly, for $1 \leq j, k \leq d$, $\sigma_{jk}(\boldsymbol{x})$ depends on x_0 or x_{d+1} only via the ratio $\frac{x_0}{x_{d+1}}$ which is identically equal to $\frac{1}{R_0^\star}$ on $\boldsymbol{x} \in \Omega$.

Substituting these and the first derivatives into (S.16) allows us to complete the description of the weak limit $\hat{\boldsymbol{E}}(t)$, exploiting the fact that the triples of Brownian motions $B_{-\boldsymbol{e}_0+\boldsymbol{e}_i}$, $B_{-\boldsymbol{e}_i-\boldsymbol{e}_{d+1}}$ and $B_{-\boldsymbol{e}_j}$ and $B_{-\boldsymbol{e}_0+\boldsymbol{e}_j}$, $B_{-\boldsymbol{e}_j-\boldsymbol{e}_{d+1}}$ and $B_{-\boldsymbol{e}_i}$ are independent for $i \neq j$ to combine each triple into a single Brownian motion. □

5.3.3 Frequency Process

Repeating the argument of Section 4, we can use the functions Π_i to finding an equation for the frequency of strain i,

$$P_i(t) = \frac{\hat{I}_i(t)}{\sum_{j=1}^d \hat{I}_j(t)}$$

where, because the limiting process is a diffusion, the standard Itô formula applies. We omit the lengthy calculations this entails, and present simply the result.

For our process $\boldsymbol{P}(t)$, we find that

$$dP_i(t) = b_i(\boldsymbol{P}(t))\,dt + \frac{1}{\sqrt{R_0^\star}} \frac{1}{\sqrt{I_e(\boldsymbol{P}(t))}} \frac{1}{\sum_{j=1}^d (\beta_j - \alpha_j)\beta_j P_j(t)}$$
$$\times \sum_{j=1}^d (\mathbb{1}_{\{i=j\}} - P_i(t)) \sum_{k=1}^d (\beta_k - \alpha_k)\left(\beta_k P_k(t)\sqrt{2\beta_j P_j(t)}\,dB_j(t) - \beta_j P_j(t)\sqrt{2\beta_k P_k(t)}\,dB_k(t)\right). \quad \text{(S.23)}$$

where

$$b_i(\boldsymbol{p}) := p_i \left(s_i(I_e(\boldsymbol{p})\boldsymbol{p}) - \sum_{m=1}^{d} s_d(I_e(\boldsymbol{p})\boldsymbol{p})p_d \right),$$

for $\boldsymbol{s}(\boldsymbol{x})$ as defined by (S.21) and (S.22), and where, if $\boldsymbol{p} \in \Delta_d$ corresponds to the point $\boldsymbol{x} \in \Omega$, *i.e.,*

$$p_i := \frac{x_i}{\sum_{j=1}^{d} x_j},$$

then

$$I_e(\boldsymbol{x}) = \sum_{j=1}^{d} x_j.$$

Writing

$$p_i := \frac{x_i}{\sum_{j=1}^{d} x_j} = \frac{x_i}{I_e(\boldsymbol{x})},$$

and recalling that

$$\lambda(R_0^\star - 1) = \sum_{i=1}^{d} (\beta_i - \alpha_i)x_i = \sum_{i=1}^{d} (\beta_i - \alpha_i)I_e(\boldsymbol{x})p_i$$

we see that we can explicitly express I_e as a function of $\boldsymbol{p}$:

$$I_e(\boldsymbol{p}) = \frac{\lambda(R_0^\star - 1)}{\sum_{i=1}^{d} (\beta_i - \alpha_i)p_i}.$$

Remark 3. The notation above has been deliberately chosen to evoke the Wright-Fisher diffusion in population genetics, with $s_i(\boldsymbol{p})$ and $I_e(\boldsymbol{p})$ a frequency-dependent selection coefficient and an effective population size, respectively. To understand the motivation for the latter, note that

$$I_e = \lim_{n \to \infty} \frac{1}{n} \sum_{j=1}^{d} I_j^{(n)}(t),$$

so that $I_e(\boldsymbol{x})$ is an idealization of the total density of infected individuals (which is itself a random variable) when the diffusion limit is at the point $\boldsymbol{x} \in \Omega$, or, equivalently, when the frequencies of the various strains is $\boldsymbol{p}$. In principle, additional population structure and the corresponding sampling effects could be taken into account via an "effective infected population size" much as effective population sizes are used in the Wright-Fisher model.

Remark 4. As before, vector $\boldsymbol{b}(\boldsymbol{p})$ may be interpreted geometrically as the projection of the vector

$$\begin{bmatrix} s_1(I_e(\boldsymbol{p})\boldsymbol{p})p_1 \\ \vdots \\ s_d(I_e(\boldsymbol{p})\boldsymbol{p})p_d \end{bmatrix}$$

onto the simplex Δ_d.

5.3.4 Results for $d = 2$

If we have $d = 2$ strains, then, since $P_1(t) + P_2(t) = 1$, it is sufficient to consider the frequency of the invading strain, strain 2. Writing $P(t) := P_2(t)$, the results of the previous section tell us that the generator of $P(t)$[2] is

$$\mathcal{L}f(p) = b(p)f'(p) + \frac{1}{2}a(p)f''(p),$$

where

$$\begin{aligned} b(p) := & \frac{1}{R_0^\star}(r_2-r_1)\frac{\beta_2\beta_1\left((\beta_2-\alpha_2)p+(\beta_1-\alpha_1)(1-p)\right)}{((\beta_2-\alpha_2)\beta_2 p+(\beta_1-\alpha_1)\beta_1(1-p))}p(1-p) \\ & -\frac{1}{R_0^\star}\frac{1}{I_e(p)}\frac{\beta_2\beta_1\left((\beta_2-\alpha_2)p+(\beta_1-\alpha_1)(1-p)\right)\left(\beta_2 p+\beta_1(1-p)\right)}{((\beta_2-\alpha_2)\beta_2 p+(\beta_1-\alpha_1)\beta_1(1-p))^2} \times \Big(((\beta_2-\alpha_2)\beta_2-(\beta_1-\alpha_1)\beta_1) \\ & \quad -((\beta_2-\alpha_2)-(\beta_1-\alpha_1))\left(\frac{\beta_2\beta_1\left((\beta_2-\alpha_2)p+(\beta_1-\alpha_1)(1-p)\right)}{((\beta_2-\alpha_2)\beta_2 p+(\beta_1-\alpha_1)\beta_1(1-p))}-2(\beta_2 p+\beta_1(1-p))\right)\Big)p(1-p) \end{aligned}$$

and

$$a(p) := \frac{2}{R_0^\star}\frac{1}{I_e(p)}\frac{\beta_2\beta_1\left((\beta_2-\alpha_2)p+(\beta_1-\alpha_1)(1-p)\right)^2\left(\beta_2 p+\beta_1(1-p)\right)}{((\beta_2-\alpha_2)\beta_2 p+(\beta_1-\alpha_1)\beta_1(1-p))^2}p(1-p).$$

The generator allows us to compute many quantities of interest for the process $P(t)$. In particular, if $h(p)$ is the probability of fixation of strain 1 given $P(0) = p$, then $h(p)$ satisfies the boundary problem

$$\begin{aligned} \mathcal{L}h(p) &= 0 \\ h(0) &= 0 \\ h(1) &= 1 \end{aligned}$$

[2]The generator of $\boldsymbol{P}(t)$ is the operator on the space of continuous functions on the d-simplex

$$\Delta_d = \left\{\boldsymbol{p} : \sum_{i=1}^d p_i = 1\right\}$$

defined by

$$\mathcal{L}f(\boldsymbol{p}) := \lim_{t\downarrow 0}\frac{\mathbb{E}\left[f(\boldsymbol{P}(t))|\boldsymbol{P}(0)=\boldsymbol{p}\right]-f(\boldsymbol{p})}{t}.$$

We recall that if the diffusion process $\boldsymbol{P}(t)$ has SDE

$$d\boldsymbol{P}(t) = \boldsymbol{b}(\boldsymbol{P}(t)))\,dt + \boldsymbol{\varsigma}(\boldsymbol{P}(t)))\,d\boldsymbol{B}(t),$$

then

$$\mathcal{L}f(\boldsymbol{p}) = \sum_{i=1}^d b_i(\boldsymbol{p})\frac{\partial f}{\partial p_i} + \frac{1}{2}\sum_{i=1}^d\sum_{i=1}^d a_{ij}(\boldsymbol{p})\frac{\partial^2 f}{\partial p_i \partial p_j}$$

where $\boldsymbol{a}(\boldsymbol{x}) = \boldsymbol{\varsigma}(\boldsymbol{x})\boldsymbol{\varsigma}(\boldsymbol{x})^\top$ is the variance-covariance matrix for $d\boldsymbol{P}(t)$, and the probability density function for $\boldsymbol{P}(t)$, say $f(t,\boldsymbol{p})$, satisfies the Kolmogorov backward equation

$$\frac{\partial}{\partial t}f(t,\boldsymbol{p}) = \mathcal{L}f(t,\boldsymbol{p}).$$

(see *e.g.,* [15, 11, 13]). This may be solved to give

$$h(p) = \frac{\int_0^p e^{-2\int \frac{b(q)}{a(q)}\,dq}\,dq}{\int_0^1 e^{-2\int \frac{b(q)}{a(q)}\,dq}\,dq}. \tag{S.24}$$

Let $\tilde{h}(p)$ be the numerator of this fraction. Substituting the expressions for $a(p)$ and $b(p)$ and some simplification yields

$$\tilde{h}(p) := \int_0^p \frac{(\beta_2 q+\beta_1(1-q))^{-\frac{(\beta_2+\beta_1)((\beta_2-\alpha_2)-(\beta_1-\alpha_1))}{\beta_2\alpha_1-\beta_1\alpha_2}}\,((\beta_2-\alpha_2)q+(\beta_1-\alpha_1)(1-q))^{2-\frac{((\beta_2-\alpha_2)\beta_2-(\beta_1-\alpha_1)\beta_1)}{\beta_2\alpha_1-\beta_1\alpha_2}}}{((\beta_2-\alpha_2)\beta_2 q+(\beta_1-\alpha_1)\beta_1(1-q))} \times e^{-(r_2-r_1)\int_0^q I_e(u)\frac{((\beta_2-\alpha_2)\beta_2 u+(\beta_1-\alpha_1)\beta_1(1-u))}{((\beta_2-\alpha_2)u+(\beta_1-\alpha_1)(1-u))(\beta_2 u+\beta_1(1-u))}\,du}\,dq,$$

whereas

$$h(p) = \frac{\tilde{h}(p)}{\tilde{h}(1)}.$$

For ease of notation, we will write

$$\phi(q) = \int_0^q I_e(u)\frac{((\beta_2-\alpha_2)\beta_2 u+(\beta_1-\alpha_1)\beta_1(1-u))}{((\beta_2-\alpha_2)u+(\beta_1-\alpha_1)(1-u))\,(\beta_2 u+\beta_1(1-u))}\,du$$

and

$$g(q) = \frac{(\beta_2 q+\beta_1(1-q))^{-\frac{(\beta_2+\beta_1)((\beta_2-\alpha_2)-(\beta_1-\alpha_1))}{\beta_2\alpha_1-\beta_1\alpha_2}}\,((\beta_2-\alpha_2)q+(\beta_1-\alpha_1)(1-q))^{2-\frac{((\beta_2-\alpha_2)\beta_2-(\beta_1-\alpha_1)\beta_1)}{\beta_2\alpha_1-\beta_1\alpha_2}}}{((\beta_2-\alpha_2)\beta_2 q+(\beta_1-\alpha_1)\beta_1(1-q))},$$

so that

$$\tilde{h}(p) = \int_0^p g(q)e^{-(r_2-r_1)\phi(q)}\,dq.$$

We can evaluate this expression numerically, but we will be particularly interested in a number of special cases, when we can obtain analytical approximations to $\tilde{h}(p)$.

(i) When $r_2 = r_1$ (or, more generally, when $R_{0,i} = R_0^\star\left(1+o\left(\frac{1}{n}\right)\right)$) we can give an explicit closed form for $\tilde{h}(p)$, and thus $h(p)$:

$$\tilde{h}(p) \propto \frac{(\beta_2 p+\beta_1(1-p))^{1-\frac{(\beta_2+\beta_1)((\beta_2-\alpha_2)-(\beta_1-\alpha_1))}{\beta_2\alpha_1-\beta_1\alpha_2}}\,((\beta_2-\alpha_2)p+(\beta_1-\alpha_1)(1-p))^{3-\frac{((\beta_2-\alpha_2)\beta_2-(\beta_1-\alpha_1)\beta_1)}{\beta_2\alpha_1-\beta_1\alpha_2}}}{((\beta_2-\alpha_2)p+(\beta_1-\alpha_1)(1-p))} - \frac{(\beta_2+\beta_1)^{1-\frac{(\beta_2+\beta_1)((\beta_2-\alpha_2)-(\beta_1-\alpha_1))}{\beta_2\alpha_1-\beta_1\alpha_2}}\,((\beta_2-\alpha_2)+(\beta_1-\alpha_1))^{3-\frac{((\beta_2-\alpha_2)\beta_2-(\beta_1-\alpha_1)\beta_1)}{\beta_2\alpha_1-\beta_1\alpha_2}}}{((\beta_2-\alpha_2)+(\beta_1-\alpha_1))}. \tag{S.25}$$

This expression is not, however, especially illuminating.

(ii) We shall principally be interested in the case when p is small, in which case we can Taylor

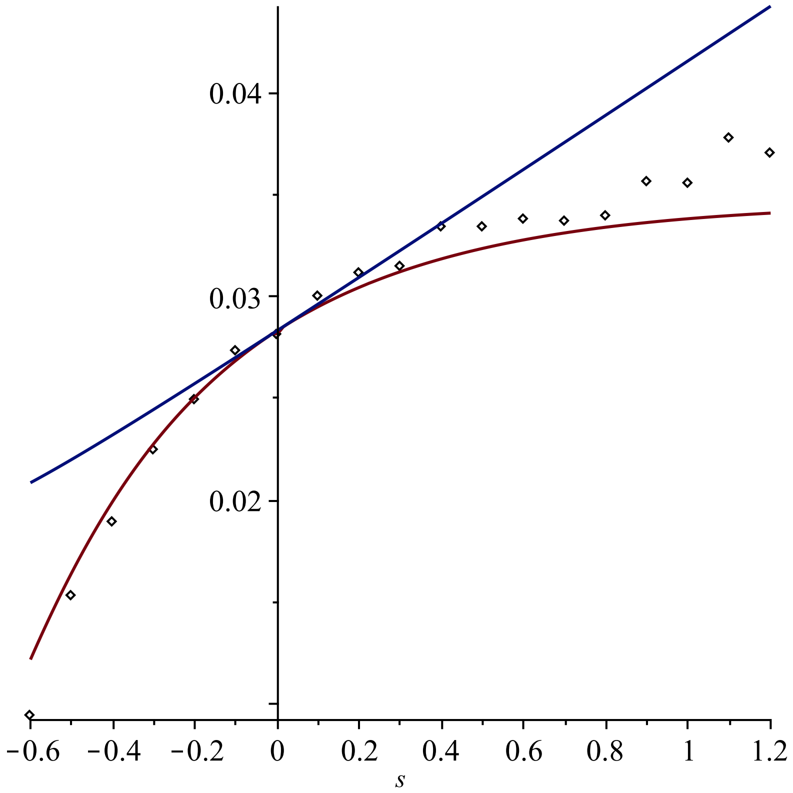

Figure S.3: We compare (S.25) (red curve) and its order p^2 approximation (S.26) (blue line) with fixation probabilities for a single mutant invader obtained via simulating the Markov chain with rates given by Table 1 (black diamonds). We vary β_2 by setting $\beta_1 = \beta_2(1+\sigma)$, while holding $R_{0,1} = R_{0,2}$ by setting $\alpha_2 = \frac{\delta+\alpha_1+\gamma}{1+\sigma} - \delta - \gamma$. The other parameters are fixed at $n = 100$, $R_0^\star = 4$, $\lambda = 2$, $\delta = 1$, $\beta_1 = 20$, and $\alpha_1 = 3$.

expand $\tilde{h}(p)$ as

$$\begin{aligned}\tilde{h}(p) &= \tilde{h}(0) + \tilde{h}'(0)p + \frac{1}{2}\tilde{h}''(0)p^2 + \mathcal{O}\big(p^3\big) \\ &= g(0)p + \frac{1}{2}(g'(0) + g(0)(r_2 - r_1)\phi'(0))p^2 + \mathcal{O}\big(P^3\big) \\ &= g(0)\left(p + \frac{1}{2}\left(\frac{g'(0)}{g(0)} + (r_2 - r_1)I_e(0)\right)p^2\right) + \mathcal{O}\big(p^3\big)\end{aligned}$$

Unfortunately, this does not yield an estimate of the normalising constant, $\tilde{h}(1)$. To obtain this, we consider the case when $\beta_2 - \beta_1$ and $\alpha_2 - \alpha_1$ are small. While this is a restrictive assumption, it will allow us to consider the long-term evolution in the framework of adaptive dynamics, where mutational changes are assumed to be very small. To this end, we introduce σ and θ such that

$$\beta_2 = \beta_1(1+\sigma) \quad \text{and} \quad \alpha_2 = \alpha_1(1+\theta).$$

We then have that

$$\frac{g'(0)}{g(0)} = \sigma + \mathcal{O}(2), \frac{g(p)}{g(0)} = 1 - \sigma p + \mathcal{O}(2), \phi'(p) = -I_e(p) + \mathcal{O}(2),$$

and

$$\begin{aligned}\frac{\tilde{h}(1)}{g(0)} &= \int_0^1 (1-\sigma p) e^{-(r_2-r_1)\int_0^p I_e(q)\,dq}\,dp + \mathcal{O}(2)\\ &= 1 - \frac{\sigma}{2} - (r_2 - r_1) I_e(0) + \mathcal{O}(2),\end{aligned}$$

where $\mathcal{O}(2)$ is used to denote terms of order $\mathcal{O}(\sigma^2)$, $\mathcal{O}(\theta^2)$, or $\mathcal{O}(\sigma\theta)$.

Then, to order p^2, the fixation probability is

$$h(p) := \frac{\tilde{h}(p)}{\tilde{h}(1)} = p + \frac{1}{2}(\sigma + (r_2 - r_1) I_e(0)) p(1-p) + \mathcal{O}(2), \tag{S.26}$$

which may be written informally in terms of the original parameters as

$$h(p) := \frac{\tilde{h}(p)}{\tilde{h}(1)} = p + \frac{1}{2}\left(\frac{\beta_1 - \beta_2}{\beta_2} + \left(1 - \frac{R_{0,1}^{(n)}}{R_{0,2}^{(n)}}\right) n I_e(0)\right) p(1-p) + \mathcal{O}(2)$$

to lowest order. Here we have used

$$r_2 - r_1 = n\frac{R_{0,2}^{(n)} - R_{0,1}^{(n)}}{R_0^\star} + o(1) = n\frac{R_{0,2}^{(n)} - R_{0,1}^{(n)}}{R_{0,2}^{(n)}}\left(1 + \frac{r_2}{n} + o(1)\right) + o(1).$$

In practice, we are most interested in the case when a single individual carries the mutant strain, so $p = \frac{1}{nI_e(0)} = \frac{1}{I^{(n)}(0)}$. While our proofs – which assume that p is independent of n, and thus that the number of invading individuals is proportional to n – do not justify taking this value for p, we find that the expression for the fixation probability obtained by taking $p = \frac{1}{I^{(n)}(0)}$, which to lowest order is

$$\frac{1}{I^{(n)}(0)} + \frac{1}{2}\left(\frac{1}{I^{(n)}(0)}\frac{\beta_1 - \beta_2}{\beta_2} + 1 - \frac{R_{0,1}^{(n)}}{R_{0,2}^{(n)}}\right), \tag{S.27}$$

agrees extremely well with simulations (Figure S.4c) – an example of the so-called "unreasonable effectiveness of mathematics' [33] – and will use it to investigate the long term evolution of the virulence in Section 6.

5.4 Reconciling the Strong and Weak Selection Results

On first inspection, our expressions for the strong and weak selection limits have little in common. In Section 5.2, we saw that if $R_{0,i}^{(n)} \to R_{0,i}$ and $R_{0,2} \neq R_{0,1}$, then, if $I_2^{(n)}(0) \to I_2(0)$, the fixation

probability was

$$\begin{cases} 1 - \left(\frac{R_{0,1}}{R_{0,2}}\right)^{I_2(0)} & \text{if } R_{0,2} > R_{0,1}\text{, and} \\ 0 & \text{otherwise} \end{cases}$$

On the other hand, in Section 5.3, we assume that

$$R_{0,i}^{(n)} = R_0^\star \left(1 + \frac{r_i}{n}\right) + o\left(\tfrac{1}{n}\right)$$

and $\bar{I}^{(n)}(0) = \frac{1}{n} I_2^{(n)}(0) \to I_2(0)$, and derive a quite different appearing expression for the fixation probability.

More generally, we might consider the intermediary scalings: let $1 \ll \kappa_n \ll n$, and suppose that

$$R_{0,i}^{(n)} = R_0^\star \left(1 + \frac{r_i}{\kappa_n}\right) + o\left(\tfrac{1}{\kappa_n}\right)$$

whilst

$$\lim_{n\to\infty} \frac{I_2^{(n)}(0)}{\kappa_n} = \iota.$$

Substituting these into our expression for strong selection, we see that, provided $r_2 > r_1$, we have that the probability of fixation is

$$1 - \left(\frac{\left(1 + \frac{r_1}{\kappa_n}\right) + o\left(\frac{1}{\kappa_n}\right)}{\left(1 + \frac{r_2}{\kappa_n}\right) + o\left(\frac{1}{\kappa_n}\right)}\right)^{\kappa_n \frac{I^{(n)}(0)}{\kappa_n}} \to 1 - e^{-(r_2 - r_1)\iota}$$

as $n \to \infty$ (*n.b.,* that when $\kappa_n \gg n$, $\frac{I_2^{(n)}(0)}{\kappa_n} \to 0$, so trivially the probability of fixation is 0).

On the other hand, we can begin with our expression for the fixation probability under weak selection, which written in terms of the original parameters $R_{0,2}^{(n)}$ and $R_{0,1}^{(n)}$, was proportional to

$$\tilde{h}(p) = \int_0^p \frac{(\beta_2 q - \beta_1(1-q))^{-\frac{(\beta_2+\beta_1)((\beta_2-\alpha_2)-(\beta_1-\alpha_1))}{\beta_2\alpha_1-\beta_1\alpha_2}} ((\beta_2-\alpha_2)q + (\beta_1-\alpha_1)(1-q))^{2-\frac{((\beta_2-\alpha_2)\beta_2-(\beta_1-\alpha_1)\beta_1)}{\beta_2\alpha_1-\beta_1\alpha_2}}}{((\beta_2-\alpha_2)\beta_2 q + (\beta_1-\alpha_1)\beta_1(1-q))} \times e^{-n\left(\frac{R_{0,2}^{(n)}-R_{0,1}^{(n)}}{R_0^\star} + o\left(\frac{1}{n}\right)\right) \frac{((\beta_2-\alpha_2)\beta_2 q+(\beta_1-\alpha_1)\beta_1(1-q))}{\beta_2\beta_1((\beta_2-\alpha_2)q+(\beta_1-\alpha_1)(1-q))(\beta_2 q+\beta_1(1-q))}\, dq}\, dq$$

Replacing $R_{0,2}^{(n)}$ and $R_{0,1}^{(n)}$ with the intermediary scalings, our expression becomes

$$\int_0^p \frac{(\beta_2 q - \beta_1(1-q))^{-\frac{(\beta_2+\beta_1)((\beta_2-\alpha_2)-(\beta_1-\alpha_1))}{\beta_2\alpha_1-\beta_1\alpha_2}} ((\beta_2-\alpha_2)q + (\beta_1-\alpha_1)(1-q))^{2-\frac{((\beta_2-\alpha_2)\beta_2-(\beta_1-\alpha_1)\beta_1)}{\beta_2\alpha_1-\beta_1\alpha_2}}}{((\beta_2-\alpha_2)\beta_2 q + (\beta_1-\alpha_1)\beta_1(1-q))} \times e^{-\frac{n}{\kappa_n}(r_2-r_1+o(1)) \int I_e(q) \frac{((\beta_2-\alpha_2)\beta_2 q+(\beta_1-\alpha_1)\beta_1(1-q))}{\beta_2\beta_1((\beta_2-\alpha_2)q+(\beta_1-\alpha_1)(1-q))(\beta_2 q+\beta_1(1-q))}\, dq}\, dq.$$

We can find a large n asymptotic expression for this probability using a pair of lemmas:
Lemma 1. *Suppose that $\phi(x)$ and $g(x)$ are an increasing continuously differentiable function and a continuous function on $[a, b]$ $(-\infty < a < b < \infty)$ respectively, and that $\phi'(a) \neq 0$. Then,*

$$\lim_{M\to\infty} \frac{\int_a^b g(x)e^{-M\phi(x)}\,dx}{\frac{g(a)e^{-M\phi(a)}}{M\phi'(a)}} = 1.$$

Proof. Fix $\varepsilon > 0$ such that $\phi'(a) > \varepsilon$. Using Taylor's theorem, we may write

$$\phi(x) = \phi(a) + \phi'(a)(x-a) + R(x)(x-a),$$

where $R(x) \to 0$ as $x \to a$. Fix $\delta > 0$ such that

$$|R(x)| < \varepsilon \quad \text{and} \quad |g(x) - g(a)| < \varepsilon$$

for all x such that $x - a < \delta$. Finally, choose $\eta > 0$ such that $\phi(x) > \phi(a) + \eta$ for all x such that $x - a \geq \delta$ and B such that $|g(x)| < B$ for all $x \in [a, b]$. Then,

$$\begin{aligned}\int_a^b g(x)e^{-M\phi(x)}\,dx &= e^{-M\phi(a)} \int_a^b g(x)e^{-M(\phi(x)-\phi(a))}\,dx \\ &= e^{-M\phi(a)} \left(\int_a^{a+\delta} g(x)e^{-M(\phi(x)-\phi(a))}\,dx + \int_{a+\delta}^b g(x)e^{-M(\phi(x)-\phi(a))}\,dx\right).\end{aligned}$$

Now,

$$\left|\int_{a+\delta}^b g(x)e^{-M(\phi(x)-\phi(a))}\,dx\right| \leq \int_{a+\delta}^b Be^{-M\eta}\,dx \to 0$$

as $M \to \infty$, whereas

$$\begin{aligned}(g(a)-\varepsilon)\int_a^{a+\delta} e^{-M(\phi'(a)+\varepsilon)(x-a)}\,dx &\leq \int_a^b g(x)e^{-M(\phi(x)-\phi(a))}\,dx \\ &\leq (g(a)+\varepsilon)\int_a^{a+\delta} e^{-M(\phi'(a)-\varepsilon)(x-a)}\,dx.\end{aligned}$$

Now, letting $y = M(x-a)$, we have

$$\int_a^{a+\delta} e^{-M(\phi'(a)-\varepsilon)(x-a)}\,dx = \frac{1}{M}\int_0^{M\delta} e^{-(\phi'(a)-\varepsilon)y}\,dy$$

whilst

$$\int_0^{M\delta} e^{-(\phi'(a)-\varepsilon)y}\,dy = \frac{1}{\phi'(a)-\varepsilon}\left(1 - e^{-(\phi'(a)-\varepsilon)M\delta}\right) \to \frac{1}{\phi'(a)-\varepsilon}$$

as $M \to \infty$, and similarly for the lower bound.

Since $\varepsilon > 0$ can be chosen arbitrarily small, the result follows. $\square$

Lemma 2. *Let $\phi(x)$, $g(x)$, and $[a,b]$ be as above. Then,*

$$\lim_{M\to\infty} \frac{\int_a^{a+\frac{X}{M}} g(x)e^{-M\phi(x)}\,dx}{\frac{g(a)e^{-M\phi(a)}}{M\phi'(a)}} = 1 - e^{-\phi'(a)X}.$$

Proof. By direct computation, we have

$$\begin{aligned}\int_a^{a+\frac{X}{M}} g(x)e^{-M\phi(x)}\,dx &= \int_0^X g(a+\frac{y}{M})e^{-M\phi(a+\frac{y}{M})}\,dy \\ &= \frac{1}{M}\int_0^X g(a+\frac{y}{M})e^{-M(\phi(a)+\phi'(a)\frac{y}{M}+R(a+\frac{y}{M})\frac{y}{M}}\,dy\end{aligned}$$

so that as $M\to\infty$,

$$\frac{\int_a^{a+\frac{X}{M}} g(x)e^{-M\phi(x)}\,dx}{\frac{g(a)e^{-M\phi(a)}}{M\phi'(a)}} \to \phi'(a)\int_0^X e^{-\phi'(a)y}\,dy.$$

The result follows. ∎

To apply the lemmas here, we take $a=0$ and $b=1$, $M_n = \frac{n}{\kappa_n}$, and, as before,

$$g(p) = \frac{(\beta_2 p - \beta_1(1-p))^{-\frac{(\beta_2+\beta_1)((\beta_2-\alpha_2)-(\beta_1-\alpha_1))}{\beta_2\alpha_1-\beta_1\alpha_2}}\,((\beta_2-\alpha_2)p+(\beta_1-\alpha_1)(1-p))^{2-\frac{((\beta_2-\alpha_2)\beta_2-(\beta_1-\alpha_1)\beta_1)}{\beta_2\alpha_1-\beta_1\alpha_2}}}{((\beta_2-\alpha_2)\beta_2 p+(\beta_1-\alpha_1)\beta_1(1-p))}$$

whereas we now take a slightly different definition for $\phi(p)$, which now has an $o(1)$ correction:

$$\phi(p) = (r_2 - r_1 + o(1))\int I_e(p)\frac{((\beta_2-\alpha_2)\beta_2 p + (\beta_1-\alpha_1)\beta_1(1-p))}{\beta_2\beta_1\left((\beta_2-\alpha_2)p+(\beta_1-\alpha_1)(1-p)\right)\left(\beta_2 p+\beta_1(1-p)\right)}\,dp$$

so that

$$\phi'(0) \to I_e(0)(r_2 - r_1)$$

as $n\to\infty$. Then, using Lemma 1 we conclude that $\tilde{h}(1)$ is asymptotically equivalent to

$$\frac{g(0)e^{-M_n\phi(0)}}{M_n\phi'(0)}.$$

Now, to consider the numerator when we start with $I_2^{(n)}(0) \sim \iota\kappa_n$ individuals of the invading strain, we recall that

$$p = \lim_{n\to\infty} P_2^{(n)}(0) = \lim_{n\to\infty}\frac{I_2^{(n)}(0)}{I_2^{(n)}(0)+I_1^{(n)}(0)} = \lim_{n\to\infty}\frac{I_2^{(n)}(0)}{I_e(P_2^{(n)}(0))n}$$

so, to apply Lemma 2, we will take

$$X := X^{(n)} = M_n\frac{I_2^{(n)}(0)}{I_e(P_2^{(n)}(0))n} = \frac{I_2^{(n)}(0)}{I_e(P^{(n)}(0))\kappa_n}$$

i.e., so that $\frac{X^{(n)}}{M_n} \sim p$.

We note that $P^{(n)}(0) \propto \frac{\kappa_n \iota}{n} \to 0$ as $n \to \infty$, so

$$\lim_{n\to\infty} X^{(n)} = \frac{\iota}{I_e(0)}.$$

Thus, applying Lemma 2, we have

$$\tilde{h}\left(\frac{I_2^{(n)}(0)}{I_e(P_2^{(n)}(0))n}\right) \sim \frac{g(0)e^{-M_n\phi(0)}}{M_n\phi'(0)}\left(1 - e^{-(r_2-r_1)\iota}\right),$$

and the probability of fixation obtained from the weak selection expression is again asymptotic to

$$1 - e^{-(r_2-r_1)\iota}.$$

While this is not a rigorous proof, it does demonstrate heuristically that the weak and strong selection expressions for the fixation probability agree to first order when applied across the intermediate selective regimes. In particular, we can use the method of matched asymptotic expansions (see *e.g.,* [17, 20]) to combine our two solutions into a single expression valid across all scales, by summing the expressions for strong and weak selection and subtracting their common limit, where all are expressed in the unscaled (*i.e.,* strong selection parameters):

$$\left[1 - \left(\frac{R_{0,1}^{(n)}}{R_{0,2}^{(n)}}\right)^{I_2^{(n)}(0)} + h\left(\frac{I_2^{(n)}(0)}{I_e(0)n}\right) - \left(1 - e^{\left(\frac{R_{0,1}^{(n)}}{R_{0,2}^{(n)}}-1\right)I_2^{(n)}(0)}\right)\right]^+, \tag{S.28}$$

where $[x]^+ = \max\{x, 0\}$ and we have used that

$$\frac{R_{0,1}^{(n)}}{R_{0,2}^{(n)}} - 1 = \frac{1+\frac{r_1}{\kappa_n}}{1+\frac{r_2}{\kappa_n}} - 1 = \frac{1}{\kappa_n}\frac{r_1 - r_2}{1+\frac{r_2}{\kappa_n}} = \frac{1}{\kappa_n}(r_1 - r_2) + \mathcal{O}\left(\frac{1}{\kappa_n^2}\right),$$

so that

$$(r_1 - r_2)\iota \sim (r_1 - r_2)\frac{I_2^{(n)}(0)}{\kappa_n} \sim \left(\frac{R_{0,1}^{(n)}}{R_{0,2}^{(n)}} - 1\right) I_2^{(n)}(0).$$

We illustrate how these approximations compare to a simulated epidemic in Figure S.4.

6 Adaptive Dynamics

Using the expressions for the fixation probability derived above, we can use the framework of adaptive dynamics to investigate the long-term evolution of strains. In what follows, we give an informal discussion of the derivation of the canonical *diffusion* for the process, a generalisation of the canonical equation of adaptive dynamics which allows us to consider the influence of random drift on phenotypic evolution. We refer the reader to [26] for a more extensive discussion

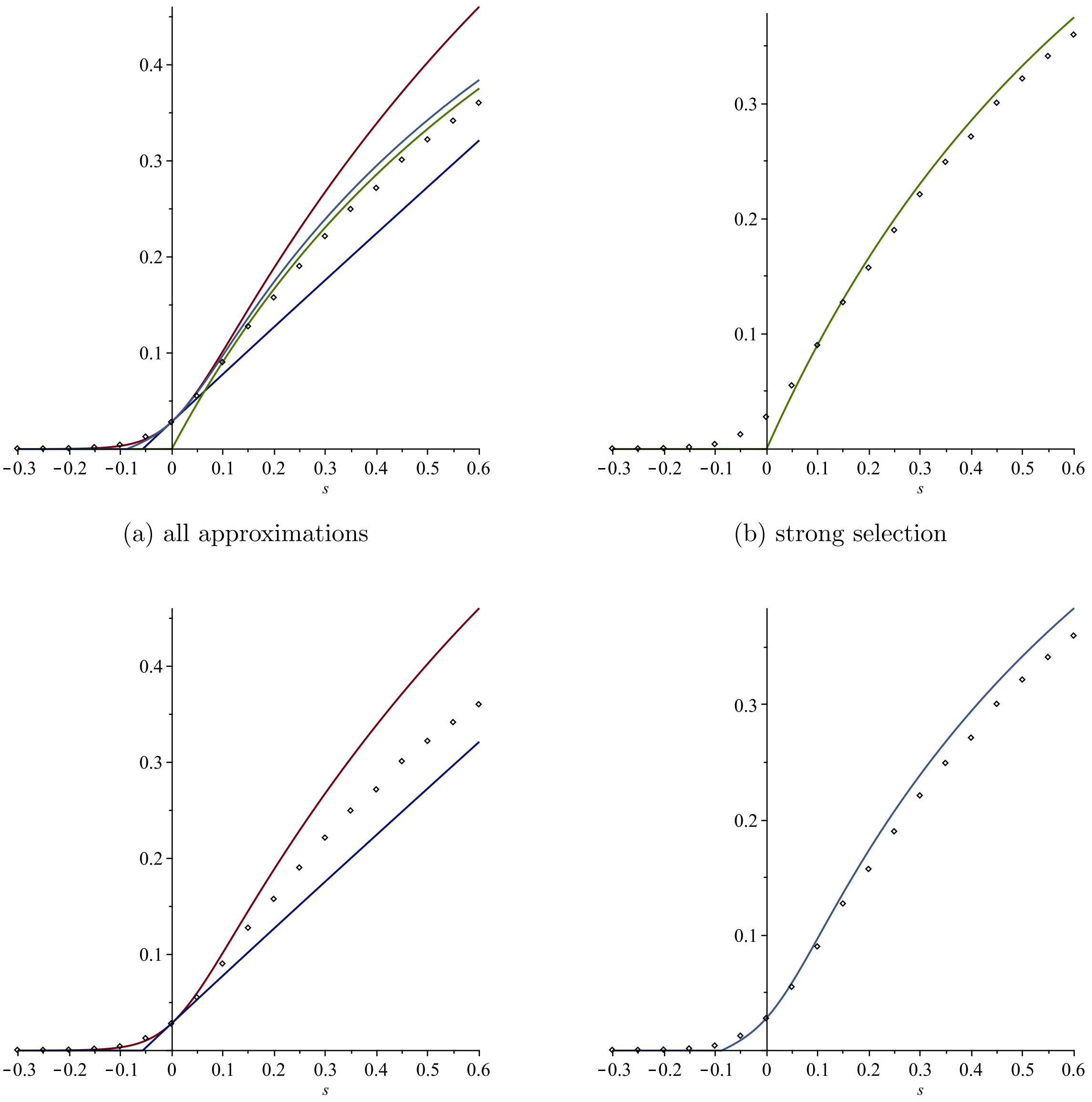

(a) all approximations

(b) strong selection

(c) weak selection

(d) matched

Figure S.4: We compare our various approximations to fixation probabilities obtained by Markov chain simulations (black diamonds), where we assume a single mutant invading a resident population at equilibrium. We have implemented selection on the invading strain by setting $\beta_2 = \beta_1(1+s)$, so that $R_{0,2}^{(n)} = R_{0,1}^{(n)}(1+s)$. The other parameters are fixed at $n = 100$, $R_{0,1}^{(n)} = R_0^\star = 4$, $\lambda = 2$, $\delta = 1$, $\beta_1 = 20$, and $\alpha_1 = 3$. (b) shows strong selection approximation (S.15) (green curve), (c) shows the weak selection approximation (S.24) (red curve) and its second order approximation (S.24) (blue line), (d) shows the matched asymptotic approximation (S.28), whereas (a) overlays all the approximations for comparison. As would be expected, the strong and weak approximations do well in their corresponding parameter regimes, but poorly elsewhere, whereas the matched asymptotic provides a compromise, performing worse than the weak or strong approximations at the respective extremes, but interpolating between them for intermediate values of s.

aimed at a biological audience and to [9] for a mathematically rigorous derivation of the canonical equation.

We briefly recall the assumptions of adaptive dynamics in the context of our epidemic models; throughout, we assume that a novel mutant strain with virulence α' invades a population which is at the endemic equilibrium with a resident strain α.

We assume a tradeoff between transmissibility and virulence, so that the contact rate of a strain depends on its virulence according to some fixed function $\beta(\alpha)$. For our numerical investigations, we take

$$\beta(\alpha) = (\delta + \alpha + \gamma)(R_{0,\max} - w(\alpha - \alpha_0)^2),$$

where w is a parameter that determines the "flatness" of the fitness landscape.

The reproductive number is a function of the virulence,

$$R_0(\alpha) = \frac{\beta(\alpha)}{\delta + \alpha + \gamma}.$$

We will assume that there is a value, α_0, for the virulence that maximises $R_0(\alpha)$.

Under these assumptions, the density of ndividuals infected with the resident strain at the endemic equilibrium is

$$I_{\mathrm{eq}}(\alpha) \sim \frac{\lambda(R_0(\alpha) - 1)}{\beta(\alpha) - \alpha}$$

$I_{\mathrm{eq}}(\alpha)$ is non-zero on a range $(\alpha_{\min}, \alpha_{\max})$; outside of this range, $R_0(\alpha) \leq 1$, and the pathogen goes extinct (when R_0 is independent of the virulence, $\alpha_{\min} = 0$; in this case, as we will see below, we must fix $\alpha_{\max} < \infty$ to have a probability distribution. The choice of $\alpha_{\max}$ is arbitrary).

We then have, using (S.27), that the fixation probability of a mutant strain of virulence α' arising in a single individual in a population in which a strain of virulence α', is

$$S(\alpha, \alpha') \sim \frac{1}{nI_{\mathrm{eq}}(\alpha)} + \frac{1}{2}\left(\frac{1}{nI_{\mathrm{eq}}(\alpha)}\frac{\beta(\alpha) - \beta(\alpha')}{\beta(\alpha')} + 1 - \frac{R_0(\alpha)}{R_0(\alpha')}\right) + \mathcal{O}\Big(|\alpha - \alpha'|^2\Big).$$

To introduce the evolutionary dynamics, we assume that mutations occur in individuals with virulence α at a per-capita rate $\epsilon\eta(\alpha)$, where $\epsilon > 0$ is a dimensionless parameter that we will take to 0. This will ensure that, with high probability, fixation occurs before a second novel mutation can arise. The population is thus assumed to be monomorphic (*i.e.,* all individuals have the same virulence) between invasion events. We will assume that given a mutation occurs in an individual α, the offspring has virulence α' with probability $K(\alpha, \alpha' - \alpha)$ where K has mean 0 and finite variance:

$$\int_{-\infty}^{\infty} K(\alpha, z) z^k \, dz = \begin{cases} 0 & \text{if } k = 1, \\ \nu(\alpha) & \text{if } k = 2, \text{ and} \\ \mathcal{O}(1) & \text{otherwise.} \end{cases}$$

We now pass from the individual based model to the trait substitution sequence [25, 10]: we have seen that whenever a new strain arises, either the mutant or resident strain will rapidly go extinct.

Until a new mutant arises, the population will be composed entirely of individuals of the surviving strain. Let $A_\epsilon(t)$ be a random variable giving the virulence of the strain that survived the last competition event prior to time t. The population is thus entirely composed of the strain $A_\epsilon(t)$ except for times t in the short intervals when two strains are competing. If we pass to a "mutational time scale", $\frac{t}{\epsilon}$, as $\epsilon \to 0$ the duration of these intervals shrinks to 0, and we are left with a process in which novel mutations either fix or disappear instantly, so that the population is only observed with a single strain at equilibrium.

Formally, as $\epsilon \to 0$, $A_\epsilon(\frac{t}{\epsilon}) \Rightarrow A(t)$, a continuous time Markov chain that jumps from virulence α to α' when a strain of virulence α' successfully invades a population with resident virulence α [9]. The process $A(t)$ has generator (recall, the generator is the operator $\mathcal{L}$ defined by $\mathcal{L}f(\alpha) := \frac{d}{dt}\big|_{t=0}\mathbb{E}[f(A(t))|A(0) = \alpha]$):

$$\mathcal{L}f(\alpha) = n\eta(\alpha)I_{\mathrm{eq}}(\alpha)\int_{\alpha_{\min}}^{\alpha_{\max}} K(\alpha,\alpha')s(\alpha,\alpha')\big(f(\alpha') - f(\alpha)\big)\, d\alpha'$$

n.b., If $\alpha' \notin [\alpha_{\min}, \alpha_{\max}]$, then the mutant cannot fix.

We now consider the limit of small mutations, and, following [8] assume that mutations will be of order $\mathcal{O}(\varepsilon)$, where ε is a dimensionless parameter which we will take to 0; this limit of small mutational effects allows us to ignore terms of order $\mathcal{O}\Big(|\alpha - \alpha'|^2\Big)$ in the fixation probability. In particular, we introduce a rescaled kernel

$$K_\varepsilon(\alpha, z) = \frac{1}{\varepsilon}K\left(\alpha, \frac{z}{\varepsilon}\right),$$

so that $K_\varepsilon(\alpha, z)\, dz$ is the probability the mutant has virulence $\alpha + \varepsilon z$. Let $A^\varepsilon(t)$ be the process defined as $A(t)$ above, but with the kernel K replaced by the kernel K_ε.

Now, consider the time rescaled process $\hat{A}_\varepsilon(t) := A^\varepsilon\left(\frac{t}{\varepsilon^2}\right)$; this has generator

$$\hat{\mathcal{L}}_\varepsilon f(\alpha) = \frac{n}{\varepsilon^2}\eta(\alpha)I_{\mathrm{eq}}(\alpha)\int_{\alpha_{\min}}^{\alpha_{\max}} K_\varepsilon(\alpha, \alpha' - \alpha)s(\alpha,\alpha')\big(f(\alpha') - f(\alpha)\big)\, d\alpha'.$$

In Section 8.3, we prove

Proposition 10. *As $\varepsilon \to 0$, $\hat{A}_\varepsilon(t)$ converges to a limiting diffusion $\hat{A}(t)$ with advective coefficient*

$$\mu(\alpha) = n\eta(\alpha)I_{eq}(\alpha)\nu(\alpha)\frac{\partial}{\partial\alpha'}s(\alpha,\alpha) = \frac{\eta(\alpha)\nu(\alpha)}{2}\left(nI_{eq}(\alpha)\frac{R_0'(\alpha)}{R_0(\alpha)} - \frac{\beta'(\alpha)}{\beta(\alpha)}\right) \tag{S.29}$$

and diffusion coefficient

$$\sigma^2(\alpha) = n\eta(\alpha)I_{eq}(\alpha)\nu(\alpha)s(\alpha,\alpha) = \eta(\alpha)\nu(\alpha). \tag{S.30}$$

and generator

$$\hat{\mathcal{L}}f(\alpha) = \mu(\alpha)f'(\alpha) + \frac{1}{2}\sigma^2(\alpha)f''(\alpha).$$

for $f \in C^2[\alpha_{\min}, \alpha_{max}]$ such that $f'(\alpha_{min}) = f'(\alpha_{max}) = 0$.

The process $\hat{A}(t)$ is our canonical diffusion.

Remark 5. The condition that $f'(\alpha_{\min}) = f'(\alpha_{\max}) = 0$ corresponds to a zero-flux (reflecting) boundary condition at $\alpha_{\min}$ and $\alpha_{\max}$ for the corresponding forward equation [16]:

$$\begin{aligned}
\int_{\alpha_{\min}}^{\alpha_{\max}} g(\alpha)\hat{\mathcal{L}}f(\alpha)\,d\alpha &= \int_{\alpha_{\min}}^{\alpha_{\max}} g(\alpha)\mu(\alpha)f'(\alpha)\,d\alpha + \frac{1}{2}\int_{\alpha_{\min}}^{\alpha_{\max}} g(\alpha)\sigma^2(\alpha)f''(\alpha)\,d\alpha \\
&= \Big(g(\alpha_{\min})\mu(\alpha_{\min}) - \big(g(\alpha_{\min})\sigma^2(\alpha_{\min})\big)'\Big) f(\alpha_{\min}) - \Big(g(\alpha_{\max})\mu(\alpha_{\max}) - \big(g(\alpha_{\max})\sigma^2(\alpha_{\max})\big)'\Big) f(\alpha_{\max}) \\
&\quad - g(\alpha_{\min})\sigma^2(\alpha_{\min})f(\alpha_{\min})f'(\alpha_{\min}) + g(\alpha_{\max})\sigma^2(\alpha_{\max})f(\alpha_{\max})f'(\alpha_{\max}) \\
&\quad - \int_{\alpha_{\min}}^{\alpha_{\max}} \big(g(\alpha)\mu(\alpha)\big)' f(\alpha)\,d\alpha + \frac{1}{2}\int_{\alpha_{\min}}^{\alpha_{\max}} \big(g(\alpha)\sigma^2(\alpha)\big)'' f(\alpha)\,d\alpha,
\end{aligned}$$

which equals $\int_{\alpha_{\min}}^{\alpha_{\max}} \hat{\mathcal{L}}^* g(\alpha)f(\alpha)\,d\alpha$ for arbitrary f such that $f'(\alpha_{\min}) = f'(\alpha_{\max}) = 0$ if and only if the flux $g(\alpha)\mu(\alpha) - \big(g(\alpha)\sigma^2(\alpha)\big)'$ vanishes at $\alpha_{\min}$ and $\alpha_{\max}$.

6.1 Stationary Distribution

Using (S.29) and (S.30), we may compute the stationary distribution ψ of $\hat{A}(t)$; this stationary distribution describes the long-term behaviour of the virulence, after any "memory" of the initial state has been lost. Given any subset $\mathcal{A} \subset (\alpha_{\min}, \alpha_{\max})$, $\psi(\mathcal{A})$ gives the proportion of time that the virulence is in the set $\mathcal{A}$, or equivalently, the probability that at some random sampling time t, the virulence takes a value in $\mathcal{A}$. ψ is characterised by the relation

$$\int_{\alpha_{\min}}^{\alpha_{\max}} \hat{\mathcal{L}}f(\alpha)\psi(d\alpha) = 0.$$

In particular, if $\psi(d\alpha)$ has a density, which, in a slight abuse of notation, we write as $\psi(\alpha)$,

$$\hat{\mathcal{L}}^*\psi = 0,$$

where $\hat{\mathcal{L}}^*$, defined by

$$\hat{\mathcal{L}}^* f(\alpha) = -\frac{d}{d\alpha}\left[\mu(\alpha)f(\alpha)\right] + \frac{1}{2}\frac{d^2}{d\alpha^2}\left[\sigma^2(\alpha)f(\alpha)\right],$$

is the adjoint operator to $\hat{\mathcal{L}}$ (and thus,

$$\frac{d}{dt}f(\alpha,t) = \hat{\mathcal{L}}^* f(\alpha,t)$$

is the Fokker-Planck equation for the probability density of $\hat{A}(t)$, $f(\alpha,t)$).

Thus,

$$\psi(\alpha) = \frac{1}{Z}\frac{1}{\sigma^2(\alpha)}e^{\int \frac{2\mu(\alpha)}{\sigma^2(\alpha)}\,d\alpha}\,d\alpha \tag{S.31}$$

where $Z = \int_{\alpha_{\min}}^{\alpha_{\max}} \frac{1}{\sigma^2(\alpha)}e^{\int \frac{2\mu(\alpha)}{\sigma^2(\alpha)}\,d\alpha}\,d\alpha$ is a normalising constant.

From the previous, we have

$$\frac{2\mu(\alpha)}{\sigma^2(\alpha)} = nI_{\text{eq}}(\alpha)\frac{R_0'(\alpha)}{R_0(\alpha)} - \frac{\beta'(\alpha)}{\beta(\alpha)}. \tag{S.32}$$

Unfortunately, we can only compute its integral analytically in the case of a "flat landscape", when $R_0(\alpha) \equiv R_0$, independently of α. In this case, we have $\beta(\alpha) = R_0(\delta+\alpha+\gamma)$, $\beta'(\alpha) = R_0$, and

$$\frac{2\mu(\alpha)}{\sigma^2(\alpha)} = -\frac{\beta'(\alpha)}{\beta(\alpha)} = -\frac{1}{\delta+\alpha+\gamma}$$

which has integral $-\ln(\delta+\alpha+\gamma)$, so that

$$\frac{1}{\sigma^2(\alpha)} e^{\int \frac{2\mu(\alpha)}{\sigma^2(\alpha)}\, d\alpha}\, d\alpha = \frac{1}{\sigma^2(\alpha)} \frac{1}{\delta+\alpha+\gamma}.$$

In particular, in the case when $\eta(\alpha)$ and $\nu(\alpha)$ (and thus $\sigma^2(\alpha)$) are constants independent of α, then we can integrate this to obtain Z and thus a closed expression for the stationary distribution:

$$\psi(\alpha) = \frac{1}{\ln\left(\frac{\delta+\alpha_{\max}+\gamma}{\delta+\alpha_{\min}+\gamma}\right)} \frac{1}{(\delta+\alpha+\gamma)}. \tag{S.33}$$

Because this is a zero-flux solution, $\alpha_{\min}$ and $\alpha_{\max}$ may be chosen arbitrarily (we have a zero-flux boundary condition). We take $\alpha_{\min} = 0$; $\alpha_{\max}$ must be finite to ensure that Z is finite.

In the next section we will show how one may obtain an analytical approximation in the large n limit.

6.2 Asymptotic Approximation to Stationary Distribution

The stationary distribution (S.31) lends itself to an approximation by Laplace's method (see *e.g.,* [12]), which tells us that if $\phi(x)$ is a twice differentiable function with a unique local maximum attained at $x_0 \in (a,b)$, and $g(x)$ is continuous, then

$$\int_a^b g(x)e^{n\phi(x)}\, dx = \sqrt{-\frac{2\pi}{n\phi''(x_0)}} g(x_0)e^{n\phi(x_0)} \left(1 + \mathcal{O}\left(\tfrac{1}{n}\right)\right).$$

(To order $\frac{1}{n}$, one has

$$\int_a^b g(x)e^{n\phi(x)}\, dx = \sqrt{-\frac{2\pi}{n\phi''(x_0)}} e^{n\phi(x_0)} \Bigg(g(x_0) + \frac{1}{n}\left(\frac{1}{2}\frac{g''(x_0)}{\phi''(x_0)} + \frac{1}{8}\frac{g^{(4)}(x_0)}{(\phi''(x_0))^2} + \frac{1}{2}\frac{g'(x_0)\phi^{(3)}(x_0)}{(\phi''(x_0))^2} + \frac{5}{24}\frac{g(x_0)(\phi^{(3)}(x_0))^2}{(\phi''(x_0))^3}\right) + \mathcal{O}\left(\tfrac{1}{n^2}\right)\Bigg); \tag{S.34}$$

see *e.g.,* [4]).

We can apply this to our stationary distribution by first observing from (S.32) that

$$\frac{1}{\sigma^2(\alpha)} e^{\int \frac{2\mu(\alpha)}{\sigma^2(\alpha)}\, d\alpha} = g(\alpha) e^{n\phi(\alpha)}$$

for

$$\phi(\alpha) = \int I_{\text{eq}}(\alpha) \frac{R_0'(\alpha)}{R_0(\alpha)}\, d\alpha \tag{S.35}$$

and

$$g(\alpha) = \frac{1}{\sigma^2(\alpha)\beta(\alpha)},$$

and we can thus apply Laplace's formula to compute Z.

To find our α_0, we note that by assumption, we have chosen α_{min} and α_{max} so that $\frac{\lambda(R_0(\alpha)-1)}{\beta(\alpha)-\alpha} > 0$ for all $\alpha \in (\alpha_{\text{min}}, \alpha_{\text{max}})$. Thus, all values of α_0 such that $\phi'(\alpha_0) = 0$ satisfy $R_0'(\alpha_0) = 0$.

On the other hand, we recall that $R_0(\alpha) = \frac{\beta(\alpha)}{\delta+\alpha+\gamma}$, so that

$$R_0'(\alpha) = \frac{\beta'(\alpha)}{\delta+\alpha+\gamma} - \frac{\beta(\alpha)}{(\delta+\alpha+\gamma)^2}, \tag{S.36}$$

and thus also $R_0(\alpha_0) = \beta'(\alpha_0)$.

We next observe that

$$R''(\alpha_0) = \frac{\beta''(\alpha_0)}{\delta+\alpha_0+\gamma}. \tag{S.37}$$

which depends on the choice of tradeoff function $\beta(\alpha)$. We note briefly that this is a local maximum if and only if $\beta''(\alpha_0) < 0$. In particular, if we assume that $R_0(\alpha)$ has a unique global maximum, then it must occur at α_0. We will henceforth make this assumption (*n.b.,* we don't have to assume this in general, but in applying Laplace's method, we require that α_0 be a global maximum; more generally we could partition $(\alpha_{\text{min}}, \alpha_{\text{max}})$ into intervals containing a single local maxima).

We then have

$$\phi''(\alpha) = I_{\text{eq}}'(\alpha)\frac{R_0'(\alpha)}{R_0(\alpha)} + I_{\text{eq}}(\alpha)\left(\frac{R_0''(\alpha)}{R_0(\alpha)} - \left(\frac{R_0'(\alpha)}{R_0(\alpha)}\right)^2\right)$$

so that

$$\phi''(\alpha_0) = I_{\text{eq}}(\alpha_0)\frac{R_0''(\alpha_0)}{R_0(\alpha_0)}.$$

Thus,

$$Z = \int_{\alpha_{\text{min}}}^{\alpha_{\text{max}}} \frac{1}{\sigma^2(\alpha)} e^{\int \frac{2\mu(\alpha)}{\sigma^2(\alpha)}\, d\alpha}\, d\alpha = \sqrt{\frac{2\pi}{nI_{\text{eq}}(\alpha_0)\frac{|R_0''(\alpha_0)|}{R_0(\alpha_0)}}}\, e^{n\phi(\alpha_0)} \left(\frac{1}{\sigma^2(\alpha_0)\beta(\alpha_0)} + \mathcal{O}\left(\tfrac{1}{n}\right)\right).$$

We then have

$$\frac{1}{Z}\frac{1}{\sigma^2(\alpha)}e^{\int \frac{2\mu(\alpha)}{\sigma^2(\alpha)}\,d\alpha} = \frac{\sigma^2(\alpha_0)\beta(\alpha_0)}{\sigma^2(\alpha)\beta(\alpha)}\frac{1}{\sqrt{\frac{2\pi}{nI_{\text{eq}}(\alpha_0)\frac{\left|R_0''(\alpha_0)\right|}{R_0(\alpha_0)}}}}e^{n(\phi(\alpha)-\phi(\alpha_0))}\left(1+\mathcal{O}\left(\tfrac{1}{n}\right)\right).$$

Next, recalling that $\phi'(\alpha_0)=0$, a Taylor expansion gives

$$n(\phi(\alpha)-\phi(\alpha_0)) = \frac{1}{2}n\left(\phi''(\alpha_0)(\alpha-\alpha_0)^2 + \mathcal{O}\big((\alpha-\alpha_0)^3\big)\right).$$

Now, for α close to α_0, $(\alpha-\alpha_0)^3$ will be quite small, so we can locally approximate our full stationary distribution by a process that is almost a Gaussian with mean α_0 and variance

$$\frac{1}{nI_{\text{eq}}(\alpha_0)\frac{\left|R_0''(\alpha_0)\right|}{R_0(\alpha_0)}},$$

except for a pre-factor of $\frac{\sigma^2(\alpha_0)\beta(\alpha_0)}{\sigma^2(\alpha)\beta(\alpha)}$, which skews the distribution:

$$\begin{aligned}\frac{1}{Z}\frac{1}{\sigma^2(\alpha)}e^{\int \frac{2\mu(\alpha)}{\sigma^2(\alpha)}\,d\alpha} &\approx \frac{\sigma^2(\alpha_0)\beta(\alpha_0)}{\sigma^2(\alpha)\beta(\alpha)}\frac{1}{\sqrt{\frac{2\pi}{nI_{\text{eq}}(\alpha_0)\frac{\left|R_0''(\alpha_0)\right|}{R_0(\alpha_0)}}}}e^{-\frac{1}{2}nI_{\text{eq}}(\alpha_0)\frac{\left|R_0''(\alpha_0)\right|}{R_0(\alpha_0)}(\alpha-\alpha_0)^2} \\ &= \frac{\eta(\alpha_0)}{\eta(\alpha)}\frac{\nu(\alpha_0)}{\nu(\alpha)}\frac{\beta(\alpha_0)}{\beta(\alpha)}\;\frac{1}{\sqrt{\frac{2\pi}{nI_{\text{eq}}(\alpha_0)\frac{\left|R_0''(\alpha_0)\right|}{R_0(\alpha_0)}}}}e^{-\frac{1}{2}nI_{\text{eq}}(\alpha_0)\frac{\left|R_0''(\alpha_0)\right|}{R_0(\alpha_0)}(\alpha-\alpha_0)^2}.\end{aligned}$$

Again assuming that $\eta(\alpha)$ and $\nu(\alpha)$ are constants independent of α, this simplifies to

$$\psi_{\text{approx}}(\alpha) = \frac{\beta(\alpha_0)}{\beta(\alpha)}\;\frac{1}{\sqrt{\frac{2\pi}{nI_{\text{eq}}(\alpha_0)\frac{\left|R_0''(\alpha_0)\right|}{R_0(\alpha_0)}}}}e^{-\frac{1}{2}nI_{\text{eq}}(\alpha_0)\frac{\left|R_0''(\alpha_0)\right|}{R_0(\alpha_0)}(\alpha-\alpha_0)^2}. \tag{S.38}$$

Remark 6. Applying (S.34) with $f(\alpha)g(\alpha)$ in place of $g(\alpha)$ for an arbitrary differentiable function $f(\alpha)$, we may estimate the error in integrating $f(\alpha)$ versus the true and approximate densities for the stationary distribution:

$$\begin{aligned}&\left|\frac{1}{Z}\int_{\alpha_{\min}}^{\alpha_{\max}} f(\alpha)g(\alpha)e^{-n\int\phi(\alpha)\,d\alpha}\,d\alpha - \frac{1}{\sqrt{\frac{2\pi}{n|\phi''(\alpha_0)|}}g(\alpha_0)}\int_{\alpha_{\min}}^{\alpha_{\max}} f(\alpha)g(\alpha)e^{-n\frac{\phi''(\alpha_0)}{2}(\alpha-\alpha_0)^2}\,d\alpha\right| \\ &\qquad = \frac{1}{n}\left|\frac{1}{2}\frac{f'(\alpha_0)\phi'''(\alpha_0)}{(\phi''(\alpha_0))^2} - \frac{1}{2}\frac{f(\alpha_0)g''(\alpha_0)}{g(\alpha_0)|\phi''(\alpha_0)|}\right| + \mathcal{O}\left(\tfrac{1}{n^2}\right)\end{aligned}$$

From this, see that in the bounded Lipschitz metric on probability measures, the difference between

the true stationary approximation $\psi(d\alpha)$ and the Laplace approximation $\psi_{\text{approx}}(d\alpha)$ satisfies

$$\lim_{n\to\infty} d_{\text{BL}}(\psi, \psi_{\text{approx}}) \leq \frac{1}{2}\left|\frac{\phi'''(\alpha_0)}{(\phi''(\alpha_0))^2}\right| + \frac{1}{2}\left|\frac{g''(\alpha_0)}{g(\alpha_0)|\phi''(\alpha_0)|}\right|.$$

Unfortunately, we cannot similarly bound the total variation distance: for any $M > 0$ the function $f(\alpha) := e^{-M(\alpha-\alpha_{\min})^2}$ satisfies

$$\sup_{\alpha\in[\alpha_{\min},\alpha_{\max}]} |f(\alpha)| \leq 1,$$

but, since

$$\sup_{\alpha\in[\alpha_{\min},\alpha_{\max}]} |f(\alpha)| = \sqrt{2M}e^{-\frac{1}{2}},$$

the bound

$$\frac{1}{n}\left|\frac{1}{2}\frac{f'(\alpha_0)\phi'''(\alpha_0)}{(\phi''(\alpha_0))^2} - \frac{1}{2}\frac{f(\alpha_0)g''(\alpha_0)}{g(\alpha_0)|\phi''(\alpha_0)|}\right|$$

may be made arbitrarily large.

6.3 Mean & Mode of the Stationary Distribution

We observe that, whilst the stationary distribution is closely related to a Gaussian centred at α_0, the value of the virulence that maximises $R_0(\alpha)$, the full stationary distribution does not have mean α_0, and α_0 is not the most probable value of the virulence.

6.3.1 Estimating the Mean

Applying Laplace's method with $g(\alpha)$ replaced by $\alpha g(\alpha)$ allows us to estimate the mean of the stationary distribution; to lowest order, we have

$$\int_{\alpha_{\min}}^{\alpha^{max}} \alpha g(\alpha)e^{n\phi(\alpha)}\, d\alpha = \sqrt{-\frac{2\pi}{n\phi''(\alpha_0)}}\alpha_0 g(\alpha_0)e^{n\phi(\alpha_0)}\left(1 + \mathcal{O}\left(\tfrac{1}{n}\right)\right),$$

so that, normalising by our prior estimate of Z, we find that the mean is α_0 to order $\mathcal{O}\left(\frac{1}{n}\right)$. To observe the effects of a finite population size, we can the use higher order corrections to Laplace's method to obtain the $\mathcal{O}\left(\frac{1}{n}\right)$ terms in both the integral above and in Z; we omit the calculations, but remark that the mean can then be shown to be

$$\alpha_0 - \frac{1}{n}\left(\frac{g'(\alpha_0)}{g(\alpha_0)\phi''(\alpha_0)} - \frac{\phi'''(\alpha_0)}{2(\phi''(\alpha_0))^2}\right) + o\left(\tfrac{1}{n}\right)$$
$$= \alpha_0 + \frac{1}{nI_{\text{eq}}(\alpha_0)\frac{|R_0''(\alpha_0)|}{R_0(\alpha_0)}}\left(\frac{g'(\alpha_0)}{g(\alpha_0)} - \frac{\phi'''(\alpha_0)}{2\phi''(\alpha_0)}\right) + o\left(\tfrac{1}{n}\right)$$

where

$$\begin{aligned}\frac{g'(\alpha_0)}{g(\alpha_0)} &= -\frac{\eta'(\alpha_0)}{\eta(\alpha_0)} - \frac{\nu'(\alpha_0)}{\nu(\alpha_0)} - \frac{\beta'(\alpha_0)}{\beta(\alpha_0)} \\ &= -\frac{\eta'(\alpha_0)}{\eta(\alpha_0)} - \frac{\nu'(\alpha_0)}{\nu(\alpha_0)} - \frac{1}{\delta+\alpha_0+\gamma},\end{aligned}$$

which simplifies to $-\frac{1}{\delta+\alpha_0+\gamma}$ in the case when η and ν are independent of α, and

$$\frac{\phi'''(\alpha_0)}{\phi''(\alpha_0)} = \frac{R_0'''(\alpha_0)}{R_0''(\alpha_0)} - \frac{\beta'(\alpha_0)-1}{\beta(\alpha_0)-\alpha_0} = \frac{I_{\text{eq}}'(\alpha_0)}{I_{\text{eq}}(\alpha_0)} - \frac{R_0'''(\alpha_0)}{|R_0''(\alpha_0)|}.$$

We note that this order $\mathcal{O}\left(\frac{1}{n}\right)$ term is proportional to the variance of the best-fit Gaussian of the previous section. Note also that

$$I_{\text{eq}}'(\alpha) = \frac{\lambda R_0'(\alpha)}{\beta(\alpha)-\alpha} - \frac{\lambda R_0(\alpha)(\beta'(\alpha)-1)}{(\beta(\alpha)-\alpha)^2},$$

so that $I_{\text{eq}}'(\alpha_0) = -\frac{\lambda R_0(\alpha_0)(R_0(\alpha_0)-1)}{(\beta(\alpha_0)-\alpha_0)^2} < 0$.

One may similarly show that the variance and skewness of the stationary distribution are

$$\frac{1}{n}\frac{1}{\phi''(\alpha_0)} + o\left(\tfrac{1}{n}\right) = \frac{1}{nI_{\text{eq}}(\alpha_0)\frac{|R_0''(\alpha_0)|}{R_0(\alpha_0)}} + o\left(\tfrac{1}{n}\right)$$

and

$$\frac{1}{n}\frac{3g'(\alpha_0)}{g(\alpha_0)(\phi''(\alpha_0))^2} + o\left(\tfrac{1}{n}\right)$$

respectively. We note that if $\eta'(\alpha_0) \geq 0$ and $\nu'(\alpha_0) \geq 0$ (for example, if both rates are independent of α), then the stationary distribution has negative skew.

6.3.2 Estimating the Mode

We begin by observing the density function of the stationary distribution (S.31) may be written as

$$e^{\int \frac{2\mu(\alpha)}{\sigma^2(\alpha)}\,d\alpha - \ln\frac{\sigma^2(\alpha)}{Z}}\,d\alpha$$

and thus has its maximum where

$$-\frac{2\mu(\alpha)}{\sigma^2(\alpha)} = \frac{d}{d\alpha}\ln\frac{\sigma^2(\alpha)}{Z},$$

or equivalently, for the value of $\alpha^\star$ such that

$$n\phi'(\alpha^\star) = -\frac{g'(\alpha^\star)}{g(\alpha^\star)}$$

where $\phi(\alpha)$ is given by (S.35).

When $\sigma^2(\alpha)$ is constant (*i.e.,* the variance of the mutation kernel is independent of the resident variance) then this reduces to

$$\frac{2\mu(\alpha^\star)}{\sigma^2} = 0,$$

and thus $\mu(\alpha^\star) = 0$. Recalling (S.29), this tells us that

$$\frac{1}{\delta + \alpha^\star + \gamma} - \left(1 - \frac{1}{nI_{\text{eq}}(\alpha^\star)}\right) \frac{\beta'(\alpha^\star)}{\beta(\alpha^\star)} = 0,$$

and thus

$$\beta'(\alpha^\star) = R_0(\alpha^\star) \left(1 + \frac{1}{nI_{\text{eq}}(\alpha^\star) - 1}\right). \tag{S.39}$$

On the other hand, (S.36) tells us that

$$\frac{R_0'(\alpha)}{R_0(\alpha)} = \frac{\beta'(\alpha)}{\beta(\alpha)} - \frac{1}{\delta + \alpha + \gamma},$$

so that

$$\frac{R_0'(\alpha^\star)}{R_0(\alpha^\star)} = \frac{1}{nI_{\text{eq}}(\alpha^\star)} \frac{\beta'(\alpha^\star)}{\beta(\alpha^\star)} > 0,$$

since $\beta(\alpha)$ is increasing. In particular, since $R_0'(\alpha)$ is maximized at α_0, we see immediately that $\alpha^\star < \alpha_0$.

Even in this special case, we cannot solve for $\alpha^\star$ exactly. Instead we will seek a perturbative solution to

$$\phi'(\alpha^\star) = -\frac{1}{n} \frac{g'(\alpha^\star)}{g(\alpha^\star)} \tag{S.40}$$

in the general case. We already know that $\phi'(\alpha_0) = 0$; we thus seek a solution of the form

$$\alpha^\star = \alpha_0 + \sum_{i=1}^{\infty} \frac{\alpha_i}{n^i}.$$

Substituting this into (S.40) and Taylor expanding right and left, we find that

$$\phi'(\alpha_0) + \frac{1}{n}\phi''(\alpha_0)\alpha_1 = -\frac{1}{n} \frac{g'(\alpha_0)}{g(\alpha_0)} + o\big(\tfrac{1}{n}\big)$$

i.e., that

$$\alpha^\star = \alpha_0 - \frac{1}{n} \frac{g'(\alpha_0)}{g(\alpha_0)\phi''(\alpha_0)} + o\big(\tfrac{1}{n}\big) = \alpha_0 + \frac{1}{nI_{\text{eq}}(\alpha_0) \frac{|R_0''(\alpha_0)|}{R_0(\alpha_0)}} \frac{g'(\alpha_0)}{g(\alpha_0)} + o\big(\tfrac{1}{n}\big)$$

which may be expanded using the expression for $\frac{g'(\alpha_0)}{g(\alpha_0)}$ given in the previous sections. In particular,

when when $\eta(\alpha)$ and $\nu(\alpha)$ (and thus $\sigma^2(\alpha)$) are constants independent of α, we have that

$$\alpha^\star = \alpha_0 - \frac{1}{nI_{\text{eq}}(\alpha_0)\frac{|R_0''(\alpha_0)|}{R_0(\alpha_0)}} \frac{1}{\delta + \alpha_0 + \gamma} + o\left(\tfrac{1}{n}\right), \tag{S.41}$$

so that to first order, the modal virulence is the virulence maximizing $R_0(\alpha)$ less the product of the variance of the best-fit Gaussian and the expected infectious period when the virulence is α_0.

7 Some Remarks Regarding Simulations

For all simulations, we simulate the Markov process described in Table 1.

For Figure 2, we consider a single individual infected with the mutant strain with the initial number of susceptibles, infecteds and total individuals set equal to the closest integers to nS_{eq}, nI_{eq} and nN_{eq}, where (S_{eq}, I_{eq}, N_{eq}) is the (deterministic) endemic equilibrium with only the resident strain present. No further mutations are allowed to occur. For each value of s and σ, 10^6 simulations were allowed to continue until fixation of the mutant or resident, and we plot the fraction in which the mutant fixed.

For Figures 3, 4 & 5, following Section 6, we impose a tradeoff between β and α, here taking

$$\beta(\alpha) = (\delta + \alpha + \gamma)(R_{0,\max} - w(\alpha - \alpha_0)^2).$$

The simulations were started with $nN_{eq}(\alpha_0)$ individuals, $nI_{eq}(\alpha_0)$ infected with a resident strain with virulence α_0 – the virulence maximizing $R_0(\alpha)$ – and $nS_{eq}(\alpha_0)$ susceptibles (as previously, $(S_{eq}(\alpha_0), N_{eq}(\alpha_0), N_{eq}(\alpha_0))$ is the endemic equilibrium for the deterministic limit with a resident strain of virulence α_0.) With probability μ per time-step, an individual was selected uniformly at random from the infected population, and the virulence of the mutant pathogen was selected according to the kernel

$$K(\alpha_m, \alpha) = \begin{cases} \frac{1}{2} & \text{if } \alpha_m - \alpha = \pm\Delta\alpha, \text{ and} \\ 0 & \text{otherwise,} \end{cases}$$

for $\Delta\alpha = \frac{6}{100}$, $\alpha_{\min} = 0$, and $\alpha_{\max} = 6$. There are thus 100 discrete values for the virulence (*n.b.*, by assumption, for $\alpha = \alpha_{\min}$, mutations $\Delta\alpha$ occur with probability $\frac{1}{2}$, whereas the process remains at $\alpha_{\min}$ with probability $\frac{1}{2}$, and similarly for $\alpha = \alpha_{\max}$) which are the bins of the histogram in Figures 3, 4 & 5.

In [7], it is shown that μ has to be of the order of $\mathcal{O}\left(\frac{1}{n \ln n}\right)$ or smaller to ensure that mutations occur sufficiently rarely that little or no clonal interference occurs, and such that the resident population is in a small neighbourhood of the equilibrium of the deterministic limit when a mutation occurs. In Figures 3& 4, we take $n = 200$, so $n \ln n \approx 1060$ and take $\mu = \frac{1}{1000}$ to be in the appropriate range. A small amount of clonal interference was observed, but it did not contribute significantly to the histogram. For selection, taking $w = 0$ forces all strains to have equal R_0, ensuring weak selection, whereas for $w > 0$,

$$|R_0(\alpha) - R_0(\alpha')| \approx |R_0'(\alpha)(\alpha - \alpha')| = 2wR_{0,\max}|\alpha_0 - \alpha||\alpha - \alpha'| \leq 1.44w,$$

so selection is always of order $\mathcal{O}\left(\frac{1}{n}\right)$ for $w = 0.01$, whereas when $w = 0.1$, one could see strong selection when $|\alpha_0 - \alpha|$ is large. Despite this, our analytical results continue to accurately predict the stationary distribution in Figures 4C & 5, where selection need not be weak (α is far from α_0).

8 Some Rigorous Demonstrations

8.1 Proof of Proposition 2

Substituting f with Π_i in Itô's formula (S.8) for jump processes yields

$$P_i^{(n)}(t) = P_i^{(n)}(0) + \int_0^t \sum_{j=1}^{d} \frac{\partial \Pi_i}{\partial x_j}(\bar{\boldsymbol{I}}^{(n)}(s)) F_j^{(n)}(\bar{\boldsymbol{E}}^{(n)}(s)) + \frac{1}{2} \sum_{j,k=1}^{d} a_{jk}^{(n)}(\bar{\boldsymbol{E}}^{(n)}(s)) \frac{\partial \Pi_i}{\partial x_j \partial x_k}(\bar{\boldsymbol{I}}^{(n)}(s))\, ds$$
$$+ \frac{1}{n} \int_0^t \sum_{j=1}^{d} \frac{\partial \Pi_i}{\partial x_j}(\bar{\boldsymbol{I}}^{(n)}(s))\, dM_j^{(n)}(s) + \varepsilon_i^{(n)}(t), \quad \text{(S.42)}$$

where $\varepsilon_i^{(n)}(t)$ can be expressed thanks to Equation (S.9) as

$$\varepsilon_i^{(n)}(t) = \sum_{s<t} \Pi_i(\bar{\boldsymbol{I}}^{(n)}(s)) - \Pi_i(\bar{\boldsymbol{I}}^{(n)}(s-)) - \sum_{j=1}^{d} \frac{\partial \Pi_i}{\partial x_j}(\bar{\boldsymbol{I}}^{(n)}(s-)) \Delta \bar{I}_j^{(n)}(s)$$
$$- \frac{1}{2} \sum_{j,k=1}^{d} \frac{\partial \Pi_i}{\partial x_j \partial x_k}(\bar{\boldsymbol{I}}^{(n)}(s-)) \Delta \bar{I}_j^{(n)}(s) \Delta \bar{I}_k^{(n)}(s).$$

Now some elementary computations yield

$$\frac{\partial \Pi_i}{\partial x_j} = \frac{1}{\sum_{l=1}^{d} x_l} \left(\mathbb{1}_{\{i=j\}} - \frac{x_i}{\sum_{l=1}^{d} x_l} \right) \quad \text{and} \quad \frac{\partial \Pi_i}{\partial x_j \partial x_k} = -\frac{1}{\left(\sum_{l=1}^{d} x_l\right)^2} \left(\mathbb{1}_{\{i=j\}} + \mathbb{1}_{\{i=k\}} - 2\frac{x_i}{\sum_{l=1}^{d} x_l} \right),$$

where $\mathbb{1}_{\{i=j\}}$ is equal to 1 if $i = j$ and 0 otherwise. Substituting these and (S.6) into (S.42) yields after some simplification Equation (S.10) in Proposition 2.

Now it remains to prove that $n^2 \varepsilon_i^{(n)}(t)$ is uniformly bounded with high probability. Taylor's theorem tells us that

$$\Pi_i(\bar{\boldsymbol{I}}^{(n)}(s)) - \Pi_i(\bar{\boldsymbol{I}}^{(n)}(s-)) - \sum_{j=1}^{d} \frac{\partial \Pi_i}{\partial x_j}(\bar{\boldsymbol{I}}^{(n)}(s-)) \Delta \bar{I}_j^{(n)}(s) - \frac{1}{2} \sum_{j,k=1}^{d} \frac{\partial \Pi_i}{\partial x_j \partial x_k}(\bar{\boldsymbol{I}}^{(n)}(s-)) \Delta \bar{I}_j^{(n)}(s) \Delta \bar{I}_k^{(n)}(s)$$
$$= \sum_{j,k=1}^{d} g_{ij}(\bar{\boldsymbol{I}}^{(n)}(s), \bar{\boldsymbol{I}}^{(n)}(s-)) \Delta \bar{I}_j^{(n)}(s) \Delta \bar{I}_k^{(n)}(s),$$

where the functions $g_{ij}(\boldsymbol{x}, \boldsymbol{y})$ satisfy

$$\lim_{\boldsymbol{x}\to\boldsymbol{y}} g_{ij}(\boldsymbol{x}, \boldsymbol{y}) = 0$$

uniformly on compact sets.

Now, recalling

$$\begin{aligned}\bar{I}_i^{(n)}(t) = \bar{I}_i^{(n)}(0) + \frac{1}{n} P_{-\boldsymbol{e}_0+\boldsymbol{e}_i}\left(n\int_0^t \frac{\beta_i^{(n)}\bar{S}^{(n)}(s)\bar{I}_i^{(n)}(s)}{\bar{N}^{(n)}(s)}\,ds\right) \\ - \frac{1}{n} P_{-\boldsymbol{e}_i-\boldsymbol{e}_{d+1}}\left(n\int_0^t (\delta^{(n)}+\alpha_i^{(n)})\bar{I}_i^{(n)}(s)\,ds\right) - \frac{1}{n}P_{-\boldsymbol{e}_i}\left(n\int_0^t \gamma_i^{(n)}\bar{I}_i^{(n)}(s)\,ds\right),\end{aligned}$$

we see that $\Delta\bar{I}_i^{(n)}(s)$ is non-zero only at the jump-times of the Poisson processes and are always of magnitude $\frac{1}{n}$. In particular, since $f_i(\boldsymbol{x})$ is smooth outside of a neighbourhood of $\mathbf{0}$, we can conclude that g_{ij} is bounded above by a constant multiple of $\|\boldsymbol{x}-\boldsymbol{y}\|$; this allows us to conclude that $|g_{ij}(\bar{\boldsymbol{I}}^{(n)}(s), \bar{\boldsymbol{I}}^{(n)}(s-))| \leq \frac{C}{n}$ and

$$|\varepsilon_i^{(n)}(t)| \leq \frac{C}{n}\sum_{s<t}\sum_{j,k=1}^{d} |\Delta\bar{I}_j^{(n)}(s)||\Delta\bar{I}_k^{(n)}(s)|.$$

Further, $|\Delta\bar{I}_j^{(n)}(s)||\Delta\bar{I}_k^{(n)}(s)|$ is non-zero if some pair of processes in $P_{-\boldsymbol{e}_0+\boldsymbol{e}_j}, P_{-\boldsymbol{e}_j-\boldsymbol{e}_{d+1}}, P_{-\boldsymbol{e}_j}$, and $P_{-\boldsymbol{e}_0+\boldsymbol{e}_k}, P_{-\boldsymbol{e}_k-\boldsymbol{e}_{d+1}}, P_{-\boldsymbol{e}_k}$ jump simultaneously; if $j \neq k$, the Poisson processes are independent, and probability of such an event in an interval $[t, t+\Delta t)$ is $\mathcal{O}(\Delta t^2)$, and thus tends to 0 if as $\Delta t \to 0$ *i.e.*, $|\Delta\bar{I}_j^{(n)}(s)||\Delta\bar{I}_k^{(n)}(s)| \neq 0$ if and only if $j \neq k$. Moreover, the processes $P_{-\boldsymbol{e}_0+\boldsymbol{e}_j}$, $P_{-\boldsymbol{e}_0-\boldsymbol{e}_{d+1}}$ and $P_{-\boldsymbol{e}_j}$ are also independent, and thus cannot jump simultaneously, so that $|\Delta\bar{I}_j^{(n)}(s)||\Delta\bar{I}_k^{(n)}(s)| \neq 0$ (and is thus equal to $\frac{1}{n^2}$) at exactly the jump times of these Poisson processes; *i.e.*,

$$\begin{aligned}\sum_{s<t}\sum_{j,k=1}^{d} |\Delta\bar{I}_j^{(n)}(s)||\Delta\bar{I}_k^{(n)}(s)| = \frac{1}{n^2}P_{-\boldsymbol{e}_0+\boldsymbol{e}_i}\left(n\int_0^t \frac{\beta_i^{(n)}\bar{S}^{(n)}(s)\bar{I}_i^{(n)}(s)}{\bar{N}^{(n)}(s)}\,ds\right) \\ + \frac{1}{n^2}P_{-\boldsymbol{e}_i-\boldsymbol{e}_{d+1}}\left(n\int_0^t (\delta^{(n)}+\alpha_i^{(n)})\bar{I}_i^{(n)}(s)\,ds\right) \\ + \frac{1}{n^2}P_{-\boldsymbol{e}_i}\left(n\int_0^t \gamma_i^{(n)}\bar{I}_i^{(n)}(s)\,ds\right)\end{aligned}$$

We seek an upper bound on this quantity. To that end, we begin by observing that $\bar{S}^{(n)}(t)$ and each $\bar{I}_i^{(n)}(t)$ is bounded above by $\bar{N}^{(n)}(t)$, and that

$$\bar{N}^{(n)}(t) \leq \bar{N}^{(n)}(0) + \frac{1}{n}P_{\boldsymbol{e}_0+\boldsymbol{e}_{d+1}}(n\lambda^{(n)}t),$$

and, since this Poisson process is increasing in t, we have that for $t \leq T$,

$$\bar{N}^{(n)}(t) \leq \bar{N}^{(n)}(0) + \frac{1}{n}P_{\boldsymbol{e}_0+\boldsymbol{e}_{d+1}}(n\lambda^{(n)}T).$$

Now,

$$\mathbb{E}\left[\frac{1}{n}P_{\boldsymbol{e}_0+\boldsymbol{e}_{d+1}}(n\lambda^{(n)}T)\right] = \lambda^{(n)}T,$$

and, applying Chebyshev's inequality, we see that for any $C > 0$,

$$\mathbb{P}\left\{\left|\frac{1}{n}P_{\boldsymbol{e}_0+\boldsymbol{e}_{d+1}}(n\lambda^{(n)}T) - \lambda^{(n)}T\right| > C\right\} = \mathbb{P}\left\{|P_{\boldsymbol{e}_0+\boldsymbol{e}_{d+1}}(n\lambda^{(n)}T) - n\lambda^{(n)}T| > Cn\right\}$$
$$\leq \frac{\mathbb{E}\left[\left(P_{\boldsymbol{e}_0+\boldsymbol{e}_{d+1}}(n\lambda^{(n)}T) - n\lambda^{(n)}T\right)^2\right]}{C^2n^2} = \frac{\lambda^{(n)}T}{C^2n} \to 0$$

as $n \to \infty$. Thus, for any fixed $T > 0$, $\bar{N}^{(n)}(t)$ is bounded above and below on $[0, T]$ by *e.g.*, $\bar{N}^{(n)}(0)+\lambda^{(n)}T \pm 1$, with probability that approaches one as n tends to infinity.

Thus, for example,

$$\int_0^t \frac{\beta_i^{(n)}\bar{S}^{(n)}(s)\bar{I}_i^{(n)}(s)}{\bar{N}^{(n)}(s)}\,ds \leq \frac{\beta_i^{(n)}(\bar{N}^{(n)}(0) + \lambda^{(n)}T + 1)^2T}{\bar{N}^{(n)}(0) + \lambda^{(n)}T - 1},$$

and we may proceed exactly as above to conclude that fo $t \leq T$,

$$\frac{1}{n}P_{-\boldsymbol{e}_0+\boldsymbol{e}_i}\left(n\int_0^t \frac{\beta_i^{(n)}\bar{S}^{(n)}(s)\bar{I}_i^{(n)}(s)}{\bar{N}^{(n)}(s)}\,ds\right)$$

is bounded above with probability approaching 1 as $n \to \infty$, and similarly for the other Poisson processes, from which we conclude that there exists some constant C' such that

$$|\varepsilon_i^{(n)}(t)| \leq \frac{C'}{n^2}$$

with high probability.

8.2 Proof of Proposition 5

In this section, we will make the heuristic argument of Section 5.2 rigorous using the technique of coupling (see [2] for a very good introduction): we start by constructing birth and death processes that bound $I_1^{(n)}(t)$ above and below provided $\bar{S}^{(n)}(t)$ and $\bar{N}^{(n)}(t)$ remain within ε of the endemic equilibrium, and such that the upper and lower bounds approach one another as $\varepsilon \to 0$. Finally, we show that the probability that $\bar{S}^{(n)}(t)$ and $\bar{N}^{(n)}(t)$ depart a ε-neighbourhood of the endemic equilibrium before strain 2 has either successfully invaded or gone extinct goes to 0 as $n \to \infty$. Since ε is arbitrary, we recover the naïve branching process result.

8.2.1 Macroscopic Initial Frequencies

In this section we prove the following extinction of part (i) of Proposition 5, where the population is infected with $d \geq 2$ strains.

Proposition 11. *Suppose that $R_{0,1} > R_{0,i}$ for all $i > 1$. If $\bar{I}_1^{(n)}(0) \to I_1(0) > 0$, then all strains $i > 1$ will go extinct with high probability.*

In light of the results in Section 5.1, we might, without loss of generality, assume that at time $t = 0$, the process is in some neighbourhood of endemic fixed point $\bar{\boldsymbol{E}}^{\star,1} = (\bar{S}^{\star,1}, \bar{I}_1^{\star,1}, \ldots, \bar{I}_d^{\star,1}, \bar{N}^{\star,1})$, as defined by (S.13).

In particular, fix $\varepsilon > 0$ such that

$$1 < \frac{\bar{S}^{\star,1} - \varepsilon}{\bar{N}^{\star,1} + \varepsilon} < \frac{1}{R_{0,1}} = \frac{\bar{S}^{\star,1}}{\bar{N}^{\star,1}} < \frac{\bar{S}^{\star,1} + \varepsilon}{\bar{N}^{\star,1} - \varepsilon} < \frac{1}{R_{0,2}} < \frac{1}{R_{0,3}} < \cdots < \frac{1}{R_{0,d}}.$$

For reasons that will become transparent below, we will assume that

$$\|\bar{\boldsymbol{E}}^{(n)}(0) - \bar{\boldsymbol{E}}^{\star,1}\| < B\varepsilon.$$

for some constant $0 < B < 1$ that will be determined later.

Let

$$\tau_\varepsilon^{(n)} := \inf \left\{ t : \|\bar{\boldsymbol{E}}^{(n)}(t) - \bar{\boldsymbol{E}}^{\star,1}\| > \varepsilon \right\},$$

where we adopt the convention that $\tau_\varepsilon^{(n)} = \infty$ if $\|\bar{\boldsymbol{E}}^{(n)}(t) - \bar{\boldsymbol{E}}^{\star,1}\| < \varepsilon$ for all t.

Provided $t < \tau_\varepsilon^{(n)}$, we have that

$$\frac{\bar{S}^{\star,1} - \varepsilon}{\bar{N}^{\star,1} + \varepsilon} < \frac{\bar{S}^{(n)}(t)}{\bar{N}^{(n)}(t)} < \frac{\bar{S}^{\star,1} + \varepsilon}{\bar{N}^{\star,1} - \varepsilon}.$$

Next, fix $\eta > 0$ sufficiently small that

$$(\beta_i + \eta)\frac{\bar{S}^{\star,1} + \varepsilon}{\bar{N}^{\star,1} - \varepsilon} - (\delta + \alpha_i + \gamma_i - 3\eta) = \beta_i \left(\frac{\bar{S}^{\star,1} + \varepsilon}{\bar{N}^{\star,1} - \varepsilon} - \frac{1}{R_{0,i}} \right) + \eta \left(3 + \frac{\bar{S}^{\star,1} + \varepsilon}{\bar{N}^{\star,1} - \varepsilon} \right) < 0$$

Since $I_i^{(n)}(0) \to I_i(0)$ for $i > 1$ and $\beta_i^{(n)} \to \beta_i$, *etc.* we can assume that n is sufficiently large that

$$|\beta_i^{(n)} - \beta_i| < \eta, \quad |\delta^{(n)} - \delta| < \eta, \quad |\alpha_i^{(n)} - \alpha_i| < \eta, \quad \text{and} \quad |\gamma_i^{(n)} - \gamma_i| < \eta$$

for all $i > 1$.

Thus, for $t < \tau_\varepsilon^{(n)}$, the per-infective transmission rate for strain i satisfies

$$(\beta_i - \eta)\frac{\bar{S}^{\star,1} - \varepsilon}{\bar{N}^{\star,1} + \varepsilon} < \frac{\beta_i^{(n)} S^{(n)}(t-)}{N^{(n)}(t-)} < (\beta_i + \eta)\frac{\bar{S}^{\star,1} + \varepsilon}{\bar{N}^{\star,1} - \varepsilon}$$

whereas the total per-infective rate of removal of strain i satisfies

$$\delta + \alpha_i + \gamma_i + 3\eta > \delta^{(n)} + \alpha_i^{(n)} + \gamma_i^{(n)} > \delta + \alpha_i + \gamma_i - 3\eta.$$

Lemma 3. *Provided $t < \tau_\varepsilon^{(n)}$, the number of infectives of strain i is stochastically smaller* [3] *than the birth and death process $Z_i^+(t)$ with $Z_i^+(0) = I_i^{(n)}(0)$ and birth and death rates*

$$(\beta_i + \eta)\frac{\bar{S}^{\star,1} + \varepsilon}{\bar{N}^{\star,1} - \varepsilon} \quad \text{and} \quad \delta + \alpha_i + \gamma_i - 3\eta,$$

and stochastically greater than the birth and death process $Z_i^-(t)$ with $Z_i^-(0) = I_i^{(n)}(0)$ and birth and death rates

$$(\beta_i - \eta)\frac{\bar{S}^{\star,1} - \varepsilon}{\bar{N}^{\star,1} + \varepsilon} \quad \text{and} \quad \delta + \alpha_i + \gamma_i + 3\eta.$$

Proof. It suffices to construct coupled versions of $I_i^{(n)}(t)$, $Z_i^+(t)$ and $Z_i^-(t)$ such that

$$Z_i^+(t) \geq I_i^{(n)}(t) \geq Z_i^-(t).$$

We will do so inductively, at each step constructing the processes up to the next among the aggregated jump times of all three processes, which we denote

$$0 = \tau_0 < \tau_1 < \tau_2 < \cdots .$$

For our underlying probability space, we assume sequences of independent rate 1 exponential random variables $\mathcal{E}_k$ and independent uniformly distributed random variables $\mathcal{U}_k$ on $[0, 1]$, for $k = 1, 2, \ldots$.

Suppose that $I_i^{(n)}(t)$, $Z_i^+(t)$ and $Z_i^-(t)$ have been constructed up to τ_k (trivially true for $k = 0$). Note that

$$\rho_{k+1} := (\beta_i + \eta)\frac{\bar{S}^{\star,1} + \varepsilon}{\bar{N}^{\star,1} - \varepsilon} Z_k^+(\tau_k) + (\delta + \alpha_i + \gamma_i + 3) Z_k^+(\tau_k)$$

is an upper bound on the combined rate of all transitions for all three processes. Set

$$\tau_{k+1} = \frac{\mathcal{E}_{k+1}}{\rho_{k+1}},$$

so τ_{k+1} is a rate ρ_{k+1} exponential random variable. Next, for $t < \tau_{k+1}$ we set

$$I_i^{(n)}(t) = I_i^{(n)}(\tau_k), \quad Z_i^+(t) = Z_i^+(\tau_k) \quad \text{and} \quad Z_i^-(t) = Z_i^-(\tau_k).$$

[3] Given random variables X and X', we say that X is *stochastically smaller* than X', denoted $X \preceq X'$ if

$$\mathbb{P}\{X' \geq x\} \geq \mathbb{P}\{X \geq x\}.$$

Similarly, a stochastic process X is stochastically smaller than the process X' if $S(t) \preceq X'(t)$ for all $t \geq 0$. One defines *stochastically greater* analogously.

Finally, we set

$$
\begin{aligned}
&(I_i^{(n)}(\tau_{k+1}), Z_i^+(\tau_{k+1}), Z_i^-(\tau_{k+1}))\\
&= \begin{cases}
(I_i^{(n)}(\tau_k){+}1, Z_i^+(\tau_k){+}1, Z_i^-(\tau_k){+}1) & \text{if } \mathcal{U}_{k+1} \le \frac{(\beta_i-\eta)\frac{\bar{S}^{\star,1}-\varepsilon}{\bar{N}^{\star,1}+\varepsilon}}{\rho_{k+1}}\\
(I_i^{(n)}(\tau_k){+}1, Z_i^+(\tau_k){+}1, Z_i^-(\tau_k)) & \text{if } \frac{(\beta_i-\eta)\frac{\bar{S}^{\star,1}-\varepsilon}{\bar{N}^{\star,1}+\varepsilon}}{\rho_{k+1}} \le \mathcal{U}_{k+1} < \frac{\beta_i\frac{\bar{S}^{(n)}(t)}{\bar{N}^{(n)}(t)}}{\rho_{k+1}}\\
(I_i^{(n)}(\tau_k){+}1, Z_i^+(\tau_k), Z_i^-(\tau_k)) & \text{if } \frac{\beta_i\frac{\bar{S}^{(n)}(t)}{\bar{N}^{(n)}(t)}}{\rho_{k+1}} < \mathcal{U}_{k+1} \le \frac{(\beta_i+\eta)\frac{\bar{S}^{\star,1}+\varepsilon}{\bar{N}^{\star,1}-\varepsilon}}{\rho_{k+1}}\\
(I_i^{(n)}(\tau_k){-}1, Z_i^+(\tau_k){-}1, Z_i^-(\tau_k){-}1) & \text{if } \frac{(\beta_i+\eta)\frac{\bar{S}^{\star,1}+\varepsilon}{\bar{N}^{\star,1}-\varepsilon}}{\rho_{k+1}} < \mathcal{U}_{k+1} \le \frac{(\beta_i+\eta)\frac{\bar{S}^{\star,1}+\varepsilon}{\bar{N}^{\star,1}-\varepsilon}+\delta+\alpha_i+\gamma_i-3\eta}{\rho_{k+1}}\\
(I_i^{(n)}(\tau_k), Z_i^+(\tau_k){-}1, Z_i^-(\tau_k){-}1) & \text{if } \frac{(\beta_i+\eta)\frac{\bar{S}^{\star,1}+\varepsilon}{\bar{N}^{\star,1}-\varepsilon}+\delta+\alpha_i+\gamma_i-3\eta}{\rho_{k+1}} < \mathcal{U}_{k+1} \le \frac{(\beta_i+\eta)\frac{\bar{S}^{\star,1}+\varepsilon}{\bar{N}^{\star,1}-\varepsilon}+\delta^{(n)}+\alpha_i^{(n)}+\gamma_i^{(n)}}{\rho_{k+1}}\\
(I_i^{(n)}(\tau_k), Z_i^+(\tau_k), Z_i^-(\tau_k){-}1) & \text{if } \frac{(\beta_i+\eta)\frac{\bar{S}^{\star,1}+\varepsilon}{\bar{N}^{\star,1}-\varepsilon}+\delta^{(n)}+\alpha_i^{(n)}+\gamma_i^{(n)}}{\rho_{k+1}} < \mathcal{U}_{k+1} \le 1.
\end{cases}
\end{aligned}
$$

It is readily verified that the resulting processes have the correct jump rates. □

Remark 7. Note that given $\varepsilon > \varepsilon_1 > 0$, choosing $\eta > 0$ as before and $\eta_1 > 0$ analogously, we can similarly construct processes $Z_i^{+,1}(t)$ and $Z_i^{-,1}(t)$ such that

$$Z_i^+(t) \succeq Z_i^{+,1}(t) \succeq I_i^{(n)}(t) \succeq Z_i^{-,1}(t) \succeq Z_i^-(t),$$

etc. We shall apply this with a decreasing sequence of values $\varepsilon_n > 0$ below.

Now, our choice of η ensures that $Z_i^+(t)$ is subcritical for all $i > 1$. In particular, setting

$$\mu_i^+ := (\beta_i + \eta)\frac{\bar{S}^{\star,1} + \varepsilon}{\bar{N}^{\star,1} - \varepsilon} - (\delta + \alpha_i + \gamma_i - 3\eta) < 0,$$

we have

$$\mathbb{E}\left[Z_i^+(t)\right] = Z_i^+(0)e^{\mu_i^+ t},$$

and, for any sequence $t_n > \frac{1}{|\mu_i^+|}\ln n$, for all $i > 1$, we have, using Markov's inequality

$$\mathbb{P}\left\{Z_i^+(t_n) \ge 1\right\} \le \mathbb{E}\left[Z_i^+(t_n)\right] \le B\varepsilon n e^{\mu_i^+ t_n} \to 0$$

as $n \to \infty$. Thus, if we show that $\mathbb{P}\left\{\tau_\varepsilon^{(n)} > t_n\right\} \to 1$ as $n \to \infty$, we can conclude that all strains $i > 1$ vanish after before t_n with high probability.

To this end, we start by defining

$$\tau_{\varepsilon,i}^{(n)} := \inf\left\{t : |\bar{I}_i^{(n)}(t) - \bar{I}_i^{\star,1}| \ge \varepsilon n\right\},$$

for $i = 1, \ldots, d$. We define $\tau_{\varepsilon,0}^{(n)}$ and $\tau_{\varepsilon,d+1}^{(n)}$ similarly, replacing $\bar{I}_i$ by $\bar{S}$ or $\bar{N}$ respectively in the above

definition (again, $\tau_{\varepsilon,i}^{(n)} = \infty$ should the respective process never exceed εn). We then have

$$\tau_{\varepsilon}^{(n)} = \min_{i} \tau_{\varepsilon,i}^{(n)}.$$

We continue with a classical result for birth and death processes: for $i > 1$, let

$$a_i = \frac{\delta + \alpha_i + \gamma_i - 3\eta}{(\beta_i + \eta)\frac{\bar{S}^{\star,1}+\varepsilon}{\bar{N}^{\star,1}-\varepsilon}} > 1.$$

Then, a simple calculation shows that

$$\mathbb{E}\left[a_i^{Z_i^+(t)} \middle| Z_i^+(s)\right] = a_i^{Z_i^+(s)}$$

i.e., $a_i^{Z_i^+(t)}$ is a martingale. Let

$$\tau_{0,i}^{(n)} = \inf\left\{t : Z_i^+(t) = 0\right\} \quad \text{and} \quad \tau_i^{(n)} = \min\{\tau_{0,i}^{(n)}, \tau_{\varepsilon,i}^{(n)}\}.$$

We saw above that $\tau_{0,i}^{(n)}$ and thus $\tau_i^{(n)}$ are with high probability bounded above by any sequence $t_n > \frac{1}{\min_{i>1}|\mu_i^+|}\ln n$. Now,

$$a_i^{Z_i^+(0)} = \mathbb{E}\left[a_i^{Z_i^+(\tau_i^{(n)})}\right] = a_i^{\lfloor \varepsilon n \rfloor}\mathbb{P}\left\{\tau_{0,i}^{(n)} > \tau_{\varepsilon,i}^{(n)}\right\} + \left(1 - \mathbb{P}\left\{\tau_{0,i}^{(n)} > \tau_{\varepsilon,i}^{(n)}\right\}\right)$$

i.e.,

$$\mathbb{P}\left\{\tau_{0,i}^{(n)} > \tau_{\varepsilon,i}^{(n)}\right\} = \frac{a_i^{Z_i^+(0)} - 1}{a_i^{\lfloor \varepsilon n \rfloor} - 1} \geq \frac{a_i^{\lfloor B\varepsilon n \rfloor} - 1}{a_i^{\lfloor \varepsilon n \rfloor} - 1},$$

which converges to 0 as $n \to \infty$ for any $B < 1$. We thus have

$$\mathbb{P}\left\{\tau_{\varepsilon,i}^{(n)} > t_n\right\} \to 1$$

as $n \to \infty$.

For the remaining three values $\tau_{\varepsilon,i}^{(n)}$, $i = 0, 1, d+1$, we take a different approach, as the values $\bar{S}^{\star,1}$, $\bar{I}_1^{\star,1}$, and $\bar{N}^{\star,1}$ are all non-zero and a branching process approach is no longer appropriate. Instead, we recall the SDE representation of our process (Proposition 1).

Now $\bar{\boldsymbol{E}}^{\star,1}$ is a stable fixed point for the dynamical system $\dot{\bar{\boldsymbol{E}}} = \boldsymbol{F}(\bar{\boldsymbol{E}})$, so we may write

$$\boldsymbol{F}(\bar{\boldsymbol{E}}) = A(\bar{\boldsymbol{E}} - \bar{\boldsymbol{E}}^{\star,1}) + \boldsymbol{G}(\bar{\boldsymbol{E}} - \bar{\boldsymbol{E}}^{\star,1}),$$

where $A := \boldsymbol{DF}(\bar{\boldsymbol{E}}^{\star,1})$, the Jacobian of $\boldsymbol{F}(\boldsymbol{x})$ evaluated at the resident endemic equilibrium, is a stable matrix and

$$\left\|\boldsymbol{G}(\bar{\boldsymbol{E}})\right\| \leq M \left\|\bar{\boldsymbol{E}}\right\|^2$$

for some fixed $M > 0$.

Now, let

$$\Xi^{(n)}(t) := \bar{\boldsymbol{E}}^{(n)}(t) - \bar{\boldsymbol{E}}^{\star,1}.$$

Then, using Duhamel's principle, we have that

$$\begin{aligned}
\Xi^{(n)}(t) &= e^{tA}\Xi^{(n)}(0) + \int_0^t e^{(t-s)A}\boldsymbol{G}\left(\Xi^{(n)}(s)\right)\, ds \\
&\quad + \frac{1}{n}\int_0^t e^{(t-s)A}(\boldsymbol{e}_0+\boldsymbol{e}_{d+1})\, dM^{(n)}_{\boldsymbol{e}_0+\boldsymbol{e}_{d+1}}(t) - \frac{1}{n}\int_0^t e^{(t-s)A}(\boldsymbol{e}_0+\boldsymbol{e}_{d+1})\, dM^{(n)}_{-\boldsymbol{e}_0-\boldsymbol{e}_{d+1}}(t) \\
&\quad + \frac{1}{n}\sum_{i=1}^d \int_0^t e^{(t-s)A}(\boldsymbol{e}_i-\boldsymbol{e}_0)\, dM^{(n)}_{-\boldsymbol{e}_0+\boldsymbol{e}_i}(t) - \frac{1}{n}\sum_{i=1}^d \int_0^t e^{(t-s)A}(\boldsymbol{e}_i+\boldsymbol{e}_{d+1})\, dM^{(n)}_{-\boldsymbol{e}_i-\boldsymbol{e}_{d+1}}(t) \\
&\quad - \frac{1}{n}\sum_{i=1}^d \int_0^t e^{(t-s)A}\boldsymbol{e}_i\, dM^{(n)}_{-\boldsymbol{e}_i}(t) - \frac{1}{n}\int_0^t e^{(t-s)A}\boldsymbol{e}_{d+1}\, dM^{(n)}_{-\boldsymbol{e}_{d+1}}(t). \qquad \text{(S.43)}
\end{aligned}$$

Let $\alpha_1, \dots, \alpha_d$ denote the eigenvalues of A. It is a standard result (see *e.g.,* [30]) that, given any $\alpha < \min\{-\Re(\alpha_j) : \Re(\alpha_j) < 0\}$, there exists a constant C, depending on α such that, when restricted to E_s, we have

$$\|e^{(t-r)A}\| \leq Ce^{-\alpha(t-r)}.$$

Thus,

$$\begin{aligned}
\|\Xi^{(n)}(t)\| &\leq Ce^{-\alpha t}\|\Xi^{(n)}(0)\| + \int_0^t Ce^{-\alpha(t-s)}M\|\Xi^{(n)}(s)\|^2\, ds \\
&\quad + \frac{1}{n}\int_0^t Ce^{-\alpha(t-s)}\, dM^{(n)}_{\boldsymbol{e}_0+\boldsymbol{e}_{d+1}}(t) + \frac{1}{n}\int_0^t Ce^{-\alpha(t-s)}\, dM^{(n)}_{-\boldsymbol{e}_0-\boldsymbol{e}_{d+1}}(t) \\
&\quad + \frac{1}{n}\sum_{i=1}^d \int_0^t Ce^{-\alpha(t-s)}\, dM^{(n)}_{-\boldsymbol{e}_0+\boldsymbol{e}_i}(t) + \frac{1}{n}\sum_{i=1}^d \int_0^t Ce^{-\alpha(t-s)}\, dM^{(n)}_{-\boldsymbol{e}_i-\boldsymbol{e}_{d+1}}(t) \\
&\quad + \frac{1}{n}\sum_{i=1}^d \int_0^t Ce^{-\alpha(t-s)}\, dM^{(n)}_{-\boldsymbol{e}_i}(t) + \frac{1}{n}\int_0^t Ce^{-\alpha(t-s)}\, dM^{(n)}_{-\boldsymbol{e}_{d+1}}(t).
\end{aligned}$$

Now, fix a sequence $\ln n \ll t_n \ll n$, we observe that

$$\begin{aligned}
\mathbb{P}\left\{\tau_\varepsilon^{(n)} < t_n\right\} &= \mathbb{P}\left\{\sup_{t\leq\tau_\varepsilon^{(n)}\wedge t_n} \|\Xi^{(n)}(t)\| \geq \varepsilon\right\} \\
&\leq \mathbb{P}\left\{Ce^{-\alpha t}\|\Xi^{(n)}(0)\| \geq \frac{\varepsilon}{5+3d}\right\} + \mathbb{P}\left\{\sup_{t\leq\tau_\varepsilon^{(n)}} \int_0^t Ce^{-\alpha(t-s)}M\|\Xi^{(n)}(s)\|^2\, ds \geq \frac{\varepsilon}{5+3d}\right\} \\
&\quad + \mathbb{P}\left\{\frac{1}{n}\sup_{t\leq\tau_\varepsilon^{(n)}\wedge t_n} \int_0^t Ce^{-\alpha(t-s)}\, dM^{(n)}_{\boldsymbol{e}_0+\boldsymbol{e}_{d+1}}(s) \geq \frac{\varepsilon}{5+3d}\right\}
\end{aligned}$$

$$+\mathbb{P}\left\{\frac{1}{n}\sup_{t\le\tau_\varepsilon^{(n)}\wedge t_n}\int_0^t Ce^{-\alpha(t-s)}\,dM^{(n)}_{-\boldsymbol{e}_0-\boldsymbol{e}_{d+1}}(s)\ge\frac{\varepsilon}{5+3d}\right\}$$

$$+\sum_{i=1}^d\mathbb{P}\left\{\frac{1}{n}\sup_{t\le\tau_\varepsilon^{(n)}\wedge t_n}\int_0^t Ce^{-\alpha(t-s)}\,dM^{(n)}_{-\boldsymbol{e}_0+\boldsymbol{e}_i}(s)\ge\frac{\varepsilon}{5+3d}\right\}$$

$$+\sum_{i=1}^d\mathbb{P}\left\{\frac{1}{n}\sup_{t\le\tau_\varepsilon^{(n)}\wedge t_n}\int_0^t Ce^{-\alpha(t-s)}\,dM^{(n)}_{-\boldsymbol{e}_i-\boldsymbol{e}_{d+1}}(s)\ge\frac{\varepsilon}{5+3d}\right\}$$

$$+\sum_{i=1}^d\mathbb{P}\left\{\frac{1}{n}\sup_{t\le\tau_\varepsilon^{(n)}\wedge t_n}\int_0^t Ce^{-\alpha(t-s)}\,dM^{(n)}_{-\boldsymbol{e}_i}(s)\ge\frac{\varepsilon}{5+3d}\right\}$$

$$+\mathbb{P}\left\{\frac{1}{n}\sup_{t\le\tau_\varepsilon^{(n)}\wedge t_n}\int_0^t Ce^{-\alpha(t-s)}\,dM^{(n)}_{-\boldsymbol{e}_{d+1}}(s)\ge\frac{\varepsilon}{5+3d}\right\}$$

Now,

$$Ce^{-\alpha t}\|\Xi^{(n)}(0)\|\le CB\varepsilon$$

and

$$\sup_{t\le\tau_\varepsilon^{(n)}}\int_0^t Ce^{-\alpha(t-s)}M\|\Xi^{(n)}(s)\|^2\,ds\le\frac{CM}{\alpha}\varepsilon^2.$$

Thus, provided we choose $B<\frac{1}{C(5+3d)}$ and $\varepsilon<\frac{\alpha}{CM(5+3d)}$, then

$$\mathbb{P}\left\{Ce^{-\alpha t}\|\Xi^{(n)}(0)\|\ge\frac{\varepsilon}{5+3d}\right\}=\mathbb{P}\left\{\sup_{t\le\tau_\varepsilon^{(n)}}\int_0^t Ce^{-\alpha(t-s)}M\|\Xi^{(n)}(s)\|^2\,ds\ge\frac{\varepsilon}{5+3d}\right\}=0,$$

Finally, we turn to the integrals $\int_0^t e^{-\alpha(t-s)}\,dM^{(n)}_{\boldsymbol{l}}(s)$. Recall that each of the integrators $M^{(n)}_{\boldsymbol{l}}(t)$ takes the form

$$\tilde{P}_{\boldsymbol{l}}\left(n\int\Lambda_{\boldsymbol{l}}(\bar{\boldsymbol{E}}^{(n)}(t))\,ds\right),$$

for some continuous function $\Lambda_{\boldsymbol{l}}$. We will thus prove the generic lemma:

Lemma 4. *Let P be a Poisson process, $\Lambda:\mathbb{R}^{d+2}\to\mathbb{R}$ be continuous, and let*

$$M^{(n)}(t)=\tilde{P}\left(n\int\Lambda(\bar{\boldsymbol{E}}^{(n)}(t))\,ds\right).$$

then for any $\alpha>0$, any constant C, any sequence $t_n\ll n$, and $\tau_\varepsilon^{(n)}$ as above, we have

$$\mathbb{P}\left\{\frac{C}{n}\sup_{t\le\tau_\varepsilon^{(n)}\wedge t_n}\int_0^t e^{-\alpha(t-s)}\,dM^{(n)}(s)>R\right\}\to 0$$

as $n\to\infty$, for any fixed $R>0$.

Proof. We start by observing that for $t \le \tau_\varepsilon^{(n)}$, $\bar{\boldsymbol{E}}^{(n)}(t)) \in \overline{B_\varepsilon(\bar{\boldsymbol{E}}^{\star,1})}$, a compact set, and thus

$$\Lambda(\bar{\boldsymbol{E}}^{(n)}(t)) \le \overline{\Lambda}$$

for a constant $\overline{\Lambda} > 0$ depending on Λ, ε, and $\bar{\boldsymbol{E}}^{\star,1}$.

$$\begin{aligned}
\mathbb{P}\left\{\frac{C}{n}\sup_{t\le\tau_\varepsilon^{(n)}\wedge t_n}\int_0^t e^{-\alpha(t-s)}\,dM^{(n)}(s) > R\right\} &= \mathbb{P}\left\{\sup_{t\le\tau_\varepsilon^{(n)}\wedge t_n} e^{-\alpha t}\int_0^t e^{\alpha s}\,dM^{(n)}(s) > \frac{nR}{C}\right\} \\
&\le \mathbb{P}\left\{\sup_{t\le t_n} e^{-\alpha t}\int_0^{t\wedge\tau_\varepsilon^{(n)}} e^{\alpha s}\,dM^{(n)}(s) > \frac{nR}{C}\right\} \\
&\le \sum_{k=0}^{t_n-1}\mathbb{P}\left\{\sup_{k<t\le k+1} e^{-\alpha t}\int_0^{t\wedge\tau_\varepsilon^{(n)}} e^{\alpha s}\,dM^{(n)}(s) > \frac{nR}{C}\right\} \\
&\le \sum_{k=0}^{t_n-1}\mathbb{P}\left\{\sup_{k<t\le k+1} e^{-\alpha k}\int_0^{t\wedge\tau_\varepsilon^{(n)}} e^{\alpha s}\,dM^{(n)}(s) > \frac{nR}{C}\right\} \\
&= \sum_{k=0}^{t_n-1}\mathbb{P}\left\{\sup_{k<t\le k+1}\int_0^{t\wedge\tau_\varepsilon^{(n)}} e^{\alpha s}\,dM^{(n)}(s) > \frac{nR}{C}e^{\alpha k}\right\} \\
&\le \sum_{k=0}^{t_n-1}\mathbb{P}\left\{\sup_{t\le k+1}\int_0^{t\wedge\tau_\varepsilon^{(n)}} e^{\alpha s}\,dM^{(n)}(s) > \frac{nR}{C}e^{\alpha k}\right\}.
\end{aligned}$$

Now, applying Doob's inequality,

$$\begin{aligned}
\mathbb{P}\left\{\sup_{t\le k+1}\int_0^{t\wedge\tau_\varepsilon^{(n)}} e^{\alpha s}\,dM^{(n)}(s) > \frac{nR}{C}e^{\alpha k}\right\} &\le \frac{C^2}{n^2R^2}e^{-2\alpha k}\mathbb{E}\left[\left(\int_0^{k+1\wedge\tau_\varepsilon^{(n)}} e^{\alpha s}\,dM^{(n)}(s)\right)^2\right] \\
= \frac{C^2}{n^2R^2}e^{-2\alpha k}\mathbb{E}\left[\int_0^{k+1\wedge\tau_\varepsilon^{(n)}} e^{2\alpha s}n\Lambda(\bar{\boldsymbol{E}}^{(n)}(s))\,ds\right] &\le \frac{C^2}{nR^2}e^{-2\alpha k}\mathbb{E}\left[\int_0^{k+1} e^{2\alpha s}\overline{\Lambda}\,ds\right] \\
&\le \frac{C^2\overline{\Lambda}}{2\alpha nR^2}e^{-2\alpha k}\left(e^{2\alpha(k+1)}-1\right) \le \frac{C^2\overline{\Lambda}}{2\alpha nR^2}e^{2\alpha}
\end{aligned}$$

Thus,

$$\mathbb{P}\left\{\frac{C}{n}\sup_{t\le\tau_\varepsilon^{(n)}\wedge t_n}\int_0^t e^{-\alpha(t-s)}\,dM^{(n)}(s) > R\right\} \le \frac{C^2\overline{\Lambda}}{2\alpha nR^2}e^{2\alpha}t_n \to 0$$

as $n \to \infty$. □

Applying the lemma, we have

$$\mathbb{P}\left\{\tau_\varepsilon^{(n)} < t_n\right\} \to 0$$

and, with high probability, all strains $i > 1$ will vanish before t_n, and moreover, during this time, the process $\bar{\boldsymbol{E}}^{(n)}(t))$ will remain in $\overline{B_\varepsilon(\bar{\boldsymbol{E}}^{\star,1})}$.

8.2.2 Novel Strain in Small Number of Copies

We now consider the possibility that a new strain invades an established population. From the results of the previous section, we see that generically, the population will eventually be in an ε-neighbourhood of the fixed point $\bar{\boldsymbol{E}}^{\star,1}$ for arbitrary $\varepsilon > 0$, and that $I_i^{(n)}(t) \equiv 0$ for all $i > 1$.

If the new strain has reproductive number less than $R_{0,1}$, then the arguments of the preceding section apply directly, and we can conclude that the novel strain will very rapidly go extinct.

If, however, it has higher reproductive number, the invading strain now has a non-zero probability of establishing itself and replacing the resident strain. In this section, we adapt the techniques above to deal with this case (by the above, we may take $d = 2$).

We start by defining the quantities $\tau_{\varepsilon,i}^{(n)}$ and $\tau_{\varepsilon}^{(n)}$ as before, with the understanding that $\tau_{\varepsilon,1}^{(n)} = \infty$ if the new strain goes extinct before hitting εn.

We now fix $\varepsilon > 0$ such that

$$\frac{1}{R_{0,1}} < \frac{\bar{S}^{\star,1} - \varepsilon}{\bar{N}^{\star,1} + \varepsilon} < \frac{1}{R_{0,2}} = \frac{\bar{S}^{\star,1}}{\bar{N}^{\star,1}} < \frac{\bar{S}^{\star,1} + \varepsilon}{\bar{N}^{\star,1} - \varepsilon},$$

and $\eta > 0$ sufficiently small that

$$\begin{aligned}
\mu_2^+ := (\beta_2 + \eta)\frac{\bar{S}^{\star,1} + \varepsilon}{\bar{N}^{\star,1} - \varepsilon} - (\delta + \alpha_2 + \gamma_2 - 3\eta) \\
> \mu_2^- := (\beta_2 - \eta)\frac{\bar{S}^{\star,1} - \varepsilon}{\bar{N}^{\star,1} + \varepsilon} - (\delta + \alpha_2 + \gamma_2 + 3\eta) \\
= \beta_2\left(\frac{\bar{S}^{\star,1} - \varepsilon}{\bar{N}^{\star,1} + \varepsilon} - \frac{1}{R_{0,2}}\right) + \eta\left(\frac{\bar{S}^{\star,1} - \varepsilon}{\bar{N}^{\star,1} + \varepsilon} - 3\right) > 0,
\end{aligned}$$

and suppose that n is sufficiently large that $|\beta_2^{(n)} - \beta_2| < \eta$, *etc.*

Again, provided $t < \tau_{\varepsilon}^{(n)}$, we have that

$$\frac{\bar{S}^{\star,1} - \varepsilon}{\bar{N}^{\star,1} + \varepsilon} < \frac{\bar{S}^{(n)}(t)}{\bar{N}^{(n)}(t)} < \frac{\bar{S}^{\star,1} + \varepsilon}{\bar{N}^{\star,1} - \varepsilon},$$

and thus, if $Z^+(t)$ and $Z^-(t)$ are birth and death processes with birth and death rates

$$(\beta_2 + \eta)\frac{\bar{S}^{\star,1} + \varepsilon}{\bar{N}^{\star,1} - \varepsilon} \quad \text{and} \quad \delta + \alpha_2 + \gamma_2 - 3\eta$$

and

$$(\beta_2 - \eta)\frac{\bar{S}^{\star,1} - \varepsilon}{\bar{N}^{\star,1} + \varepsilon} \quad \text{and} \quad \delta + \alpha_2 + \gamma_2 + 3\eta$$

respectively, then both $Z^+(t)$ and $Z^-(t)$ are supercritical with Malthusian parameters μ_2^+ and μ_2^-, respectively, and

$$Z^-(t) < I_2^{(n)}(t) < Z^+(t)$$

stochastically for $t < \tau_\varepsilon^{(n)}$.

Now, set

$$\overline{q}_2 := \frac{\delta + \alpha_2 + \gamma_2 - 3\eta}{(\beta_2 + \eta)\frac{\bar{S}^{\star,1}+\varepsilon}{\bar{N}^{\star,1}-\varepsilon}} < \underline{q}_2 := \frac{\delta + \alpha_2 + \gamma_2 + 3\eta}{(\beta_2 - \eta)\frac{\bar{S}^{\star,1}-\varepsilon}{\bar{N}^{\star,1}+\varepsilon}} < 1.$$

Classical results for birth and death processes (*e.g.,* [1]) tell us that $Z^+(t)$ and $Z^-(t)$ will hit 0 in finite time with probability $\overline{q}_2^{Z^+(0)}$ and $\underline{q}_2$ respectively, and will grow indefinitely otherwise, and, moreover, that there exist random variables $\overline{W}$ and $\underline{W}$ taking values on $[0,\infty)$ such that

$$\mathbb{P}\{\overline{W} = 0\} = \overline{q}_2^{Z^+(0)} \quad \text{and} \quad \mathbb{P}\{\underline{W} = 0\} = \underline{q}_2^{Z^-(0)}$$

and

$$e^{-\mu_2^+ t} Z^+(t) \to \overline{W} \quad \text{and} \quad e^{-\mu_2^- t} Z^-(t) \to \underline{W} \tag{S.44}$$

both almost surely and in L^2.

Now, as before, fix $\ln n \ll t_n \ll n$. Taking logarithms in (S.44), we see that for almost all $\omega \notin \{\overline{W} = 0\}$, we have

$$\frac{\ln Z^-(\omega, t_n)}{t_n} \to \mu_2^- .$$

Now, if $Z^-(t_n) \leq \varepsilon n$ then the right hand side converges to 0. Moreover, if $I_2^{(n)}(t) \leq \varepsilon n$, then necessarily $Z^-(t_n) \leq \varepsilon n$, and we conclude that

$$\limsup_{n\to\infty} \mathbb{P}\left\{t_n < \tau_\varepsilon^{(n)}\right\} \leq \mathbb{P}\{\underline{W} = 0\} = \underline{q}_2^{Z^-(0)}.$$

In particular, if we can establish that with high probability $\tau_\varepsilon^{(n)} = \tau_{\varepsilon,2}^{(n)}$, then this gives a lower bound on the probability that the novel strain invades, as the latter is the probability that $\tau_\varepsilon^{(n)}$ is finite.

Now,

$$\mathbb{P}\left\{\tau_\varepsilon^{(n)} < \tau_{\varepsilon,2}^{(n)}\right\} = \mathbb{P}\left\{\tau_\varepsilon^{(n)} < \tau_{\varepsilon,2}^{(n)}; \tau_\varepsilon^{(n)} < t_n\right\} + \mathbb{P}\left\{\tau_\varepsilon^{(n)} < \tau_{\varepsilon,2}^{(n)}; \tau_\varepsilon^{(n)} > t_n\right\}.$$

We have already established that the latter is bounded above by $\underline{q}_1^{Z^-(0)}$ as $n \to \infty$. The former follows almost exactly as the proof that $\mathbb{P}\left\{\tau_\varepsilon^{(n)} < t_n\right\} \to 0$ of the previous section; $\bar{\boldsymbol{E}}^{\star,1}$ is now a hyperbolic fixed point rather than a stable fixed point, and A has a positive eigenvalue, but the stable manifold of $\bar{\boldsymbol{E}}^{\star,1}$ coincides with the subset of $\mathbb{R}^{d+2}$ with $x_1 = 0$. In particular, we may decompose

$$\mathbb{R}^{d+2} = E_S \oplus E_X,$$

where $E_S = \{x_1 = 0\}$ and E_X are A invariant subspaces, corresponding to the sum of the generalised eigenspaces for eigenvalues with negative and positive real parts respectively. We will write P_S and P_X for the corresponding projections (*i.e.,* P_S has image E_S and kernel E_X, and oppositely for P_X), and note that both P_S and P_X commute with A. Proceeding exactly as previously, we define

$$\Xi^{(n)}(t) := \bar{\boldsymbol{E}}^{(n)}(t) - \bar{\boldsymbol{E}}^{\star,1}$$

and let $\Xi_S^{(n)}(t) = P_S \Xi^{(n)}(t)$ denote it's projection onto the stable subspace. Then, if

$$\tau_{\varepsilon,S}^{(n)} = \tau_{\varepsilon,0}^{(n)} \wedge \tau_{\varepsilon,2}^{(n)} \wedge \tau_{\varepsilon,3}^{(n)}$$

then

$$\mathbb{P}\left\{\tau_\varepsilon^{(n)} < \tau_{\varepsilon,2}^{(n)}; t_n < \tau_\varepsilon^{(n)}\right\} = \mathbb{P}\left\{\tau_{\varepsilon,S}^{(n)} = \tau_\varepsilon^{(n)}; t_n < \tau_\varepsilon^{(n)}\right\} = \mathbb{P}\left\{\sup_{t \leq \tau_\varepsilon^{(n)} \wedge t_n} \|\Xi_S^{(n)}(t)\| \geq \varepsilon\right\}.$$

Applying the arguments of the previous section, using the equation for $\Xi_S^{(n)}(t)$ obtained by letting P_S act on both sides of (S.43), one obtains almost identically that

$$\mathbb{P}\left\{\sup_{t \leq \tau_\varepsilon^{(n)} \wedge t_n} \|\Xi_S^{(n)}(t)\| \geq \varepsilon\right\} \to 0$$

as $n \to \infty$.

Now, note that for any $\omega \in \{\overline{W} = 0\}$, we must have $Z^+(\omega, t) < \varepsilon n$ for all t, for some sufficiently large n, so $\omega \in \{\tau_\varepsilon^{(n)} < \tau_{\varepsilon,2}^{(n)}\}$. Thus we have

$$\overline{q}_1^{Z^+(0)} = \mathbb{P}\{\overline{W} = 0\} \leq \liminf_{n\to\infty} \mathbb{P}\left\{\tau_\varepsilon^{(n)} < \tau_{\varepsilon,1}^{(n)}\right\}$$

Finally, we notice that as $\varepsilon \to 0$ (and thus $\eta \to 0$ also,) both $\overline{q}_2$ and $\underline{q}_2$ approach

$$q_2 := \frac{\delta + \alpha_2 + \gamma_2}{\beta_2 \frac{\bar{S}^{\star,1}}{\bar{N}^{\star,1}}} = \frac{R_{0,1}}{R_{0,2}}.$$

Since the choice of ε was arbitrary in our definition of invasion, we conclude that the probability of successful invasion of the new strain 1 is

$$1 - \left(\frac{R_{0,1}}{R_{0,2}}\right)^{I_2(0)}. \tag{S.45}$$

Once this has happened, as we note above, Kurtz's deterministic approximation is applicable, and with high probability, the system will approach any arbitrarily small neighbourhood of the endemic fixed point for the new strain 1, at which point, by the argument above, the former resident strain, strain 2, goes extinct with probability approaching 1 as $n \to \infty$.

8.3 Proof of Proposition 10

We start with a lemma:

Lemma 5. *Suppose that $\alpha \in [\alpha_{min}, \alpha_{max}]$. Then,*

$$\int_{\alpha_{min}}^{\alpha_{max}} K_\varepsilon(\alpha, \alpha' - \alpha)(\alpha' - \alpha)^k \, d\alpha' = \begin{cases} \varepsilon^k \int_0^\infty K(\alpha, z) z^k \, dz + o(\varepsilon^k) & \text{if } \alpha = \alpha_{min}, \\ \varepsilon^k \int_{-\infty}^0 K(\alpha, z) z^k \, dz + o(\varepsilon^k) & \text{if } \alpha = \alpha_{max}, \text{ and} \\ \int_{-\infty}^\infty K(\alpha, z) z^k \, dz + o(\varepsilon^k) & \text{if } \alpha_{min} < \alpha < \alpha_{max} \end{cases}$$

Proof. Making a change of variable, we have

$$\begin{aligned} \int_{\alpha_{\min}}^{\alpha_{\max}} K_\varepsilon(\alpha, \alpha' - \alpha)(\alpha' - \alpha)^k \, d\alpha' &= \int_{\alpha_{\min}}^{\alpha_{\max}} K\left(\alpha, \frac{\alpha' - \alpha}{\varepsilon}\right) (\alpha' - \alpha)^k \frac{d\alpha'}{\varepsilon} \\ &= \varepsilon^k \int_{\frac{\alpha_{\min} - \alpha}{\varepsilon}}^{\frac{\alpha_{\max} - \alpha}{\varepsilon}} K(\alpha, z) z^k \, dz, \end{aligned}$$

and $\frac{\alpha_{\min} - \alpha}{\varepsilon}$ and $\frac{\alpha_{\max} - \alpha}{\varepsilon}$ tend to $-\infty$ and $+\infty$ respectively as $\varepsilon \to 0$, unless $\alpha = \alpha_{\min}$ or $\alpha = \alpha_{\max}$. By assumption, the integrals converge, so the tails must vanish as $\varepsilon \to 0$. □

Taylor expanding $s(\alpha, \alpha')\,(f(\alpha') - f(\alpha))$ in α' about α, we have

$$\begin{aligned} \hat{\mathcal{L}}_\varepsilon f(\alpha) = \frac{n}{\varepsilon^2} \eta(\alpha) I_{\text{eq}}(\alpha) \int_{\alpha_{\min}}^{\alpha_{\max}} K_\varepsilon(\alpha, \alpha' - \alpha) \big[s(\alpha, \alpha)(\alpha - \alpha') f'(\alpha) \\ + \left(\frac{\partial s}{\partial \alpha'}(\alpha, \alpha) f'(\alpha) + \frac{1}{2} s(\alpha, \alpha') f''(\alpha) \right) (\alpha - \alpha')^2 + \cdots \Big] \, d\alpha' \end{aligned}$$

From the Lemma, we have

$$\begin{aligned} \hat{\mathcal{L}}_\varepsilon f(\alpha_{\min}) = \frac{n}{\varepsilon} \eta(\alpha_{\min}) I_{\text{eq}}(\alpha_{\min}) s(\alpha_{\min}, \alpha_{\min}) f'(\alpha_{\min}) \int_0^\infty K(\alpha_{\min}, z) z \, dz \\ + \Bigg(n\eta(\alpha_{\min}) I_{\text{eq}}(\alpha_{\min}) \frac{\partial s}{\partial \alpha'}(\alpha_{\min}, \alpha_{\min}) f'(\alpha_{\min}) \\ + \frac{n}{2} \eta(\alpha_{\min}) I_{\text{eq}}(\alpha_{\min}) s(\alpha_{\min}, \alpha_{\min}) f''(\alpha_{\min}) \Bigg) \int_0^\infty K(\alpha_{\min}, z) z^2 \, dz + o(\varepsilon), \\ \hat{\mathcal{L}}_\varepsilon f(\alpha_{\max}) = \frac{n}{\varepsilon} \eta(\alpha_{\max}) I_{\text{eq}}(\alpha_{\max}) s(\alpha_{\max}, \alpha_{\max}) f'(\alpha_{\max}) \int_{-\infty}^0 K(\alpha_{\max}, z) z \, dz \\ + \Bigg(n\eta(\alpha_{\max}) I_{\text{eq}}(\alpha_{\max}) \frac{\partial s}{\partial \alpha'}(\alpha_{\max}, \alpha_{\max}) f'(\alpha_{\max}) \\ \frac{n}{2} \eta(\alpha_{\max}) I_{\text{eq}}(\alpha_{\max}) s(\alpha_{\max}, \alpha_{\max}) f''(\alpha_{\max}) \Bigg) \int_{-\infty}^0 K(\alpha_{\max}, z) z^2 \, dz + o(\varepsilon), \end{aligned}$$

and

$$\hat{\mathcal{L}}_\varepsilon f(\alpha) = n\eta(\alpha) I_{\text{eq}}(\alpha) \nu(\alpha) \frac{\partial s}{\partial \alpha'}(\alpha, \alpha) f'(\alpha) + \frac{n}{2} \eta(\alpha) I_{\text{eq}}(\alpha) \nu(\alpha) s(\alpha, \alpha) f''(\alpha) + o(\varepsilon).$$

for $\alpha \in (\alpha_{\min}, \alpha_{\max})$.

We first note that to have a well-posed (finite) limit as $\varepsilon \to 0$, we must have $f'(\alpha_{\min}) = f'(\alpha_{\max}) = 0$, so that the first term, which is otherwise of order $\mathcal{O}(\varepsilon^{-1})$, vanishes.

To deal with the discontinuities at $\alpha_{\min}$ and $\alpha_{\max}$, suppose we have f such that $f'(\alpha_{\min}) = f'(\alpha_{\max}) = 0$. Choose $h \in C^2[\alpha_{\min}, \alpha_{\max}]$ such that $h'(\alpha_{\min}) = \frac{1}{2} f''(\alpha_{\min}) \frac{\int_{-\infty}^{0} K(\alpha_{\min}, z) z^2\, dz}{\int_{0}^{\infty} K(\alpha_{\min}, z) z\, dz}$ and $h'(\alpha_{\max}) = \frac{1}{2} f''(\alpha_{\max}) \frac{\int_{0}^{\infty} K(\alpha_{\max}, z) z^2\, dz}{\int_{-\infty}^{0} K(\alpha_{\max}, z) z\, dz}$, and let $f_\varepsilon = f + \varepsilon h$, then

$$\hat{\mathcal{L}}_\varepsilon f(\alpha_{\min}) = \frac{n}{2} \eta(\alpha_{\min}) I_{\text{eq}}(\alpha_{\min}) \nu(\alpha_{\min}) s(\alpha_{\min}, \alpha_{\min}) f''(\alpha_{\min}) + o(\varepsilon),$$
$$\hat{\mathcal{L}}_\varepsilon f(\alpha_{\max}) = \frac{n}{2} \eta(\alpha_{\max}) I_{\text{eq}}(\alpha_{\max}) \nu(\alpha_{\max}) s(\alpha_{\max}, \alpha_{\max}) f''(\alpha_{\max}) + o(\varepsilon),$$

so that $\hat{\mathcal{L}}_\varepsilon f_\varepsilon(\alpha) \to \hat{\mathcal{L}} f(\alpha)$, $f_\varepsilon(\alpha) \to f(\alpha)$, for all $\alpha \in [\alpha_{\min}, \alpha_{\max}]$, and weak convergence follows by Corollary 8.7 in Chapter 4 of [14]

References

[1] K. B. Athreya and P. Ney. *Branching processes.* Springer-Verlag, Berlin, 1972.

[2] F. Ball. Coupling methods in epidemic theory. In D. Mollison, editor, *Epidemic Models: Their Structure and Relation to Data*, pages 34–52. Cambridge University Press, 1995.

[3] M. S. Bartlett. Stochastic processes. *Cambridge University Press*, 1955.

[4] C. M. Bender and S. A. Orszag. *Advanced Mathematical Methods for Scientists and Engineers.* McGraw-Hill, New York, 1978.

[5] P. Billingsley. *Convergence of Probability Measures.* Wiley, New York, 1968.

[6] H. J. Bremermann and H. R. Thieme. A competitive exclusion principle for pathogen virulence. *Journal of Mathematical Biology*, 27(2):179–190, 1989.

[7] N. Champagnat. A microscopic interpretation for adaptive dynamics trait substitution sequence models. *Stoch. Proc. Appl.*, 116(8):1127–1160, 2006.

[8] N. Champagnat, R. Ferrière, and G. Ben Arous. The canonical equation of adaptive dynamics: a mathematical view. *Selection*, 2:71–81, 2001.

[9] N. Champagnat and A. Lambert. Evolution of discrete populations and the canonical diffusion of adaptive dynamics. *Ann. Appl. Prob.*, 17(1):102–155, 2007.

[10] U. Dieckmann and R. Law. The dynamical theory of coevolution: A derivation from stochastic ecological processes. *J. Math. Biol.*, 34:579–612, 1996.

[11] R. Durrett. *Probability Models for DNA Sequence Evolution.* Springer, New York, 2nd edition, 2009.

[12] A. Erdélyi. *Asymptotic expansions.* Dover Publications, 1956.

[13] A. M. Etheridge. Some mathematical models from population genetics. In J. Picard, editor, *École d'Été de Probabilités de Saint-Flour, XXXIX—2009*, volume 2012 of *Lecture Notes in Math.*, Berlin Heidelberg, 2011. Springer.

[14] S. N. Ethier and T. G. Kurtz. *Markov Processes: Characterization and Convergence.* John Wiley and Sons, New York, 1986.

[15] W. J. Ewens. *Mathematical Population Genetics.* Springer-Verlag,, Berlin/New York, 1979.

[16] W. Feller. Diffusion processes in one dimension. *Trans. Amer. Math. Soc.*, 77(1):1–31, 1954.

[17] E. J. Hinch. *Perturbation Methods.* Cambridge University Press, Cambridge, 1991.

[18] M. W. Hirsch and S. Smale. *Differential Equations, Dynamical Systems, and Linear Algebra.* Academic Press, New York, 1974.

[19] G. S. Katzenberger. Solutions of a stochastic differential equation forced onto a manifold by a large drift. *Ann. Probab.*, 19(4):1587–1628, 1991.

[20] J. Kevorkian and J. D. Cole. *Multiple Scale and Singular Perturbation Methods.* Springer-Verlag New York Inc., New York, 1996.

[21] T. G. Kurtz. Solutions of ordinary differential equations as limits of pure jump Markov processes. *J. Appl. Prob.*, 7(1):pp. 49–58, 1970.

[22] T. G. Kurtz. Limit theorems for sequences of jump Markov processes approximating ordinary differential processes. *J. Appl. Prob.*, 8(2):344–356, 1971.

[23] T. G. Kurtz. Strong approximation theorems for density dependent Markov chains. *Stochastic Processes Appl.*, 6(3):223 – 240, 1978.

[24] T. G. Kurtz. *Approximation of Population Processes*, volume 36. Society for Industrial and Applied Mathematics, Philadelphia, 1981.

[25] J. A. J. Metz, R. M. Nisbet, and S. A. H. Geritz. How should we define 'fitness' for general ecological scenarios? *Trends Ecol. Evol.*, 7(6):198–202, 1992.

[26] S. P. Otto and T. Day. *A biologist's guide to mathematical modeling in ecology and evolution.* Princeton University Press, 2007.

[27] T. L. Parsons and T. Rogers. Dimension reduction for stochastic dynamical systems forced onto a manifold by large drift: a constructive approach with examples from theoretical biology. *J. Phys. A: Math. Theor.*, 50(41):415601, 2017.

[28] P. K. Pollett. On a model for interference between searching insect parasites. *J. Austral. Math. Soc. Ser. B*, 32(02):133–150, 1990.

[29] P. E. Protter. *Stochastic Integration and Differential Equations – a new approacg.* Springer, Berlin, 2nd edition, 2004.

[30] G. Teschl. *Ordinary differential equations and dynamical systems*, volume v. 140 of *Graduate studies in mathematics.* American Mathematical Society, Providence, R.I., 2012.

[31] N. G. van Kampen. *Stochastic Processes in Physics and Chemistry.* Elsevier, Amsterdam, San Diego, Oxford, London, 1992.

[32] C. Vargas-De-León. On the global stability of SIS, SIR and SIRS epidemic models with standard incidence. *Chaos, Solitons & Fractals*, 44(12):1106–1110, 2011.

[33] E. P. Wigner. The unreasonable effectiveness of mathematics in the natural sciences. *Commun. Pure Appl. Math*, 13(1):1–14, 1960.
